# Medical cannabinoids: a pharmacology-based systematic review and meta-analysis for all relevant medical indications

**DOI:** 10.1186/s12916-022-02459-1

**Published:** 2022-08-19

**Authors:** Ainhoa Bilbao, Rainer Spanagel

**Affiliations:** 1 Behavioral Genetics Research Group, Central Institute of Mental Health, Faculty of Medicine Mannheim, University of Heidelberg, Mannheim, Germany; 2 Institute of Psychopharmacology, Central Institute of Mental Health, Faculty of Medicine Mannheim, University of Heidelberg, Mannheim, Germany

**Keywords:** Cannabinoids, Pharmacology, Medical conditions, Neuropsychiatry, Clinical trial, Efficacy, Adverse events

## Abstract

**Background:**

Medical cannabinoids differ in their pharmacology and may have different treatment effects. We aimed to conduct a pharmacology-based systematic review (SR) and meta-analyses of medical cannabinoids for efficacy, retention and adverse events.

**Methods:**

We systematically reviewed (registered at PROSPERO: CRD42021229932) eight databases for randomized controlled trials (RCTs) of dronabinol, nabilone, cannabidiol and nabiximols for chronic pain, spasticity, nausea /vomiting, appetite, ALS, irritable bowel syndrome, MS, Chorea Huntington, epilepsy, dystonia, Parkinsonism, glaucoma, ADHD, anorexia nervosa, anxiety, dementia, depression, schizophrenia, PTSD, sleeping disorders, SUD and Tourette. Main outcomes and measures included patient-relevant/disease-specific outcomes, retention and adverse events. Data were calculated as standardized mean difference (SMD) and ORs with confidence intervals (CI) via random effects. Evidence quality was assessed by the Cochrane Risk of Bias and GRADE tools.

**Results:**

In total, 152 RCTs (12,123 participants) were analysed according to the type of the cannabinoid, outcome and comparator used, resulting in 84 comparisons. Significant therapeutic effects of medical cannabinoids show a large variability in the grade of evidence that depends on the type of cannabinoid. CBD has a significant therapeutic effect for epilepsy (SMD − 0.5[CI − 0.62, − 0.38] high grade) and Parkinsonism (− 0.41[CI − 0.75, − 0.08] moderate grade). There is moderate evidence for dronabinol for chronic pain (− 0.31[CI − 0.46, − 0.15]), appetite (− 0.51[CI − 0.87, − 0.15]) and Tourette (− 1.01[CI − 1.58, − 0.44]) and moderate evidence for nabiximols on chronic pain (− 0.25[− 0.37, − 0.14]), spasticity (− 0.36[CI − 0.54, − 0.19]), sleep (− 0.24[CI − 0.35, − 0.14]) and SUDs (− 0.48[CI − 0.92, − 0.04]). All other significant therapeutic effects have either low, very low, or even no grade of evidence. Cannabinoids produce different adverse events, and there is low to moderate grade of evidence for this conclusion depending on the type of cannabinoid.

**Conclusions:**

Cannabinoids are effective therapeutics for several medical indications if their specific pharmacological properties are considered. We suggest that future systematic studies in the cannabinoid field should be based upon their specific pharmacology.

**Supplementary Information:**

The online version contains supplementary material available at 10.1186/s12916-022-02459-1.

## Background

There is a worldwide growing interest and investments in using medical cannabinoids for the treatment of numerous diseases. Furthermore, in 2020, the United Nations (UN) finally recognized the medical value of cannabinoids and removed cannabis from Schedule IV of the 1961 Single Convention on Narcotic Drugs. This allows, in a less restricted manner, the use of medical cannabinoids. It is therefore of critical importance to thoroughly review the grade of evidence of the effectiveness of medical cannabinoids to inform policy and clinical decisions.

Previous systematic reviews have been limited in their coverage of all relevant diseases, but most importantly primarily ignored the fact that medical cannabinoid products—a term that encompasses all plant-derived and synthetic derivatives—differ in their pharmacology [1–5]. The synthetic cannabinoids dronabinol, which is ( −)-*trans*-Δ^9^-tetrahydrocannabinol (THC) (Marinol® and Syndros®), and nabilone—a synthetic cannabinoid with structural similarities to THC (Cesamet®), are partial agonists at the cannabinoid receptor 1 (CB1) and with somehow lower affinity at CB2 receptors [6]. Both cannabinoids have indications as appetite stimulants, antiemetics, cannabis addiction, sleep apnea and analgesics and are approved by the FDA for HIV/AIDS-induced loss of appetite and chemotherapy-induced nausea and vomiting. Cannabidiol (CBD; Epidolex®) acts as a negative allosteric modulator at CB1 receptors [7] and also acts at several other receptors, such as CB2 receptors, serotonin 1A receptors, opioid receptors and several ligand-gated ion channels [8]; it represents the only CBD formulation approved by both USA and Europe for the treatment of seizures associated with Dravet syndrome, Lennox-Gastaut syndrome or tuberous sclerosis complex. Nabiximols, a cannabis-derived extract that contains equal quantities of THC and CBD (Sativex®), was approved in 2010 in the UK for symptoms associated to MS, and exported to more than 28 countries from Asia, Africa, the Middle East, Europe (Spain, Czech Republic, Germany, Denmark, Sweden, Italy, Austria, France, Poland) and Canada. Moreover, plant-derived medical cannabis contains almost 150 phytocannabinoids, though most of them have neither been isolated nor pharmacologically characterized [9]. THC and CBD can vary largely in concentrations across different medical cannabis products and can thereby differ in their pharmacological properties. Therefore, a systematic review (SR) that does not consider the different pharmacological properties of medical cannabinoids can be misleading.

The aim of this SR and meta-analysis is to examine possible therapeutic differences for medical cannabinoids in all relevant medical conditions.

## Results

**Fig. 1:**
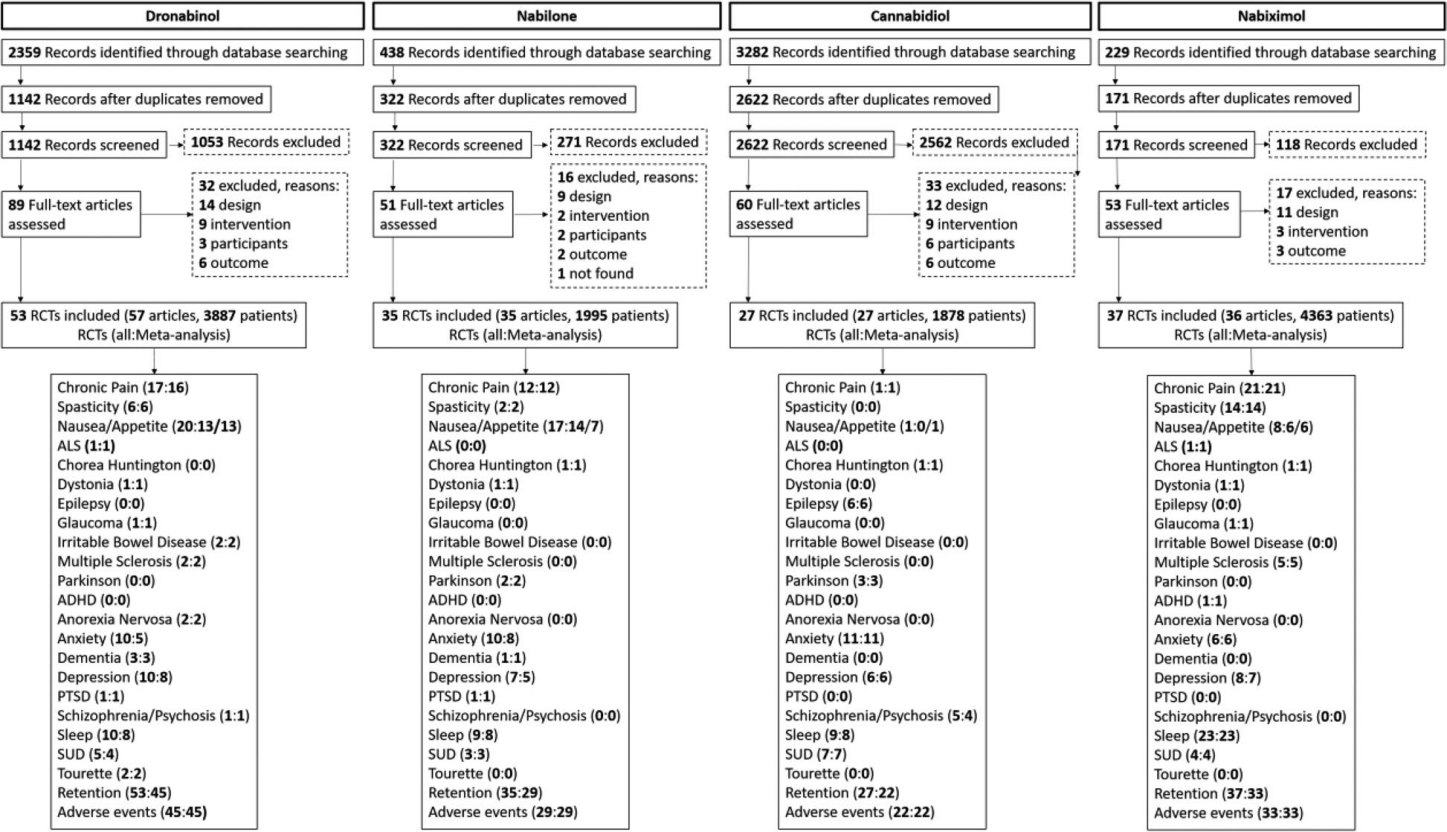
PRISMA flowchart of the studies

**Fig. 2:**
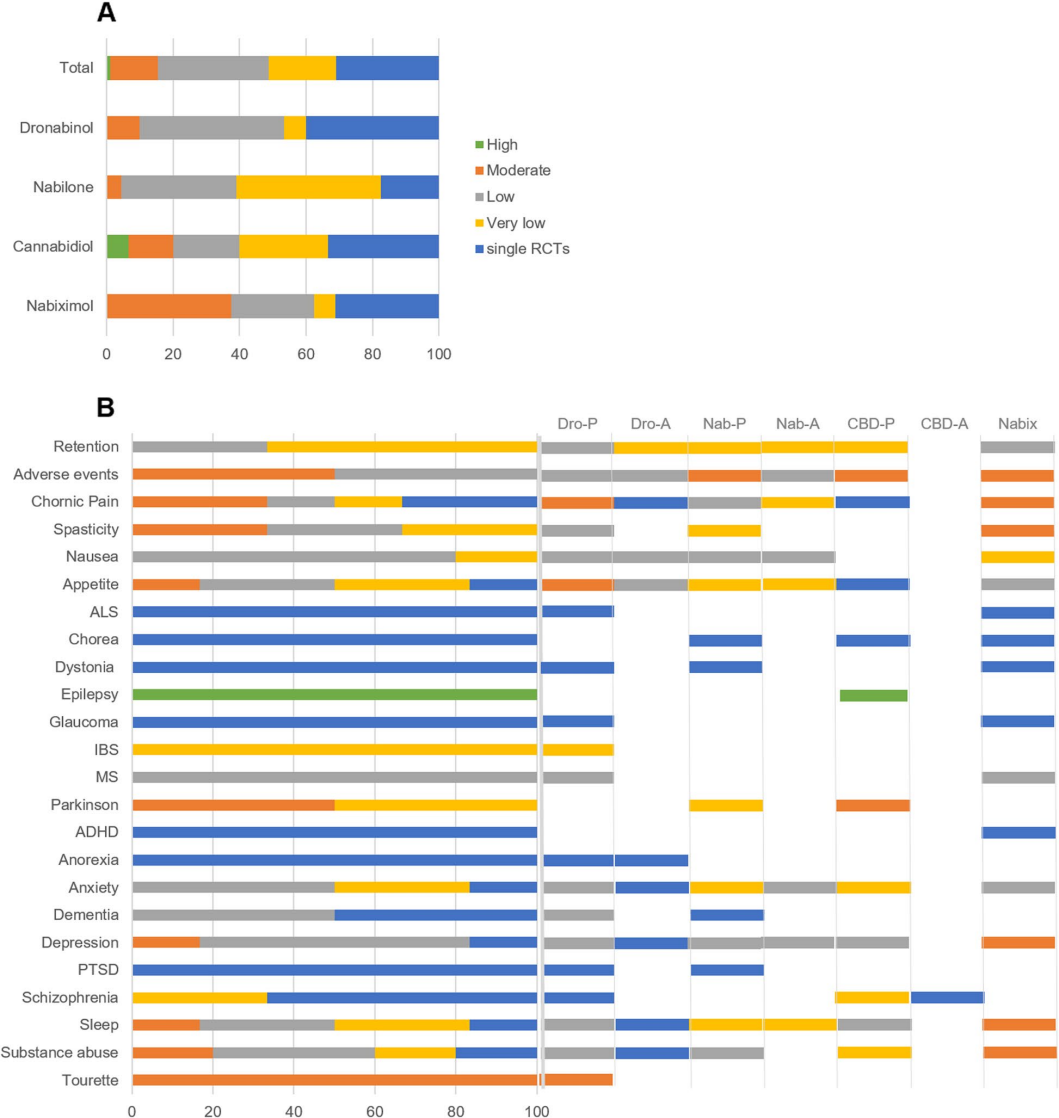
GRADE summary graph. Percentage of studies showing high, moderate, low, very low evidence and single RCTs for each cannabinoid type (**A**) and outcome (**B**)

Our 32 searches identified 6308 abstracts. Figure 1 shows a flow diagram depicting our selection procedure for the SR and meta-analysis resulting in 53 (dronabinol), 35 (nabilone), 27 (CBD) and 37 (nabiximols) selected RCTs (see Additional file 2). The list of indications by cannabinoid and characteristics of the studies are shown in Tables 1 and 2 and the full description is presented in Additional file 2: Tables S2-5 [10–160]. The summary of findings from the 152 RCTs analysed resulting in 84 comparisons (23 outcomes, 12,123 participants) is shown in Table 3 and the GRADE summary in Fig. 2. Low risk of bias was judged in 26, 6, 26 and 19% and high risk of bias was found in 5, 9, 1 and 2 studies of the dronabinol, nabilone, CBD and nabiximols trials, respectively (Additional file 3: Figs. S1-8, for references see Tables S2-5). The complete risk of bias assessment for each RCT can be found in Additional file 3: Table S6 (for references see Tables S2-5).

**Table 1:** List of indications by cannabinoid

Indication	Dronabinol	Nabilone	CBD	Nabiximols
Chronic pain	x	x	x	x
Spasticity	x	x		x
Nausea/appetite	x	x	x	x
Amyotrophic lateral sclerosis	x			x
Chorea Huntington		x	x	x
Dystonia	x	x		x
Epilepsy			x	
Glaucoma	x			x
Irritable bowel disease	x			
Multiple sclerosis	x			x
Parkinson		x	x	
ADHD				x
Anorexia nervosa	x			
Anxiety	x	x	x	x
Dementia	x	x		
Depression	x	x	x	x
PTSD	x	x		
Schizophrenia/psychosis	x		x	
Sleep	x	x	x	x
Substance abuse	x	x	x	x
Tourette	x			
Retention	x	x	x	x
Adverse events	x	x	x	x

**Table 2:** Characteristics of included studies

Intervention	Indication	No. of studies	Parallel/crossover	Maximum dose	Comparator	Treatment length (weeks)
Dronabinol	Chronic pain	16	9/7	8–129.6 mg. Most common: 2.5–15 mg (*n* = 11)	Placebo	Acute to 156. Most common: acute (*n* = 5)
		1	0/1	20 mg	Active	8
	Spasticity	6	4/2	2.5–60 mg	Placebo	< 1 week to 144. Most common: 4 (*n* = 2)
	Nausea, vomiting, loss of appetite	14	6/8	5–129.6 mg. Most common: 5–20 mg (*n* = 12)	Placebo	Acute to 6. Most common: 1–5 days (*n* = 8)
		6	3/3	5–100 mg. Most common: 5–10 (*n* = 3)	Active	Acute to 9.8. Most common: acute (*n* = 4)
	ALS	1	0/1	10 mg	Placebo	2
	Dystonia	1	0/1	15 mg	Placebo	8
	Glaucoma	1	0/1	5 mg	Placebo	Acute
	IBS	2	1/1	2.5–10 mg	Placebo	Acute
	MS	3	3/0	25–8 mg	Placebo	14 (*n* = 2), 156 (*n* = 1)
	Anorexia Nervosa	1	0/1	5 mg	Placebo	4
		1	0/1	30 mg	Active	2
	Anxiety	8	3/5	5–30 mg. Most common: 10 mg (*n* = 6)	Placebo	Acute to 7.3. Most common: acute (*n* = 6)
		2	1/1	30–50 mg	Active	Acute to 6
	Depression	8	4/4	5–30 mg. Most common: 5–10 mg (*n* = 7)	Placebo	< 1 week to 156. Most common: less than 1 week (*n* = 4)
		2	1/1	30–50 mg	Active	Acute and 6
	Dementia	3	1/2	1.5–5 mg	Placebo	1 to 6
	PTSD	1	1/0	7.5 mg	Placebo	Acute
	Psychosis/schizophrenia	1	0/1	2.5–5 mg	Placebo	Acute
	Sleep	8	4/4	2.5–129.6 mg. Most common: 10 mg (*n* = 3), 129.6 mg (*n* = 2)	Placebo	< 1 week to 14. Most common: less than 1 week to 2 (*n* = 5)
		2	1/1	30 mg	Active	2 and 6
	SUDs	4	1/3	5–40 mg	Placebo	Acute (*n* = 2), < 1 week and 8
		1	1/0	30 mg	Active	6
	Tourette	2	1/1	5–10 mg	Placebo	Acute and 6
Nabilone	Chronic pain	9	6/3	0.25–4 mg. Most common: 2 mg (*n* = 5)	Placebo	4 to 9. Most common: 4 (*n* = 4)
		3	0/3	0.5–2 mg	Active	2 to 8
	Spasticity	2	0/2	1 mg	Placebo	4
	Nausea, vomiting, loss of appetite	6	3/3	0.5–6 mg. Most common: 0.5–2 mg (*n* = 4)	Placebo	Acute/ < 1 week (*n* = 3) to 6–8 (*n* = 3)
		11	3/8	1–8 mg. Most common: 1–4 mg (*n* = 10)	Active	Acute to 1
	Huntington	1	0/1	1 and 2 mg	Placebo	5
	Dystonia	1	0/1	0.03 mg	Placebo	Acute
	Parkinson	2	1/1	0.06 and 2 mg	Placebo	Acute and 4
	Anxiety	8	5/3	1–8 mg	Placebo	< 1 week to 10
		2	0/2	0.5 and 2 mg	Active	6 and 8
	Dementia	1	0/1	2 mg	Placebo	6
	Depression	5	4/1	1–5 mg	Placebo	Acute to 10. Most common: 4–5 (*n* = 3)
		2	0/2	0.5 and 2 mg	Active	6 and 8
	PTSD	1	0/1	3 mg	Placebo	7
	Sleep	7	4/3	1–8 mg. Most common: 1–4 mg (*n* = 6)	Placebo	< 1 week to 8
		2	0/2	0.5, 1 and 2 mg	Active	2 and 6
	SUDs	3	1/2	2, 6 and 8 mg	Placebo	< 1 week (*n* = 2) and 10
Cannabidiol	Chronic pain	1	1/0	20 mg, 30 mg	Placebo	12
	Nausea, vomiting, loss of appetite	1	1/0	200 mg	Placebo	13
	Huntington	1	0/1	10 mg	Placebo	6
	Epilepsy	6	6/0	10–300 mg. Most common: 10–20 mg (*n* = 4)	Placebo	14 (*n* = 4), 16 and 18
	Parkinson	3	2/1	75 and 300 mg	Placebo	Acute, 6 and 12
	Anxiety	11	9/2	400–800 mg. Most common: 300 mg (*n* = 4), 400 mg (*n* = 3)	Placebo	Acute to 12. Most common: acute to < 1 week (*n* = 5)
	Depression	6	6/0	75–400 mg	Placebo	1 to 13
	Psychosis/schizophrenia	4	3/1	300 mg, 600 mg (*n* = 2) and 1000 mg	Placebo	Acute (*n* = 2) and 6 (*n* = 2)
		1	1/0	800 mg	Active	4
	Sleep	9	9/0	10–1000 mg. Most common: 10–20 mg (*n* = 5)	Placebo	1 to 14. Most common: 12–14 (*n* = 5)
	SUDs	7	6/2	400–800 mg. Most common doses: 800 mg (*n* = 5)	Placebo	Acute to 13. Most common: < 1 week (*n* = 4)
Nabiximols	Chronic pain	20	16/4	10.8–129.6 mg THC: 10–120 mg CBD. Most common: 32.4 mg THC: 30 mg:CBD (*n* = 7), 129.6 mg THC: 120 mg CBD (*n* = 5)	Placebo	2 to 14. Most common: 5 (*n* = 6), 12 (*n* = 4)
	Spasticity	14	6/4	8.1–129.6 mg THC: 7.5–120 mg CBD. Most common: 32.4 mg THC: 30 mg:CBD (*n* = 6), 129.6 mg THC: 120 mg CBD (*n* = 2)	Placebo	2 to 14. Most common: 12 (*n* = 3), 6 (*n* = 3), 4 (*n* = 3)
	Nausea, vomiting, loss of appetite	8	6/2	5–129.6 mg THC: 2–120 mg CBD	Placebo	< 1 week to 12. Most common: < 1 week (*n* = 3)
	ALS	1	1/0	32.4 mg THC: 30 mg CBD	Placebo	6
	Chorea Hunington	1	0/1	32.4 mg THC: 30 mg CBD	Placebo	12
	Dystonia	1	0/1	32.4 mg THC: 30 mg CBD	Placebo	12
	Glaucoma	1	0/1	CBD: THC (1:21). 20, 40 mg	Placebo	Acute
	MS	5	4/1	30–129.6 mg THC: 10.8–120 mg CBD	Placebo	2 to 14. Most common: 14 (*n* = 2)
	ADHD	1	1/0	37.8 mg THC: 35 mg CBD	Placebo	6
	Depression	8	5/3	32.4–129.6 mg THC: 30–120 mg CBD. Most common: 32.4 mg THC: 30 mg:CBD (*n* = 2)	Placebo	< 1 week to 12. Most common: 12 (*n* = 3)
	Anxiety	6	3/3	30–129.6 mg THC: 10.8–120 mg CBD	Placebo	3 to 12. Most common: 12 (*n* = 2)
	Sleep	22	18/4	16.2–129.6 mg THC: 15–120 mg CBD. Most common: 32.4 mg THC: 30 mg:CBD (*n* = 6), 129.6 mg THC: 120 mg CBD (*n* = 4)	Placebo	< 1 week to 14. Most common: 5 (*n* = 6), 12 (*n* = 4)
	SUDs	4	3/1	64.8–113.4 mg THC: 60–105 mg CBD	Placebo	< 1 week to 12. Most common: 12 (*n* = 2)

**Table 3:** Summary of findings. GRADE Working Group grades of evidence. High quality: Further research is very unlikely to change our confidence in the estimate of effect. Moderate quality: Further research is likely to have an important impact on our confidence in the estimate of effect and may change the estimate. Low quality: Further research is very likely to have an important impact on our confidence in the estimate of effect and is likely to change the estimate. Very low quality: We are very uncertain about the estimate

	**Comparison**	**No. of participants (studies)**	***I***^**2**^** (%)**	**GRADE**	**Relative effect (95% CI)**	**Anticipated absolute effects**	
**Outcome**						**Risk with control**	**Risk difference with Cannabinoids**
Chronic pain	Dronabinol vs Placebo	1395 (15 RCTs)	53	⨁⨁⨁◯ MODERATE ^c^			SMD **0.31 lower** (0.46 lower to 0.15 lower)
	Dronabinol vs Active	14 (1 RCT)					SMD **0.73 higher** (0.1 lower to 1.56 higher)
	Nabilone vs Placebo	363 (9 RCTs)	70	⨁⨁◯◯ LOW ^c,g^			SMD **0.41 lower** (0.76 lower to 0.06 lower)
	Nabilone vs Active	262 (3 RCTs)	83	⨁◯◯◯ VERY LOW ^c,e^			SMD **0.08 lower** (0.54 lower to 0.38 higher)
	Cannabidiol vs Placebo	129 (1 RCT)					SMD **0.01 higher** (0.34 lower to 0.35 higher)
	Nabiximols vs Placebo	3238 (21 RCTs)	65	⨁⨁⨁◯ MODERATE ^c^			SMD **0.23 lower** (0.34 lower to 0.12 lower)
Spasticity	Dronabinol vs Placebo	704 (6 RCTs)	52	⨁⨁◯◯ LOW ^c,f^			SMD **0.08 lower** (0.34 lower to 0.17 higher)
	Nabilone vs Placebo	44 (2 RCTs)	68	⨁◯◯◯ VERY LOW ^a,c,g^			SMD **0.7 lower** (1.56 lower to 0.16 higher)
	Nabiximols vs Placebo	1658 (14 RCTs)	68	⨁⨁⨁◯ MODERATE ^c^			SMD **0.36 lower** (0.54 lower to 0.19 lower)
Nausea/vomiting	Dronabinol vs Placebo	495 (8 RCTs)	61	⨁⨁◯◯ LOW ^c,f^			SMD **0 .21 lower** (0.49 lower to 0.07 higher)
	Dronabinol vs Active	308 (5 RCTs)	0	⨁⨁◯◯ LOW ^a,g^			SMD **0.28 lower** (0.47 lower to 0.1 lower)
	Nabilone vs Placebo	603 (4 RCTs)	61	⨁⨁◯◯ LOW ^c,f^			SMD **0.09 lower** (0.36 lower to 0.18 higher)
	Nabilone vs Active	627 (11 RCTs)	46	⨁⨁◯◯ LOW ^b,c^			SMD **0.44 lower** (0.62 lower to 0.26 lower)
	Nabiximols vs Placebo	393 (6 RCTs)	56	⨁◯◯◯ VERY LOW ^a,e^			SMD **0.23 lower** (0.55 lower to 0.08 higher)
Appetite	Dronabinol vs Placebo	599 (10 RCTs)	81	⨁⨁⨁◯ MODERATE ^c^			SMD **0.51 lower** (0.87 lower to 0.15 lower)
	Dronabinol vs Active	795 (3 RCTs)	86	⨁⨁◯◯ LOW ^c,f^			SMD **0.02 lower** (0.39 lower to 0.34 higher)
	Nabilone vs Placebo	187 (4 RCTs)	74	⨁◯◯◯ VERY LOW ^c,e^			SMD **0.44 lower** (0.98 lower to 0.11 higher)
	Nabilone vs Active	130 (3 RCTs)	85	⨁◯◯◯ VERY LOW ^a,d, g^			SMD **0.24 higher** (0.51 lower to 0.99 higher)
	Cannabidiol vs Placebo	27 (1 RCT)					SMD **0.1 higher** (0.66 lower to 0.85 higher)
	Nabiximols vs Placebo	430 (6 RCTs)	66	⨁⨁◯◯ LOW ^c,f^			SMD **0.25 lower** (0.61 lower to 0.1 higher)
Amyotrophic lateral sclerosis	Dronabinol vs Placebo	22 (1 RCT)					SMD **0.26 higher** (0.17 lower to 0.68 higher)
	Nabiximols vs Placebo	59 (1 RCT)					SMD **0.38 higher** (0.13 lower to 0.90 higher)
Chorea Huntington	Nabilone vs Placebo	74 (1 RCT)					SMD **0.45 lower** (0.79 lower to 0.11 lower)
	Cannabidiol vs Placebo	30 (1 RCT)					SMD **0.18 higher** (0.33 lower to 0.69 higher)
	Nabiximols vs Placebo	50 (1 RCT)					SMD **0.17 higher** (0.23 lower to 0.56 higher)
Dystonia	Dronabinol vs Placebo	14 (1 RCT)					SMD **0.05 higher** (0.69 lower to 0.79 higher)
	Nabilone vs Placebo	26 (1 RCT)					SMD **0.49 lower** (1.07 lower to 0.08 higher)
	Nabiximols vs Placebo	50 (1 RCT)					SMD **0** (0.39 lower to 0.39 higher)
Epilepsy	Cannabidiol vs Placebo	956 (6 RCTs)	0	⨁⨁⨁⨁ HIGH			SMD **0.5 lower** (0.62 lower to 0.38 lower)
Glaucoma	Dronabinol vs Placebo	12 (1 RCT)					SMD **1.28 lower** (2.36 lower to 0.2 lower)
	Nabiximols vs Placebo	12 (1 RCT)					SMD **0.82 higher** (0.1 lower to 1.75 higher)
IBS	Dronabinol vs Placebo	81 (2 RCTs)	85	⨁◯◯◯ VERY LOW ^c,e^			SMD **0** (1.09 lower to 1.09 higher)
MS	Dronabinol vs Placebo	660 (2 RCTs)	74	⨁⨁◯◯ LOW ^c,f^			SMD **0.15 lower** (0.51 lower to 0.22 higher)
	Nabiximols vs Placebo	863 (5 RCTs)	65	⨁⨁◯◯ LOW ^c,f^			SMD **0.14 lower** (0.38 lower to 0.11 higher)
Parkinson	Nabilone vs Placebo	51 (2 RCTs)	46	⨁◯◯◯ VERY LOW ^c,e^			SMD **0.38 lower** (1.05 lower to 0.29 higher)
	Cannabidiol vs Placebo	101 (3 RCTs)	0	⨁⨁⨁◯ MODERATE ^g^			SMD **0.41 lower** (0.75 lower to 0.08 lower)
ADHD	Nabiximols vs Placebo	30 (1 RCT)					SMD **0.83 lower** (1.58 lower to 0.09 lower)
Anorexia nervosa	Dronabinol vs Placebo	48 (1 RCT)					SMD **0.47 lower** (0.89 lower to 0.05 lower)
	Dronabinol vs Active	22 (1 RCT)					SMD **0.06 lower** (0.65 lower to 0.53 higher)
Anxiety	Dronabinol vs Placebo	113 (4 RCTs)	49	⨁⨁◯◯ LOW ^e^			SMD **0.03 lower** (0.47 lower to 0.41 higher)
	Dronabinol vs Active	278 (1 RCT)					SMD **0.14 higher** (0.03 lower to 0.31 higher)
	Nabilone vs Placebo	147 (6 RCTs)	84	⨁◯◯◯ VERY LOW ^a,c,e^			SMD **0.59 lower** (1.4 lower to 0.22 higher)
	Nabilone vs Active	192 (2 RCTs)	11	⨁⨁◯◯ LOW ^e^			SMD **0.11 lower** (0.33 lower to 0.11 higher)
	Cannabidiol vs Placebo	481 (11 RCTs)	78	⨁◯◯◯ VERY LOW ^c,e^			SMD **0.34 lower** (0.73 lower to 0.06 higher)
	Nabiximols vs Placebo	258 (6 RCTs)	43	⨁⨁◯◯ LOW ^e^			SMD **0.06 higher** (0.23 lower to 0.34 higher)
Dementia	Dronabinol vs Placebo	115 (3 RCTs)	3	⨁⨁◯◯ LOW ^e^			SMD **0.27 lower** (0.57 lower to 0.04 higher)
	Nabilone vs Placebo	76 (1 RCT)					SMD **0.53 lower** (0.87 lower to 0.19 lower)
Depression	Dronabinol vs Placebo	700 (7 RCTs)	83	⨁⨁◯◯ LOW ^c,f^			SMD **0.15 lower** (0.49 lower to 0.19 higher)
	Dronabinol vs Active	278 (1 RCT)					SMD **0.14 higher** (0.03 lower to 0.31 higher)
	Nabilone vs Placebo	76 (3 RCTs)	0	⨁⨁◯◯ LOW ^e^			SMD **0.03 lower** (0.48 lower to 0.43 higher)
	Nabilone vs Active	192 (2 RCTs)	0	⨁⨁◯◯ LOW ^e^			SMD **0.03 lower** (0.23 lower to 0.17 higher)
	Cannabidiol vs Placebo	301 (6 RCTs)	0	⨁⨁◯◯ LOW ^e^			SMD **0.12 higher** (0.09 lower to 0.34 higher)
	Nabiximols vs Placebo	413 (7 RCTs)	43	⨁⨁⨁◯ MODERATE ^f^			SMD **0.12 lower** (0.36 lower to 0.13 higher)
PTSD	Dronabinol vs Placebo	46 (1 RCT)					SMD **0.63 lower** (1.22 lower to 0.03 lower)
	Nabilone vs Placebo	18 (1 RCT)					SMD **0.88 lower** (1.65 lower to 0.11 lower)
Schizophrenia /psychosis	Dronabinol vs Placebo	26 (1 RCT)					SMD **0.89 higher** (0.25 higher to 1.53 higher)
	Cannabidiol vs Placebo	152 (3 RCTs)	70	⨁◯◯◯ VERY LOW ^c,f^			SMD **0.24 lower** (0.81 lower to 0.33 higher)
	Cannabidiol vs Active	39 (1 RCT)					SMD **0.05 higher** (0.58 lower to 0.68 higher)
Sleep	Dronabinol vs Placebo	683 (7 RCTs)	64	⨁⨁◯◯ LOW ^c,f^			SMD **0.13 lower** (0.39 lower to 0.12 higher)
	Dronabinol vs Active	22 (1 RCT)					SMD **1.12 higher** (0.37 higher to 1.87 higher)
	Nabilone vs Placebo	175 (6 RCTs)	77	⨁◯◯◯ VERY LOW ^c,g^			SMD **0.58 lower** (1.14 lower to 0.01 lower)
	Nabilone vs Active	200 (2 RCTs)	91	⨁◯◯◯ VERY LOW ^d,e^			SMD **0.21 lower** (0.97 lower to 0.55 higher)
	Cannabidiol vs Placebo	961 (8 RCTs)	46	⨁⨁◯◯ LOW ^a,c^			SMD **0.06 lower** (0.23 lower to 0.11 higher)
	Nabiximols vs Placebo	3659 (23 RCTs)	61	⨁⨁⨁◯ MODERATE ^c^			SMD **0.24 lower** (0.35 lower to 0.14 lower)
SUDs	Dronabinol vs Placebo	196 (3 RCTs)	0	⨁⨁◯◯ LOW ^a,g^			SMD **0.47 lower** (0.73 lower to 0.2 lower)
	Dronabinol vs Active	60 (1 RCT)					SMD **0.85 lower** (1.41 lower to 0.29 lower)
	Nabilone vs Placebo	70 (3 RCTs)	0	⨁⨁◯◯ LOW ^a,g^			SMD **0.55 lower** (0.93 lower to 0.18 lower)
	Cannabidiol vs Placebo	353 (7 RCTs)	81	⨁◯◯◯ VERY LOW ^c,e^			SMD **0.2 lower** (0.63 lower to 0.24 higher)
	Nabiximols vs Placebo	237 (4 RCTs)	59	⨁⨁⨁◯ MODERATE ^g^			SMD **0.48 lower** (0.92 lower to 0.04 lower)
Tourette	Dronabinol vs Placebo	41 (2 RCTs)	0	⨁⨁⨁◯ MODERATE ^g^			SMD **1.01 lower** (1.58 lower to 0.44 lower)
Retention	Dronabinol vs Placebo	3285 (37 RCTs)	65	⨁⨁◯◯ LOW ^c,f^	**OR 1.11** (0.80 to 1.53)	194 per 1.000	**17 more per 1.000** (33 fewer to 75 more)
	Dronabinol vs Active	1079 (8 RCTs)	32	⨁◯◯◯ VERY LOW ^a,e^	**OR 1.03** (0.76 to 1.40)	422 per 1.000	**7 more per 1.000** (65 fewer to 83 more)
	Nabilone vs Placebo	1070 (16 RCTs)	0	⨁◯◯◯ VERY LOW ^a,e^	**OR 0.99** (0.76 to 1.29)	143 per 1.000	**1 fewer per 1.000** (30 fewer to 34 more)
	Nabilone vs Active	1004 (13 RCTs)	0	⨁◯◯◯ VERY LOW ^a,e^	**OR 0.99** (0.79 to 1.25)	205 per 1.000	**2 fewer per 1.000** (36 fewer to 39 more)
	Cannabidiol vs Placebo	1775 (22 RCTs)	41	⨁◯◯◯ VERY LOW ^c,g^	**OR 1.38** (0.77 to 2.47)	71 per 1.000	**24 more per 1.000** (15 fewer to 88 more)
	Nabiximols vs Placebo	4643 (33 RCTs)	44	⨁⨁◯◯ LOW ^c,f^	**OR 1.17** (0.92 to 1.49)	170 per 1.000	**23 more per 1.000** (11 fewer to 64 more)
Adverse events	Dronabinol vs Placebo	2707 (37 RCTs)	56	⨁⨁◯◯ LOW ^c^	**OR 2.16** (1.59 to 2.94)	320 per 1.000	**184 more per 1.000** (108 more to 260 more)
	Dronabinol vs Active	925 (8 RCTs)	67	⨁⨁◯◯ LOW ^a,c^	**OR 2.75** (1.43 to 5.26)	466 per 1.000	**240 more per 1.000** (89 more to 355 more)
	Nabilone vs Placebo	996 (16 RCTs)	76	⨁⨁⨁◯ MODERATE ^c^	**OR 3.12** (1.52 to 6.42)	226 per 1.000	**251 more per 1.000** (81 more to 426 more)
	Nabilone vs Active	909 (13 RCTs)	71	⨁⨁◯◯ LOW ^a,c^	**OR 2.47** (1.45 to 4.20)	223 per 1.000	**192 more per 1.000** (71 more to 324 more)
	Cannabidiol vs Placebo	1736 (22 RCTs)	58	⨁⨁⨁◯ MODERATE ^c^	**OR 1.82** (1.08 to 3.07)	482 per 1.000	**147 more per 1.000** (19 more to 259 more)
	Nabiximols vs Placebo	4404 (33 RCTs)	74	⨁⨁⨁◯ MODERATE ^c^	**OR 1.97** (1.48 to 2.64)	536 per 1.000	**159 more per 1.000** (95 more to 217 more)

*The basis for the assumed risk (e.g. the median control group risk across studies) is provided in footnotes. The risk difference (and its 95% confidence interval) is based on the assumed risk in the comparison group and the relative effect of the intervention (and its 95% CI). *CI* confidence interval, *OR* odds ratio. ^a^Moderate-High risk of bias; ^b^Many high risk of bias; ^c^Moderate-Substantial heterogeneity; ^d^High heterogeneity; ^e^Optimal information size not met, CI overlaps no effect; ^f^Optimal information size met, CI overlaps no effect; ^g^Optimal information size not met, CI excludes no effect

### Primary outcomes

#### Chronic pain

**Fig. 3:**
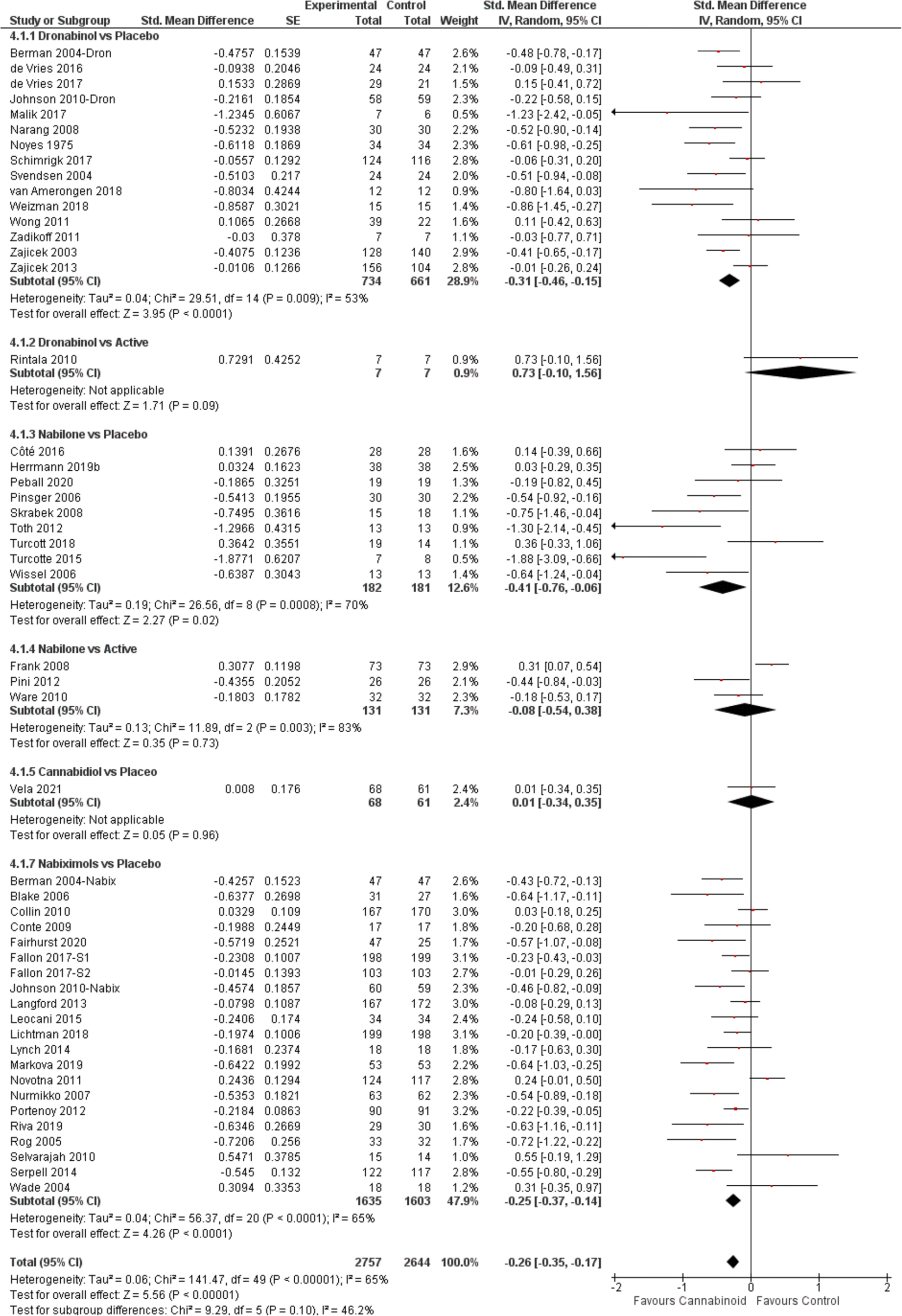
Chronic pain forest plot, stratified according to cannabinoid type and comparator used. The horizontal lines indicate 95% CIs. The diamond markers represent the subtotal and overall weighed standardized mean difference (SMD) mean difference and 95% CI. The vertical line shows the line of no effect

The meta-analysis (Fig. 3) showed the beneficial effect of cannabinoids on chronic pain (SMD − 0.26, 95% CI − 0.35 to − 0.17; *P* < 0.00001). Further subgroup analyses indicated that compared to placebo, dronabinol [10, 11, 95, 106, 139, 150, 161, 12, 23, 34, 45, 56, 72, 73, 84] and nabiximols [10, 33–44, 46–52] were associated with significant improvements and moderate evidence (Fig. 2B) in conditions causing chronic pain (dronabinol SMD − 0.31; nabiximols SMD − 0.25, *P* < 0.0001). Trials using nabilone vs placebo [114, 115, 118, 119, 122, 124–126, 162] (but not vs active [120, 121, 123]) also reported a significant effect (SMD − 0.41, *P* = 0.02), but the evidence on this effect was low (Fig. 2B). The to date single RCTs with CBD vs placebo [153] and dronabinol vs active drug [69] reported no effect.

#### Spasticity with MS and paraplegia

**Fig. 4:**
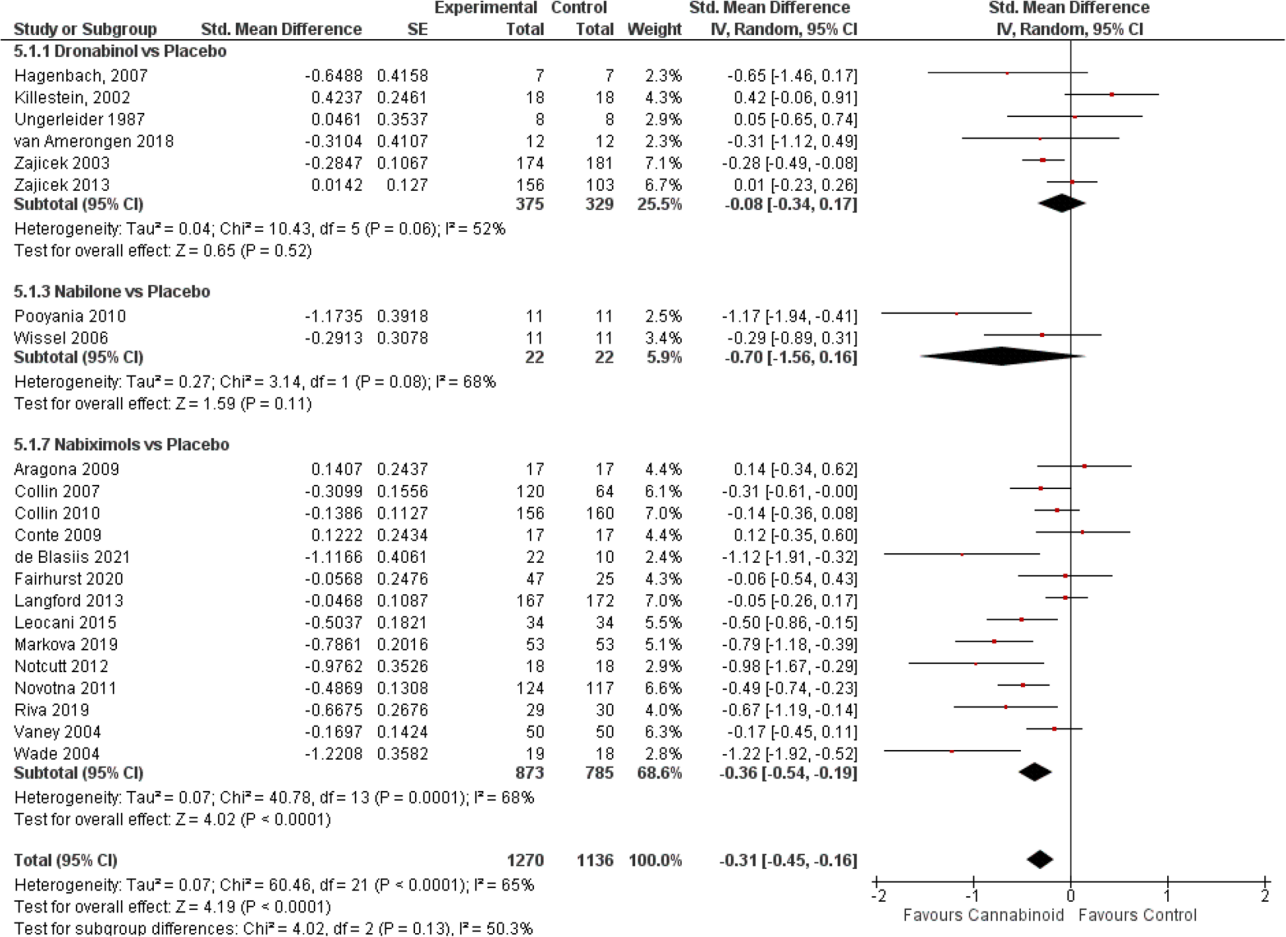
Spasticity forest plot, stratified according to cannabinoid type and comparator used. The horizontal lines indicate 95% CIs. The diamond markers represent the subtotal and overall weighed standardized mean difference (SMD) mean difference and 95% CI. The vertical line shows the line of no effect

When all RCTs were pooled (Fig. 4), a significant effect favouring cannabinoids was found (SMD − 0.31, 95% CI − 0.45 to − 0.16; *P* < 0.0001). Yet, subgroup analyses indicated that only nabiximols [38, 40–44, 46, 47, 49, 53–55, 57, 58] were associated with improvements in spasticity (SMD − 0.36, 95% CI − 0.54 to − 0.19; *P* < 0.0001), and the limited number of studies found with dronabinol [12, 67, 70–72, 150] /nabilone [126, 127] did not provide enough evidence.

#### Nausea and vomiting

The meta-analysis of nausea and vomiting (Additional file 4: Fig. S9) including all studies showed a general efficacy of cannabinoids (SMD − 0.29, 95% CI − 0.39 to − 0.18; *P* < 0.00001). Confidence on the results from earlier trials reporting improvements in nausea and vomiting versus an active comparator (dronabinol [77, 80, 83, 87]: SMD − 0.28, *P* = 0.003; nabilone [129–138, 141]: SMD − 0.44, *P* < 0.00001) is low due to the lack of methodical rigor. Dronabinol [10, 76, 79, 81, 82, 85, 88, 91], nabilone [115, 140, 142, 162] and nabiximols [10, 59, 60, 62, 63, 85]) were not better than placebo.

#### Appetite

**Fig. 5:**
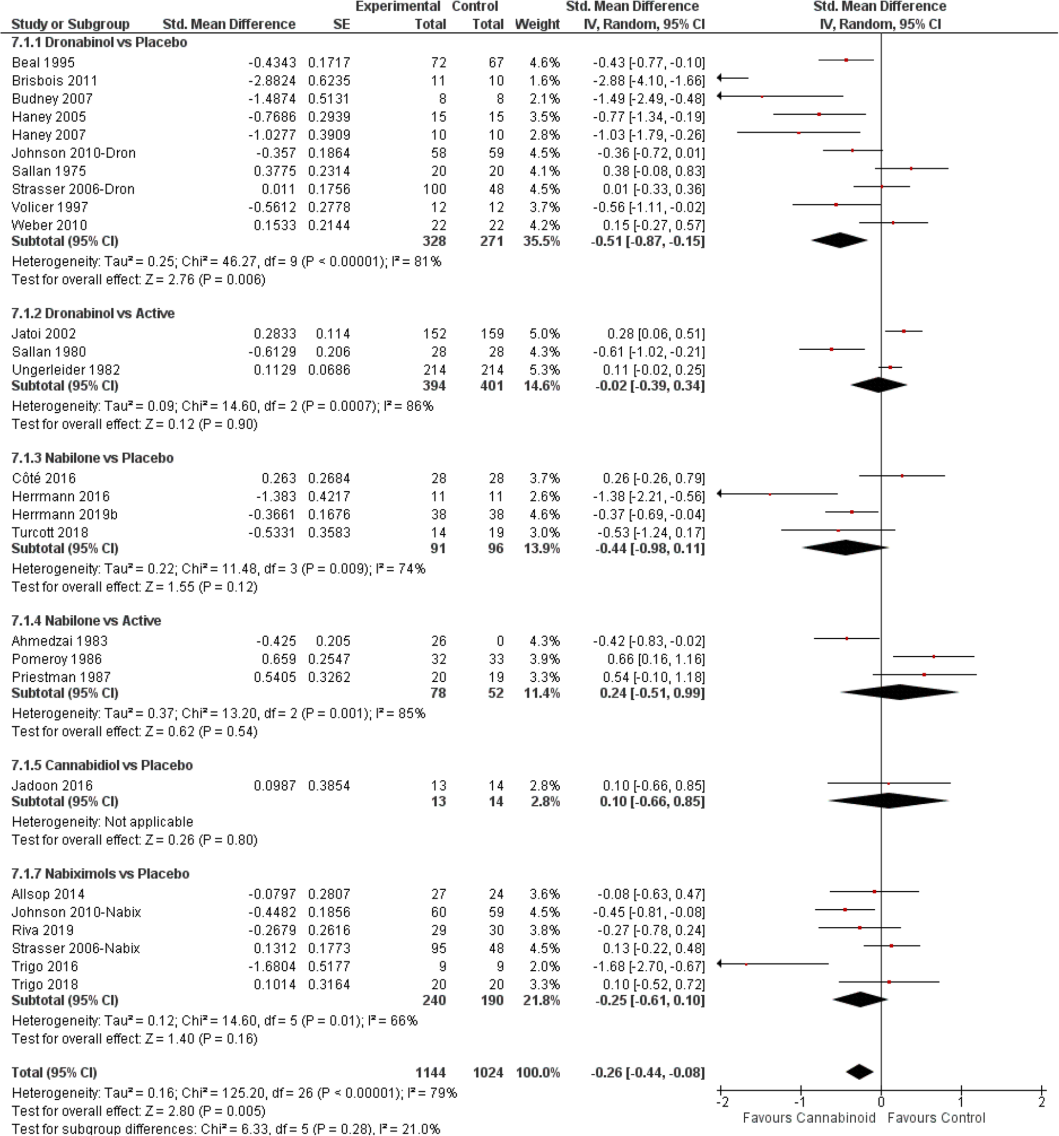
Appetite forest plot, stratified according to cannabinoid type and comparator used. The horizontal lines indicate 95% CIs. The diamond markers represent the subtotal and overall weighed standardized mean difference (SMD) mean difference and 95% CI. The vertical line shows the line of no effect

The meta-analysis (Fig. 5) showed the efficacy of cannabinoids for increasing appetite scores compared to the control arms (SMD − 0.26, *P* = 0.005). Only the combination of dronabinol-placebo [10, 74–76, 82, 85, 88–90, 92] (but not vs active [78, 83, 86]) retained the stimulating effect on appetite (SMD − 0.51, 95% CI − 0.87 to − 0.15; *P* = 0.006). Low/very low evidence and a lack of significance was found for nabilone (vs placebo [114, 115, 143, 162]: SMD − 044, *P* = 0.12; vs active [129, 137, 138]: SMD 0.24), CBD [154] (SMD 0.10, *P* = 0.80) or nabiximols [10, 40, 61–63, 85] (SMD − 0.25, *P* = 0.16).

#### Amyotrophic lateral sclerosis

To date, only one cross-over RCT with dronabinol [75] and one parallel RCT with nabiximols [40] have been carried out in patients suffering from ALS (Additional file 4: Fig. S10). The two trials did not report any improvement in ALS scores and the pooled effect indicated an almost significant effect favouring placebo (SMD 0.31, *P* = 0.07).

#### Chorea Huntington

The meta-analysis of the three included studies (Additional file 4: Fig. S11) showed a tendency towards favouring cannabinoids with significant subgroup differences (*P* = 0.03). That is, the calculated SMD from a single study with nabilone [144] (SMD − 0.45, 95% CI − 0.79 to − 0.11; *P* = 0.009) but not with CBD [155] or nabiximols [64] (SMD 0.18, *P* = 0.48 / SMD 0.17, *P* = 0.4) was significant.

#### Dystonia

Results with the three small cross-over studies (Additional file 4: Fig. S12) showed a potential benefit of nabilone [145] (SMD − 0.49, *P* = 0.09) and a lack of effect of nabiximols [64] (SMD 0) and dronabinol [73] (SMD 0.05).

#### Epilepsy

**Fig. 6:**
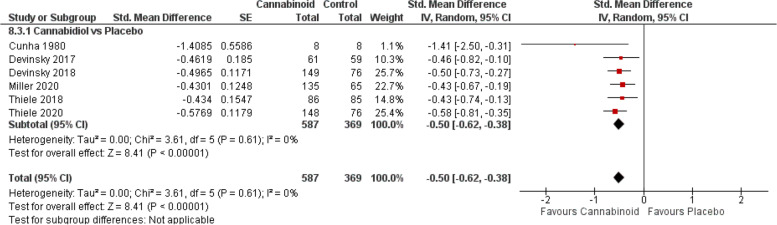
Epilepsy forest plot. The horizontal lines indicate 95% CIs. The diamond markers represent the subtotal and overall weighed standardized mean difference (SMD) mean difference and 95% CI. The vertical line shows the line of no effect

First reported by an early small study [156] and recently by a series of publications from a large international clinical trial [13, 157–160], CBD was associated with a significant decrease in seizure frequencies (Fig. 6, SMD − 0.50, 95% CI − 0.62 to 0.38; *P* < 0.00001).

#### Glaucoma

Only a very small cross-over trial [94] tested the effects of dronabinol and nabiximols on ocular hypertension (Additional file 4: Fig. S13). Dronabinol produced a transient benefit (SMD − 1.28, 95% CI − 2.36 to − 0.20; *P* = 0.02), while nabiximols (CBD combined with small amounts of dronabinol) resulted in a transient worsening (SMD − 0.82, *P* = 0.08).

#### Irritable bowel syndrome

Two (one parallel [106] and one cross-over [96]) studies tested the effect of acute dronabinol administration on colonic and visceral symptoms (Additional file 4: Fig. S14). Individual results favoured dronabinol and placebo, respectively, resulting in an overall no effect (SMD 0) with a very low evidence.

#### Multiple sclerosis

Nabiximols [41, 43, 49, 58, 65] or/and dronabinol [97, 117] did not improve symptoms associated with MS (Additional file 4: Fig. S15, overall SMD − 0.13, 95% CI − 0.31 to 0.05; *P* = 0.15), and none of the subgroups achieved significant improvements (dronabinol SMD − 0.15, *P* = 0.43; nabiximols SMD − 0.14, *P* = 0.28).

#### Parkinson’s disease

**Fig. 7:**
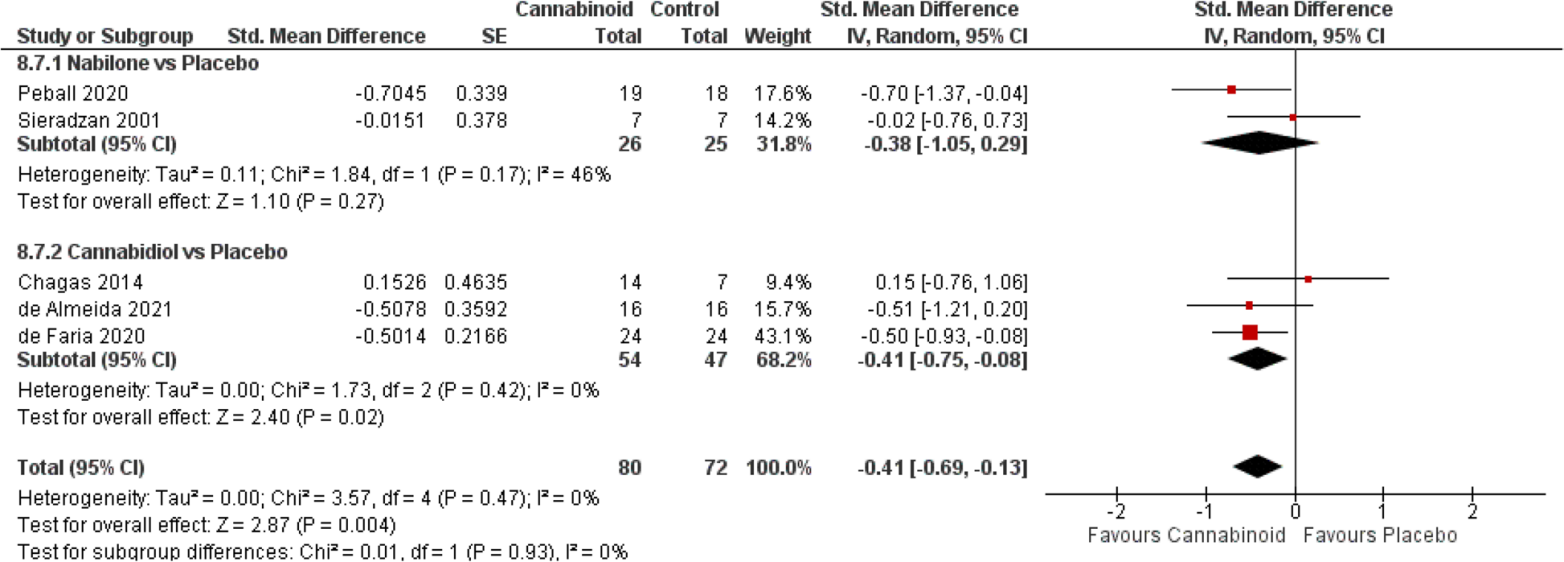
Parkinson’ disease forest plot, stratified according to cannabinoid type and comparator used. The horizontal lines indicate 95% CIs. The diamond markers represent the subtotal and overall weighed standardized mean difference (SMD) mean difference and 95% CI. The vertical line shows the line of no effect

Meta-analysis from all studies (Fig. 7) favoured cannabinoids (SMD − 0.41, 95% CI − 0.69 to − 0.13; *P* = 0.004), and subgroup analyses indicated that CBD [14–16] (SMD − 0.41, 95% CI − 0.75 to − 0.08; *P* = 0.02) but not nabilone [125, 146] (SMD − 0.38; *P* = 0.27) was associated with a significant improvement in parkinsonian symptoms.

#### ADHD

One small parallel RCT [66] comparing nabiximols with placebo in ADHD found significant differences in scores of hyperactivity and impulsivity (SMD − 0.83, 95% CI − 1.58 to − 0.09; *P* = 0.03).

##### Anorexia nervosa

Two small cross-over RCTs with dronabinol [98, 100] (Additional file 4: Fig. S16) found an increase in body weight when compared with placebo (SMD − 0.47; *P* = 0.03), but not with diazepam (SMD − 0.06, *P* = 0.84).

#### Anxiety

Measurements of anxiety were included in dronabinol vs placebo trials in 4 RCTs [23, 45, 92, 102] and vs prochlorperazine in one study [86]; nabilone in comparison with placebo trials in 6 RCTs [118, 119, 125, 143, 148, 151] and versus active comparators in two RCTs [121, 123]; in 11 RCTs [15–18, 20–22, 24, 25, 153, 163] comparing CBD to placebo and in six nabiximols trials [48, 53, 61–64]. The meta-analysis including all studies (Additional file 4: Fig. S17) showed that cannabinoids attenuate anxiety levels (SMD − 0.19, 95% CI − 0.37 to − 0.00; *P* = 0.05), but none of the subgroup analysis showed a significant improvement in anxiety. The quality of evidence of these results was low or very low (Fig. 2B).

#### Dementia

Disturbed, agitated behaviour in dementia was assessed in 4 RCTs (Additional file 4: Fig. S18), with an overall significant effect (SMD − 0.37, 95% CI − 0.61 to − 0.13; *P* = 0.002); however, the evidence for specific cannabinoids is low or missing (Fig. 2B). While the three studies with dronabinol [74, 105, 107] collectively did not reach significance (SMD − 0.27, *P* = 0.09), a single study with nabilone [114] reported a significant reduction (SMD − 0.53, 95% CI − 0.87 to − 0.19; *P* = 0.002).

#### Depression

Symptoms of depression caused by diverse medical conditions were evaluated with dronabinol in seven RCTs versus placebo [12, 23, 45, 75, 92, 102, 117] and in one study versus prochlorperazine [86]; with nabilone, three studies comparing placebo [118, 125, 151] and two comparing an active drug [121, 123] were carried out; placebo was compared with CBD in 6 RCTs [15, 19, 22, 24, 153, 154] and with nabiximols in 7 RCTs [48, 49, 53, 61–64]. The overall meta-analysis (Additional file 4: Fig. S19, SMD − 0.04, *P* = 0.53) was consistent with the results found in all subgroups reporting minor or no attenuations of depressive symptoms. CBD and nabilone did not modify depressive symptoms, and dronabinol and nabiximols showed a minor improvement compared with placebo (dronabinol: SMD − 0.15, *P* = 0.39; nabiximols: SMD − 0.12, *P* = 0.35), but the evidence was moderate only for nabiximols (Fig. 2B).

#### PTSD

Two small studies with dronabinol [104] and nabilone [152] (Additional file 4: Fig. S20) found significant improvements compared with placebo (dronabinol: SMD − 0.63, 95% CI − 1.22 to − 0.03; *P* = 0.04; nabilone: SMD − 0.88, 95% CI − 1.65 to − 0.11; *P* = 0.03).

#### Schizophrenia and psychosis

The trials evaluating PANNS symptoms (Additional file 4: Fig. S21) showed no effect of cannabinoids (SMD 0.04, *P* = 0.89) but with subgroup differences (*P* = 0.03). Thus, a study with dronabinol [108] found a deterioration (SMD 0.89, 95% CI 0.25 to 1.53; *P* = 0.007), whereas CBD [25, 26, 28, 164] had no effect but the grade of evidence was very low (Fig. 2B).

#### Sleep

**Fig. 8:**
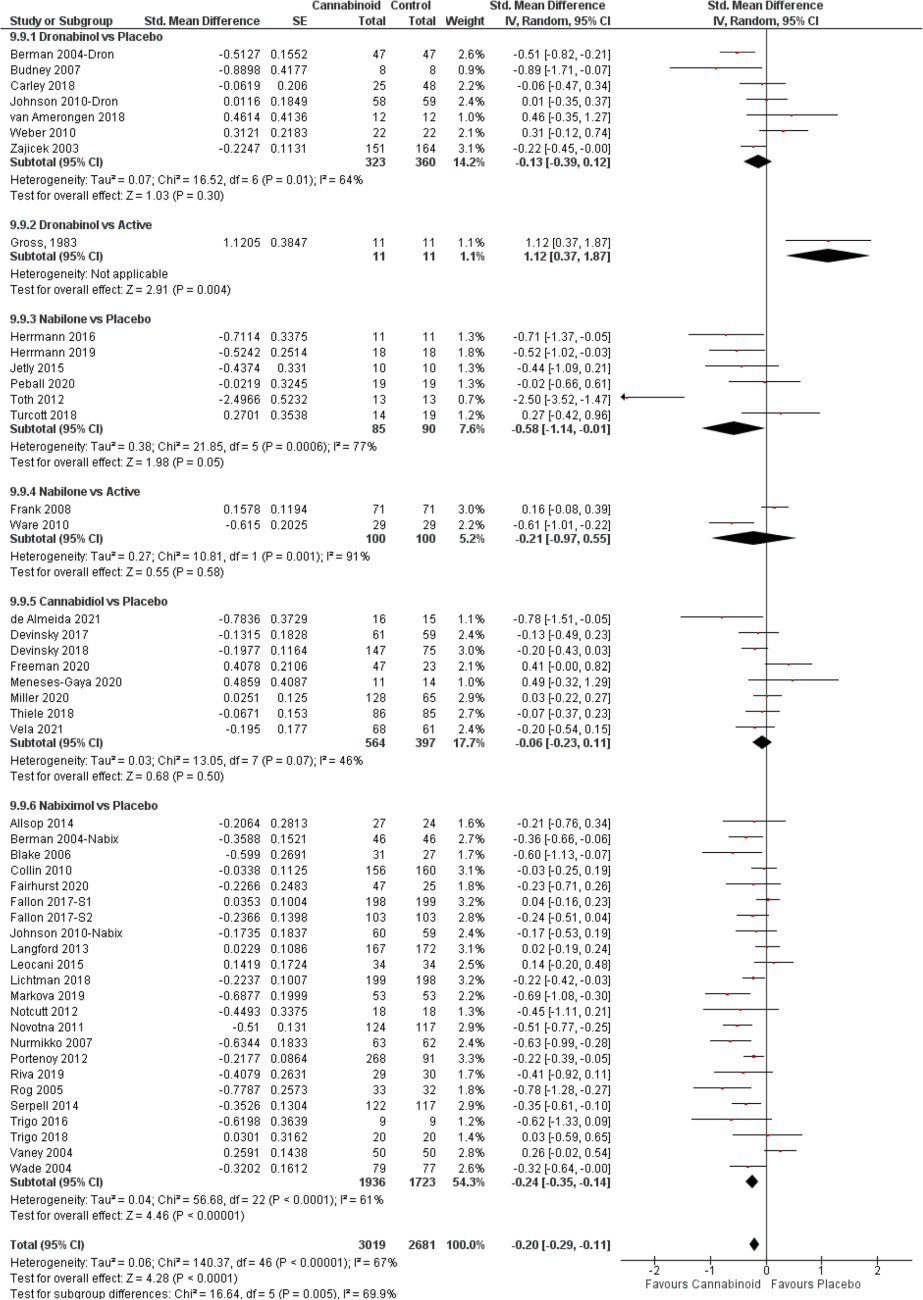
Sleep forest plot, stratified according to cannabinoid type and comparator used. The horizontal lines indicate 95% CIs. The diamond markers represent the subtotal and overall weighed standardized mean difference (SMD) mean difference and 95% CI. The vertical line shows the line of no effect

Several trials included within their outcomes sleep measurements (Fig. 8). From the studies with dronabinol, seven [10, 12, 34, 75, 92, 109, 150] were compared to placebo and one cross-over [100] with diazepam; with nabilone, 6 trials [118, 125, 143, 149, 152, 162] used placebo and two trials [120, 123] used active comparators; and eight CBD [13, 15, 19, 22, 153, 157–159] and 23 nabiximols [10, 33–35, 37, 38, 40, 41, 43, 44, 46–52, 57, 58, 61–63] used placebo. The overall meta-analysis showed a clear improvement in sleep scores (SMD − 0.20, 95% CI − 0.29 to − 0.11; *P* < 0.0001), but also significant subgroup differences (*P* = 0.005). Significant effects favouring cannabinoids were restricted to trials comparing nabilone and nabiximols with placebo. Although nabiximols demonstrated the highest efficacy (SMD − 0.24, 95% CI − 0.35 to − 0.14; *P* < 0.00001) and a moderate quality evidence (Fig. 2B), meta-regression did not indicate a significant superiority versus nabilone (additional file 5, *Q* = 1.96, *P* = 0.1618).

#### Substance abuse

**Fig. 9:**
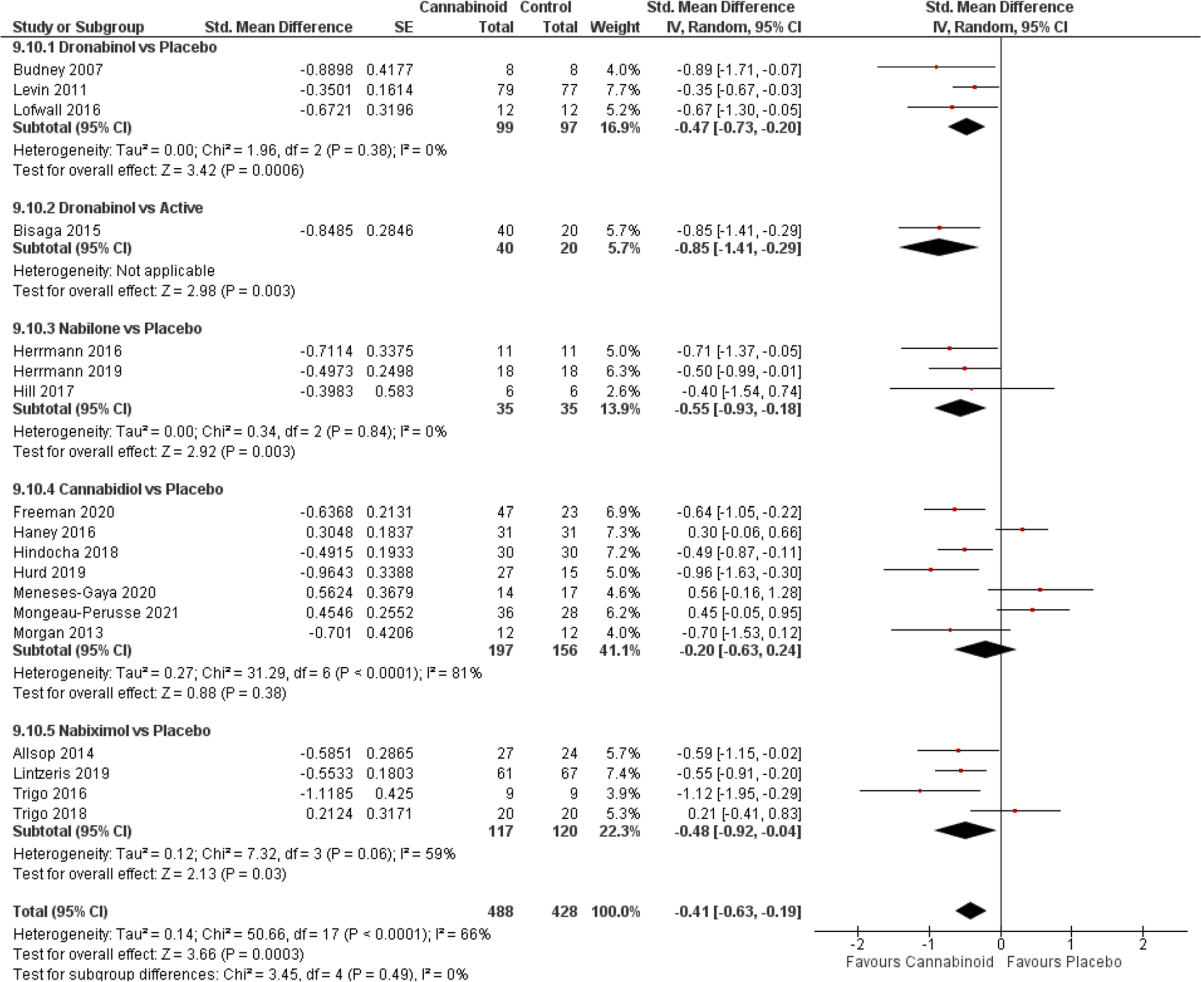
SUDs forest plot, stratified according to cannabinoid type and comparator used. The horizontal lines indicate 95% CIs. The diamond markers represent the subtotal and overall weighed standardized mean difference (SMD) mean difference and 95% CI. The vertical line shows the line of no effect

The overall analysis (Fig. 9) indicates that cannabinoids have a beneficial effect in the treatment of drug dependence (SMD − 0.41, 95% CI − 0.63 to − 0.19; *P* = 0.0003), an effect seen in all subgroup analyses except for CBD [19, 20, 22, 24, 30–32]. Although dronabinol [92, 110, 111] showed the highest efficacy (vs placebo: SMD − 0.47, *P* = 0.0006; vs. active [101]: SMD − 0.85; *P* = 0.003), followed by nabilone [143, 149, 151] (SMD − 0.55, 95% CI − 0.93 to − 0.18; *P* = 0.003), confidence on those results was low and the moderate evidence on the effect estimate was provided only by nabiximols [61–63, 68] (SMD − 0.48, 95% CI − 0.92 to − 0.04; *P* = 0.03) (Fig. 2B). Further meta-regression analysis indicated that the differences in the effect sizes were not related to the cannabinoid type (Additional file 5).

#### Tourette

**Fig. 10:**
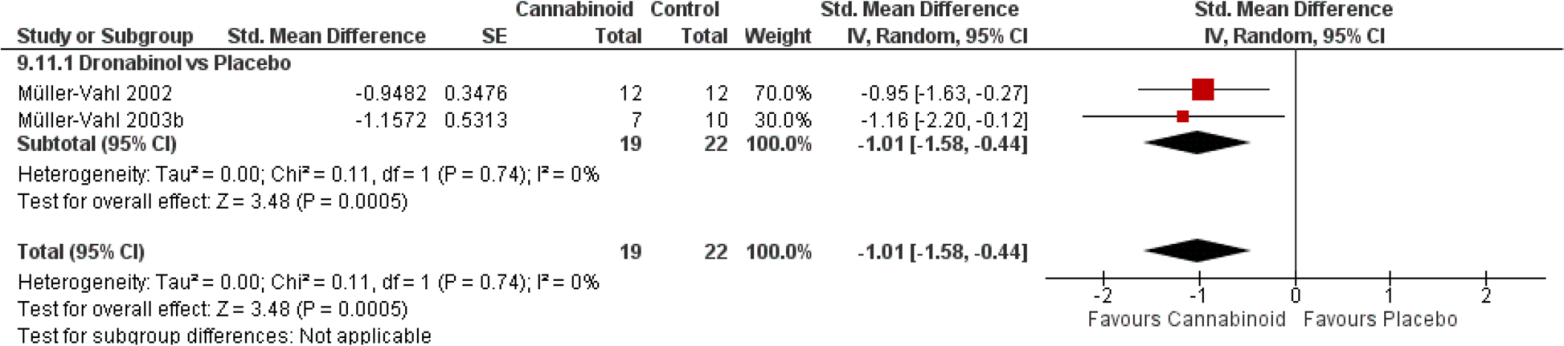
Tourette forest plot. The horizontal lines indicate 95% CIs. The diamond markers represent the subtotal and overall weighed standardized mean difference (SMD) mean difference and 95% CI. The vertical line shows the line of no effect

The two studies [103, 112] reporting the superiority of dronabinol over placebo in attenuating tics severity suggest that dronabinol may be beneficial for Tourette syndrome with a moderate grade of evidence (Fig. 2B) (Fig. 10, SMD − 1.01, 95% CI − 1.58 to − 0.44; *P* = 0.0005).

### Secondary outcomes

Dropouts and adverse events were analysed in 45 trials with dronabinol (37 vs placebo [10–12, 23, 34, 45, 70–76, 79, 81, 83–85, 88, 89, 91, 92, 94–96, 98, 102, 105–110, 112, 139, 150, 161] and 8 vs active comparators [69, 78, 80, 82, 86, 87, 100, 101]), 29 with nabilone (16 vs placebo [114, 118, 119, 124–127, 140, 142, 144–148, 151, 152] and 13 vs active drugs [120, 121, 123, 129, 130, 132–138, 141]) and in 22 and 33 with CBD [13–22, 26, 29, 30, 32, 153–160] and nabiximols [10, 33–38, 40–44, 46–55, 57–60, 64–66, 68, 85, 94], respectively vs placebo (Additional file 6).

#### Retention

Overall retention (Additional file 6: Fig. S22) for all cannabinoids was better in control arms, although not significantly different (OR 1.12, *P* = 0.1). After subgroup analyses, this result remained in CBD-containing medications versus placebo (OR 1.38, 95% CI 0.77 to 2.47 and OR 1.17, 95% CI 0.92 to 1.49) while dronabinol/nabilone subgroups had an almost identical proportion of dropouts in each treatment arm, regardless of the comparator used. The low/very low evidence of these results (Fig. 2B) suggests that retention may be influenced by other or additional factors than the treatment.

#### Adverse events

Despite the fact that the dropout rate in cannabinoid-treated patients does not differ from placebo or active comparators, all cannabinoids produce significant adverse events (Additional file 6: Fig. S23). The evidence was low for dronabinol versus placebo (OR 2.16, 95% CI 1.59 to 2.94; *P* < 0.00001) also in trials using active comparators (OR 2.75, 95% CI 1.43 to 5.26; *P* = 0.002), but nabiximols and nabilone were associated with a high number of participants reporting adverse events in comparison to placebo (nabiximols OR 1.97, 95% CI 1.48 to 2.64; nabilone OR 3.12, 95% CI 1.52 to 6.42). Though nabiximols showed the highest significance (*P* < 0.00001) and CBD the lowest (OR 1.82, 95% CI 1.08 to 3.07; *P* = 0.02), meta-regression analysis did not indicate significant differences (Additional file 5: *Q* = 0.04, *P* = 0.8424). It is also important to consider the severity and the adverse event-related dropouts. That is, severe or serious adverse events were reported only by 4.5% of the CBD trials followed by dronabinol and nabilone (5.4% and 6.3%), dronabinol versus active comparators (12.5%), nabiximols (15.2%) and nabilone versus active comparators with 23.1%; lowest adverse event-related dropouts were found with dronabinol and CBD (24.3% and 27.3%), followed by nabilone (vs placebo 43.8%, vs active comparator 53.8%), nabiximols (54.5%) and dronabinol vs active comparators (62.5%).

## Discussion

Previous SRs and meta-analyses on cannabinoids [1–5] (and many others) did not consider, or only considered via sensitivity analysis, that medical cannabinoids and medical plant-derived cannabis products differ largely in their pharmacological mode of action [6–9] and pharmacokinetics [165]. For the first time, we provide pharmacology-based comparative systematic results for dronabinol, nabilone, CBD and nabiximols for all relevant medical indications. As shown in Fig. 2A, the confidence on the effect estimate strongly differs for these four medications. That is, high quality of evidence is seen only with CBD (6.7% of all CBD trials), and moderate quality of evidence is higher with CBD-containing (CBD 13.3%, nabiximols 37.5%) cannabinoids than with THC-containing (dronabinol 10%, nabilone 4.3%) medications. Notably, these differences are not directly related to a better efficacy, as the proportion of the 152 trials reporting positive results on their primary outcomes did not differ between cannabinoids (dronabinol 52%, nabilone 70%, CBD 52% and nabiximols 57%), resulting in an overall positive effect (data not shown, SMD − 0.33, 95% CI − 0.40 to 0.26; *P* = 0.0004). Although further meta-regression analyses did not show any specific impact of the cannabinoid type, we still found other differences for the four medications. First, CBD shows with a high grade of evidence effectiveness in the treatment of epilepsy (in particular for Dravet syndrome and Lennox-Gastaut syndrome). Second, there is an overall significant effect of cannabinoids on the improvement of chronic pain, but only dronabinol and nabiximols had moderate evidence. Third, although we found an overall significant effect of cannabinoids on appetite stimulation (especially in HIV/AIDS patients), this effect might be driven by dronabinol with a moderate grade of evidence. Fourth, although the overall effect in Parkinson favoured cannabinoids, only CBD seems to have an effect. Fifth, there was an overall significant effect of cannabinoids on improvement in sleep quality and disturbances and this effect was mainly driven by nabiximols. CBD does not improve sleep but the evidence for this is low. Therefore, it is unclear whether the THC or CBD component of nabiximols (because of low or very low evidence) induces this therapeutic effect. Finally, dronabinol and nabilone improves with a low grade of evidence nausea and vomiting due to chemotherapy. However, this effect is only significant in comparison to active comparators such as prochlorperazine that is not well tolerated by patients undergoing chemotherapy [166] and thus speaks against the use of THC-containing medications for the treatment of nausea and vomiting.

A dichotomy of THC vs. CBD-containing medications is also seen with respect to alterations of physiological functions such as appetite in all medical indications. A recent meta-analysis shows that pharmaceutical THC (dronabinol, nabilone) has no negative effect on appetite, whereas CBD decreases appetite (OR = 2.46 [1.74:4.01] with moderate evidence) [167].

In summary, all medical cannabinoid medications differ in their pharmacology, in their therapeutic profile, and in their profile of adverse events.

The strengths of our study are that we performed for the first time a pharmacology-based comparative systematic analysis of medical cannabinoids. Whole plant-derived cannabis products were excluded from our analysis, as those products have a complex and undefined pharmacology. Thus, we also excluded cannabinoid products with undefined mixtures and other non-approved synthetic cannabinoids in order to reduce heterogeneity. We also excluded studies on healthy individuals and studies with no RCT design to reduce heterogeneity and increase the grade of evidence of our interpretations. Finally, data analysis using SMD allowed the inclusion of a large variety of measurements in the evaluation of the outcomes and allowed us to include many more RCTs for all relevant medical indications than in a previous extensive meta-analysis [3].

There are also limitations. One limitation is the exclusion of an important number of studies (15% of all studies, 31% of all comparisons) that were unable to be graded as they are single RCTs for ALS, Chorea Huntington, dystonia, glaucoma, ADHD, anorexia and PTSD, and therefore could not be included in our conclusions (Fig. 2). Due to missing trials, which was especially the case with CBD for many indications, a second limitation is that we were often unable to directly compare all cannabinoid types, which strongly restricted our conclusions. A third limitation is the inclusion of several RCTs with small study sizes. Small study sizes are of particular concern as it has been previously demonstrated that effects are larger in small studies using cannabinoids [2, 168]. Differences in sample characteristics, durations of the trials and doses or route of administration contributed to heterogeneity in some comparisons, thus limiting the confidence on the findings and the meta-analyses results. In this regard, a systematic meta-regression approach adding those variables as covariates was not possible due to the small number of studies.

In conclusion, medical cannabinoids have an overall positive therapeutic effect for epilepsy, chronic pain, spasticity, appetite, Parkinson’s disease, sleep, SUDs and Tourette. Cannabinoids produce significant adverse events and there is low to moderate grade of evidence for this conclusion depending on the type of cannabinoid. Adverse events produced by cannabinoids do not influence retention in clinical trials, as the dropout rate in cannabinoid-treated patients does not differ from placebo or active comparators. CBD trials reported less adverse events than trials with other medical cannabinoids, but regression analysis did not show any significant differences between these medications; noteworthy, CBD trials reported the lowest percentage of serious adverse events (4.5% of all trials compared with 23% of all nabilone trials).

Most importantly, significant therapeutic effects of medical cannabinoids underlie a large variability in the grade of evidence that depends on the type of cannabinoid. Thus, CBD has a significant therapeutic effect for epilepsy and Parkinson’s disease. The grade of evidence for the treatment of CBD for these conditions is high/moderate. There is moderate evidence for dronabinol for the treatment of chronic pain, appetite and Tourette. Moderate evidence is obtained for nabiximols for having significant therapeutic effects on chronic pain, spasticity, sleep and SUDs. All other significant therapeutic effects of medical cannabinoids have either low, very low or even no grade of evidence, which is the case of single RCTs. In conclusion, dronabinol, nabilone, CBD and nabiximols not only differ in their pharmacology but also in their therapeutic profile. Therefore, future SRs and meta-analyses should consider the pharmacology of cannabinoids.

## Conclusions

Cannabinoids are effective therapeutics for several medical indications if their specific pharmacological properties are considered. We suggest that future systematic studies in the cannabinoid field should be based upon their specific pharmacology.

## Methods

Methodological details are provided in Additional file 1 [169–172].

### Study design

This systematic review followed the Preferred Reporting Items for Systematic Reviews and Meta-analyses (PRISMA) guidelines [173] and was registered at PROSPERO (CRD42021229932).

### Search strategy and selection criteria

We searched in eight databases using Medical Subject Heading (MeSH) terms on all literature published until May 2021 (updated in October 2021) separately for dronabinol, nabilone, cannabidiol and nabiximols (Fig. 1, Additional file 2: Table S1) [12, 59, 94, 174–263]. Studies identified by our search that fulfilled the inclusion criteria given below were reviewed by both authors and disagreements were solved through discussion or by consulting colleagues with long-standing expertise in the field of medical cannabinoids. The inclusion criteria were as follows:

- Type of studies: randomized controlled parallel and cross-over trials (RCTs) with allocation concealment that was blinded (single or double blinded) which examined the study objective. We excluded all other study designs, including cohort studies, case control studies, outcome research, case studies, case series, expert opinion and conference abstracts.
- Type of participants: humans of any age or sex, with a medical condition or health problem of any type.
- Types of interventions: four medical cannabinoids: dronabinol, nabilone, cannabidiol and nabiximols for the treatment of any medical condition. We excluded natural cannabis-based formulations (i.e. smoked marijuana). If a study compared one type of cannabinoid to another or one type of cannabinoid with another active drug, we included both arms. The following indications were included: chronic pain; spasticity with multiple sclerosis and paraplegia; nausea, vomiting or loss of appetite; gastroenterological, neurodegenerative and other neurological diseases including: amyotrophic lateral sclerosis, irritable bowel syndrome, multiple sclerosis (tremor and bladder dysfunction), Chorea Huntington, epilepsy, dystonia, Parkinson and glaucoma, and psychiatric disorders including ADHD, anorexia nervosa, anxiety disorders, dementia, depression, psychotic disorders and schizophrenia, PTSD, sleeping disorders, substance abuse disorders and Tourette.
- Types of outcomes measures: Eligible outcomes were patient-important and disease-specific outcomes (primary outcomes), retention and adverse events (secondary outcomes).

Data were extracted based on the PICO (Population, Intervention, Comparator and Outcome) format. Risk of bias was assessed using the Cochrane Collaboration’s tool for assessing risk of bias as outlined in the Cochrane Handbook for Systematic Reviews of Interventions [169] and contained in Review Manager (RevMan) version 5.4.1. (The Cochrane Collaboration, 2020). Grading of evidence was assessed using GRADEpro [170]. Both assessments were completed independently by both reviewer authors.

### Data synthesis and statistical analysis

All analyses were conducted using Review Manager (RevMan) version 5.4.1. (The Cochrane Collaboration, 2020). Dichotomous and continuous outcomes were pooled as odds ratios (ORs) and standardized mean difference (SMD), respectively using random effects. For cross-over trials, SMD and SE were calculated with the correlation coefficient estimated at 0.5, according to the Becker-Balagtas marginal method [171]. Heterogeneity was assessed using the *I*^*2*^ statistic. Analyses were stratified by outcome and conducted with subgroup analyses by cannabinoid type and comparator. For direct comparisons between two subgroups, meta-regression was performed using type of cannabinoid as covariate.

## Supplementary Information

**Additional file 1. **Methodological details.

**Additional file 2. **Abbreviations and characteristics of excluded and included studies.

**Additional file 3. **Risk of bias assessments of included studies.

**Additional file 4. **Forest-plot for primary outcomes.

**Additional file 5. **Meta-regression analysis.

**Additional file 6. **Forest-plot for secondary outcomes: retention and adverse events.

## Data Availability

The datasets used and/or analysed during the current study available from the corresponding author (AB) on reasonable request.

## Abbreviations

ADHD: Attention deficit and hyperactivity disorder
AIDS: Acquired immunodeficiency syndrome
ALS: Amyotrophic lateral sclerosis
CB1: Cannabinoid receptor type 1
CB2: Cannabinoid receptor type 2
CBD: Cannabidiol
CI: Confidence interval
HIV: Human immunodeficiency virus
MeSH: Medical Subject Heading
OR: Odds ratio
PANNS: Psychiatric Assessments Psychotic symptoms
PICO: Population, Intervention, Comparator and Outcome
PTSD: Post-traumatic stress disorder
RCT: Randomized controlled trial
SE: Standard error
SMD: Standardized mean difference
SR: Systematic review
SUD: Substance use disorder
THC: ( −)-*trans*-Δ^9^-Tetrahydrocannabinol
