# Supplementary material for: Medical cannabinoids: a pharmacology-based systematic review and meta-analysis for all relevant medical indications
Source: BMC Med. 2022 Aug 19;20:259. doi: 10.1186/s12916-022-02459-1 (PMC9389720; doi:10.1186/s12916-022-02459-1)
Supplement: Supplementary file 1 — Additional file 1. Methodological details. [file 12916_2022_2459_MOESM1_ESM.docx]

**Methodological details**

**Search Strategy**

-Electronic searches:

- PubMed (https://pubmed.ncbi.nlm.nih.gov/)
- Embase 8https://www.embase.com/landing?status=grey)
- PsychoINFO (https://www.apa.org/pubs/databases)
- Cochrane database of Reviews (https://www.cochranelibrary.com/cdsr/reviews)
- Cochrane Central Register of Controlled Trials (CENTRAL) (https://www.cochranelibrary.com/central)
- Medline (OvidSP)
- ERIC (OvidSP) (Education Resources Information Center)
- Ovid journals (journals@Ovid LWW Journal Definitive Archive Collection)

-Medical Subject Heading (MeSH) terms on all literature published until May 2021. Four separate searches were conducted by using the following MeSH terms:

- Epidiolex OR Epidyolex OR CBD OR cannabidiolum OR cannabidiol.
- Dronabinol OR Marinol OR Syndros.
- Nabilone OR Cesamet OR Canemes.
- Nabiximols OR Sativex OR THC: CBD OR THC/CBD

-Restrictions: English or German language, RCTs, SRs, or meta-analyses as type of studies and human species.

*-*Searching other resources: Reference lists of included studies, conference abstracts and systematic reviews or meta-analyses for potentially relevant articles.

**Assessment of risk of bias and grading of evidence**

The risk of bias includes the following seven items:

- Random sequence generation (selection bias): selection bias (biased allocation to interventions) due to inadequate generation of a randomised sequence;
- Allocation concealment (selection bias): selection bias (biased allocation to interventions) due to inadequate concealment of allocations prior to assignment;
- Blinding of participants and personnel (performance bias): performance bias due to knowledge of the allocated interventions after assignment;
- Blinding of outcome assessment (detection bias): detection bias due to knowledge of the allocated interventions after assignment;
- Incomplete outcome data (attrition bias): attrition bias due to amount, nature of handling of incomplete outcome data;
- Selective reporting (reporting bias): reporting bias due to selective outcome reporting; and
- Other bias: bias due to problems not covered elsewhere.

The GRADE approach begins with the study design, where RCTs provide high quality of evidence, and then addresses five reasons to possibly rate down the quality of the body of evidence for each outcome: study limitations, inconsistency, indirectness, imprecision, and publication bias. The GRADE system uses the following criteria for assigning grade of evidence:

- High: This research provides a very good indication of the likely effect. The likelihood that the effect will be substantially different is low.
- Moderate: This research provides a good indication of the likely effect. The likelihood that the effect will be substantially different is moderate.
- Low: This research provides some indication of the likely effect. However, the likelihood that it will be substantially different (a large enough difference that it might have an effect on a decision) is high.
- Very low: This research does not provide a reliable indication of the likely effect. The likelihood that the effect will be substantially different (a large enough difference that it might have an effect on a decision) is very high.

Outcomes can be downgraded a maximum of 3 levels (serious (-1) or very serious (-2)) according to the following factors that can reduce the quality of the evidence:

- Study limitation (risk of bias): limitations in the study design and execution that may bias the estimates of the treatment effect.
- Inconsistency of results: referring to an unexplained heterogeneity of results.
- Indirectness of evidence: research that directly compares the interventions delivered to the populations in which we are interested, and measures the outcomes important to patients.
- Imprecision: when studies include relatively few patients and few events and thus have a wide confidence interval (CI) around the estimate of the effect.
- Publication bias: systematic under-estimation or an over-estimation of the underlying beneficial or harmful effect due to the selective publication of studies.

**Data analysis**

All analyses were conducted using Review Manager (RevMan) version 5.4.1. (The Cochrane Collaboration, 2020). Dichotomous outcomes such as retention (number of participants not completing the trial) and adverse events (number of participants experiencing any type of adverse event) were pooled as odds ratios (ORs) using random effects, Mantel-Haenszel meta-analyses. For continuous outcomes, we used generic inverse variance and random effects.

The effect sizes were calculated from the differences in change from baseline when available, or from post-treatment differences, and were pooled as standardised mean difference (SMD) and the corresponding standard error (SE). Meta-analyses included parallel and cross-over RCTs. For cross-over trials, SMD and SE were calculated with the correlation coefficient estimated at 0.5, according to the Becker-Balagtas marginal method. Subgroups within parallel studies were combined into a single group, and in the case of cross-over studies, the most effective dose was included. When several outcomes measures were available within one study, we included the most regular method commonly used by other studies. The time point selected was at the end of the treatment, and no follow-up time points after treatment cessation were included. Heterogeneity was assessed using the *I^2^* statistic. The following rule of thumb for determining what constituted a large *I^2^* value was used: *I^2^* values of < 40%, 30-60%, 50-90% and 75-100% can be considered low, moderate, substantial and considerable levels of inconsistency across studies, respectively.

Analyses were stratified by outcome, and conducted with subgroup analyses by cannabinoid type (dronabinol, nabilone, cannabidiol and nabiximol) and comparator (placebo or active). We first performed a general estimates analysis, by pooling the evidence from all eligible RCTs, regardless of population or health condition, for the outcomes of efficacy (primary outcome or patient-important outcome) retention (number of participants not completing the trial) and adverse events (number of participants experiencing any type of adverse event). We then conducted 21 primary analyses including the following outcomes: chronic pain; spasticity; nausea/vomiting, appetite, amyotrophic lateral sclerosis, irritable bowel syndrome, multiple sclerosis, Chorea Huntington, epilepsy, dystonia, Parkinson, glaucoma, ADHD, anorexia nervosa, anxiety, dementia, depression, psychosis and schizophrenia, PTSD, sleep, substance abuse and Tourette.
