# Supplementary material for: Medical cannabinoids: a pharmacology-based systematic review and meta-analysis for all relevant medical indications
Source: BMC Med. 2022 Aug 19;20:259. doi: 10.1186/s12916-022-02459-1 (PMC9389720; doi:10.1186/s12916-022-02459-1)
Supplement: Supplementary file 2 — Additional file 2. Abbreviations and characteristics of excluded and included studies. [file 12916_2022_2459_MOESM2_ESM.docx]

**List of abbreviations**

2/10MWT: 2/10-meter walking test

9HPT: 9-Hole Peg Test

AC/S: Anorexia/Cachexia Scale

AD: Alzheimer's disease

ADHD: Attention Deficit Hypercativity Disorder

ADL: Activity Daily Living

ALS: Amyotrophic Lateral Sclerosis

ALSFRS: Amyotrophic Lateral Sclerosis Functional Rating Scale

AN: Anorexia Nervosa

BACS: Brief Assessment of Cognition in Schizophrenia

BAI: Beck Anxiety Inventory

BBS: Berg Balance Score

BDI: Beck Depression Inventory

BMI: body mass index

BPI: Brief Pain Inventory

BPI-SF: Brief Pain Inventory-Short Form

BPRS: Brief psychiatric rating scale

BS-11: 11-point box scale

BSI: Brief Symptom Inventory

BSS: Bodily Symtoms Scale

BVRT: Benton-visual-retention-test

CAARS: Conners Adult ADHD Rating Scale

CADSS: clinical-administered dissociative symptoms scale

CAPS: Clinician-Administered PTSD Scale

CBD: Cannabidiol

CCGIC: Caregiver Clinical Global Impression of Change

CCQ-Brief: Cocaine Craving Questionnaire – Brief

CGI: Clinician's Global Impression

CGIC: Caregiver Global Impression of Change

CGICSD: Caregiver Global Impression of Change in Seizure Duration

CGI-I/S: Improvement/Severity score on the Clinical Global Impressions Scale

CMAI: Cohen-Mansfield Agitation Inventory

CNP: chronic neuropahtic pain

CP: chronic pain

CSSA: Cocaine Selective Severity Assessment

CWS: Cannabis withdrawal scale

EDI-2: Eating Disorder Inventory -2

EDSS: expanded disability status scale score

EORTC: European Organisation for Research and Treatment of Cancer

EPRS: Emetic Process Rating Scale

ESAS: Edmonton Symptom Assessment System

ESPID: evoqued pain differences

ESS: Epworth Sleepiness Scale

FAACT: functional assessment of anorexia/cachexia therapy

FIQ: fibromyalgia imparct questionnaire

FNE: Fear of Negative Evaluation Questionnaire

GAAQ: Goldberg Anorectic Attitude Questionnaire

GAF: Global Assessment of Functioning scale

GHQ-12: General Health Questionnaire-12

GIC: Global Impression of Change

GSI: General symptomatic index

HADS: Hospital Anxiety and Depression Scale

HAM-A: Hamilton Anxiety Scale

HAM-D: Hamilton Depression Scale

HAQ-DI: Health Assessment Questionnaire

HI: headache index

HIV: human immunodeficiency virus

HRQL: Health-related Quality of Life

HSCL-90: Hopkins Symptom Checklist-90

HSQ: Hunger-Satiety Questionnaire

IBS: Irritable Bowel Disease

INV-2: Index of Nausea and Vomiting Form 2

ISIS: Insomnia Severity Index Scale

KPPS: King's PD Pain Scale

LSAS: Liebowitz Social Anxiety Scale

LSEQ: Leeds Sleep Evaluation Questionnaire

M and Q: Marsden and Quinn's chorea severity evaluation scale

MAS: Modified Asworth Scale

MBPI: Modified Bried Pain Inventory

MCCB-T: T score of MATRICS Consensus Cognitive Battery

MCCS: Minnesota Cocaine Craving Scale

MCQ: Marijuana Craving Questionnaire

MDS-UPDRS-I: Movement Disorder Society-Unified PD Rating Scale-I

MFIS: Modified Fatigue Impact Scale

MOSSS: Medical Outcomes Study Sleep Scale

MPQ: McGill Pain QUestionnaire

MPSS: Mood and Physical Symptoms Scale

MRS: Mood Rating Scale

MS: Multiple Sclerosis

MSFC: Multiple sclerosis functional composite

MSIS-29: Multiple Sclerosis Impact Scale

MSIS-29phys: Multiple sclerosis impact physical subscale

MSSS-88: Multiple sclerosis spasticity scale-88

MSWS-12: multiple sclerosis walking scale

MTS: Modified Tardieu Scale

MWC: Marijuana Withdrawall Checklist

MWT-B: Multiple choice vocabulary test

NCCTG: North Central Cancer Treatment Group questionnaires

NMA-SF: Mini-Nutritional Assessment Short-Form

NMSS: Non-motor symptoms

NP: Neuropathic Pain

NPI: Neuropsychiatric inventory score

NPI-NH: neuropsychiatric inventory- nursing home

NPS: Neuropathic pain scale

NPSI: Neuropathic Pain Symptom Inventory

NRS: numerical rating scale

OBC: Overall Bladder Condition

OOWS: Objective Opiate Withdrawal Scale

PAIN-AD: Pain Assessment in Alzheimer's disease

PANSS: Psychiatric Assessments Psychotic symptoms

PAS: Parkinson Anxiety Scale

PCS: Pain Catastrophizing Scale

PD: Parkinson's Disease

PDI: Pain Disability Index

PDQ-39: Parkinson’s Disease Questionnaire – 39

PDSS: Parkinson Disease Sleep Scale

PGIC: Patient's or Physician's Global Impression of change

PI: Pain intensity

POMS: Profile of Modd States

PONV: post-operative nausea and vomiting

PPP: paedriatic pain profile

PR50: Pressure at half-maximun volume

PSDI: positive symtom distress intdex

PSQ: Patient Satisfaction Questionnaire

PSQI: Pittsburg Sleep Quality Index

PST: positive symptom total

PTSD: Post-traumatic stress disorder

PTSS: Pain Treatment Satisfaction Scale

Qb Test: Quantitative Behavioral Test

QLQ: Quality of Life Questionnaire

QST: quantitative sensory testing

QSU-B: Questionnaire of Smoking Urges–Brief

QUIDS: Quick Inventory for Depressive Symptoms

RBDSQ: REM Sleep Behaviour Disorder Screening Questionnaire

RDDS: Rush Dyskinesia Disabiity Scale

REM: Rapid Eye Movement

RMA: Rivermead Motor Assessment

RMI: Rivermead mobility index

SANS: Scale for Assessment of Negative symptoms

SART: Sustained Attention to Response Task

SAS: Self-rating anxiety Scale

SCL-56/90-R: Sympom Checklist-56/90 Revised

SDS: Goldberg Situational Discomfort Scale

SF-36: Short Form Health Survey

SF-MPQ: short form McGill questionnaire

SGIC: Subject's Global Impression of change

SIB: severe impariment battery

SLIM: Satiety Labeled Intensity Magnitude scale

SMHSQ: St Mary's Hospital Sleep Questionnaire

sMMSE: standardized Mini-Mental Status Exxamination

SOWS: Subjective Opiate Withdrawal Scale

SPID: sum of pain intensity difference

SQD: Sleep Disorder Questionnaire

SS: Sleep Scale

SSPS-N: Self-Statements during Public Speaking, negative subscale

SSS: Spasticity sum score

STAI: State-Trait Anxiety Inventory

STAI-S: State Trait Anxiety Inventory state subscale

SUDS: Substance Use Disorders

T10/25FW: Timed 10/25-Foot Walk Test

TCQ:11: Tiffany Craving Questionnaire

TLFB: Timeline Follow Back interview

TOTPAR: Total Pain Relief

TSSL: Tourette`s syndrome Symptom List

TUG: Timed Up Go Test

TWSTRS(-C): Toronto Western Spasmodic Torticollis Rating Scale (-C: pain)

UHDRS: Unified Huntington's Disease Rating Scale

UPDRS: Unified Parkinson's disease rating scale

VAMS: Visual Analogue Mood Scale

VAP: Visual Analog of Pain

VAS: Visual analogue scale

VPA: Verbal paired associates

WDS: Withdrawal Discomfort Score

WRAADS: Wender-Reimherr Adult Attention Deficit Disorder Scale

ZAS/ZDS: Zung Depression and Anxiety Scales

ZBI: Zarit Burden Interview

**Characteristics of included studies**

**Dronabinol**

Of the 53 RCTs (57 articles, note that 1) Zajicek 2003, Zajicek 2013, Müller-Vahl 2001, Müller-Vahl 2003 and Andries 2014 belong to the same RCT as Freeman 2006, Ball 2015, Müller-Vahl 2002, Müller-Vahl 2003b and Andries 2015, respectively and that 2) Gilbert 1995 contains 2 RCTs) included in the review, 43 used placebo (2755 participants) and 10 (947 participants) used an active comparator, the latter mostly included in the nausea, vomiting and loss of appetite indication. Twenty-four studies (2796 participants) were parallel trials and 29 (916 participants) were cross-over trials with each type of design including nearly equal proportion of indications.

As shown in Table S2, among the indications with the largest number of studies are chronic pain with 17 RCTs, spasticity with 6 RCTs and 7 articles, nausea, vomiting and appetite with 29 RCTs, anxiety with 10 RCTs and 11 articles, depression with 10 RCTs and 11 articles and sleep with 10 RCTs. Few RCTs have been performed in gastroenterological, neurodegenerative, and other neurological diseases including amyotrophic lateral sclerosis , dystonia, glaucoma, IBS, MS (2 RCTs, 3 articles) and in psychiatric conditions like anorexia (2 RCTs, 3 articles), dementia, PTSD, psychosis, substance abuse and Tourette (2 RCTs, 3 articles). There are no RCTs in patients with Huntington chorea, epilepsy, Parkinson or ADHD.

**Nabilone**

Of the 35 RCTs included in the review, 21 used placebo (942 participants) and 14 (603 participants) used an active comparator, the latter mostly included in the nausea, vomiting and loss of appetite indication. Twelve studies (738 participants) were parallel trials and included mainly nausea, vomiting, appetite and pain outcomes and 23 (807 participants) were cross-over trials which included all indications.

Table S3 shows the number of studies for each indication using nabilone. As observed, a moderate evidence -in terms of number of published RCTs- has been restricted to few indications. In particular, to 12 RCTs for chronic pain, 17 RCTs for nausea, vomiting and appetite, 10 RCTs on anxiety, 7 RCTs with scores for depression and 9 RCTs assessing sleep. In other medical conditions, trials using nabilone have been very occasional, as with spasticity, chorea Huntington dystonia, Parkinson’s disease, dementia, PTSD and substance abuse disorders. There are no RCTs in patients with epilepsy, glaucoma, irritable bowel syndrome, MS, ADHD, anorexia nervosa, schizophrenia/psychosis and Tourette.

**Cannabidiol**

Of the 27 RCTs included in the review, 26 used placebo (1802 participants) and 1 (42 participants) used an active comparator in schizophrenic patients. Twenty-one studies (1702 participants) were parallel trials and 6 (142 participants) were cross-over trials, the latter conducted in substance abuse (n=2), Parkinson (n=1), schizophrenia (n=1), chorea Huntington (n=1) and anxious (n=1) patients.

The literature on cannabidiol is relatively young, as virtually all RCTs (except for two) have been published in the last 11 years. As shown in Table S4, from the twenty-one possible medical indications, cannabidiol has only been tested in 10 of them. Individual studies have assessed the effect of cannabidiol in chronic pain, appetite and chorea Huntington. The effect of cannabidiol on epilepsy was first reported by an early and small study and more recently has been further documented by a series of publications belonging to a large international clinical trial. Parkinson’s disease symptoms have been investigated in recent trials including one cross-over and two parallel studies. In the field of psychiatry, anxiety has been extensively assessed in eleven RCTs; indeed, it represents the medical condition with the highest number of studies carried out not only within cannabidiol studies, but also when compared to the other cannabinoids. Of note, in four of the 11 studies, anxiety was a primary outcome and included participants with a diagnosed anxiety disorder or/and an anxiety task. The other seven include pathologies with a high anxiety component like substance abuse, sleep disorders (104) , psychosis or arhtritis. Some of the studies mentioned above assessing anxiety have also tested depressive symptoms together with another trial. There are four studies in schizophrenic and psychotic patients comparing the effect of cannabidiol versus placebo and one versus an active comparator. Sleep outcomes have been evaluated in nine studies. Four of them included participants with epilepsy, two included substance abuse disorders, one schizophrenic patients, one arthiritic patients and in one sleep was a main outcome. Lastly, the efficacy of cannabidiol has been tested during the last 10 years for substance abuse disorders in six parallel and one cross-over trials. There are no RCTs in patients with spasticity, dystonia, glaucoma, irritable bowel syndrome, MS, ADHD, anorexia nervosa, dementia, PTSD and Tourette.

**Nabiximols**

All the 37 RCTs (36 articles, note that Fallon 2017 contains 2 RCTs) included in the review used placebo. Twenty-seven studies (4130 participants) were parallel trials and 10 (333 participants) were cross-over trials, the latter conducted for spasticity (n=3), pain (n=3), glaucoma (n=1), chorea Huntington (n=1), substance abuse (n=1) and nausea, vomiting and appetite (n=1) primary outcomes.

As shown in Table S5, twenty-one of the 37 RCTs have assessed chronic pain being a primary outcome in fourteen of them. Patients characteristics included: cancer, cerebral palsy, diabetes, amyotrophic lateral sclerosis, MS, neuropathic pain and arthritis. Of these, only four were cross-over studies. Nabiximol has been the main cannabinoid used (among dronabinol, nabilone or cannabidiol) to treat spasticity as reflected by the fourteen published trials. Two of them have been conducted in patients with cerebral palsy and amyotrophic lateral sclerosis and the other in MS. Nausea, vomiting and loss of appetite outcomes have been reported in cancer patients receiving chemotherapy or with anorexia-cachexia syndrome in four studies, in patients with substance abuse disorders in three studies and in amyotrophic lateral sclerosis in one study. There are some RCTs performed in gastroenterological, neurodegenerative, and other neurological diseases including amyotrophic lateral sclerosis, chorea Huntington and dystonia, both assessed by one cross-over study, glaucoma and bladder and tremor symptoms in MS reported by five trials. From the ten psychiatric indications selected in this review, nabiximol has been tested in 5 of them. A single and recent RCT with 30 participants has been carried out in ADHD patients. Six RCTs have measured anxiety as secondary outcomes in cannabis dependence, MS and chorea Huntington. In the same way, depressive mood was included as secondary outcome in eight trials involving cannabis dependent, MS and chorea Huntington patients. A large proportion of the studies, twenty-three out of 37, have included sleep measurements in their trials (Table S5) involving substance abuse (n=3), MS (n=9), cancer (n=5), pain (n=3), amyotrophic lateral sclerosis (n=1), arthritis (n=1) and cerebral palsy (n=1). Finally, the efficacy of nabiximol in substance abuse disorders has been tested in cannabis dependent patients in three parallel and one cross-over studies. There are no RCTs in patients with epilepsy, spasticity, irritable bowel syndrome, Parkinson, anorexia nervosa, dementia, PTSD, schizophrenia or psychosis and Tourette.

**SupplementaryTable 1. List of excluded studies with reasons**

| **Dronabinol (n=32)** | **Reason** | **Explanation** |
| --- | --- | --- |
| Abrams et al., 2003 | Outcome | Pharmacokinetic |
| Almog et al., 2020 | Intervention | Cannabis |
| Andries et al., 2015b | Design | Secondary analysis |
| Attal et a l., 2004 | Design | Open label |
| Baker et al., 2018 | Intervention | Buspirone, vilazodone |
| Bedi et al., 2010 | Design | Laboratory experimental |
| Bonn-Miller et al., 2021 | Intervention | Smoked cannabis |
| Bredt et al., 2002 | Outcome | Molecular/genetic markers |
| Brezing et al., 2018 | Design | Secondary analysis |
| Buggy et al., 2003 | Participants | Acute pain |
| Chang et al., 1981 | Intervention | Dronabinol plus THC cigarette |
| Chang et al-. 1979 | Intervention | Dronabinol plus THC cigarette |
| Freeman et al., 2015 | Participants | No- clinical paranoid ideations |
| Haney et al., 2008 | Participants | Volunteers smokers |
| Issa et al., 2014 | Design | Secondary analysis |
| Jansma et al., 2013 | Outcome | Monetary reward reaction |
| Jatoi et al., 2002b | Design | Secondary analysis |
| Jicha et al., 2015 | Outcome | Pharmacokinetic |
| Karschner et al., 2011 | Outcome | Subjective effects of the test drug |
| Lane et al., 1990 | Design | Partial report of data |
| Levin et al., 2016 | Intervention | Dronabinol plus lofexidine |
| McCabe et al., 1988 | Design | Not blinded |
| Merritt et al., 1981 | Design | Laboratory experimental |
| Prasad et al., 2013 | Design | No placebo-controlled |
| Reichenbach et al., 2015 | Outcome | Metabolic parameters |
| Schlienz et al., 2018 | Design | Laboratory experimental |
| Ungerleider et al., 1985 | Design | Secondary analysis |
| van den Elsen et al., 2017 | Design | Secondary analysis |
| Wade et al., 2003 | Intervention | THC-rich, does not specify concentration |
| Wallace et al., 2015 | Intervention | Inhaled cannabis |
| Wong et al., 2012 | Design | Secondary analysis |
| Zajicek et al., 2012 | Intervention | Cannador |
| **Nabilone (n=16)** | **Reason** | **Explanation** |
| Lile et al., 2011 | Outcome | Discrmination effect |
| Wade et al., 2003 | Intervention | THC-rich, CBD-rich |
| Williams et al., 1980 | Design | No placebo-controlled |
| Bedi, et al., 2013 | Outcome | Subjective effects of the test drug |
| Cunningham et al., 1988 | Design | Short report |
| Cunningham et al., 1985 | Design | No placebo-controlled |
| Duran et al., 2010 | Intervention | Nabiximols |
| Fraser, 2009 | Design | Report from an open label |
| **Nabilone (cont)** | **Reason** | **Explanation** |
| Jones et al., 1982 | Design | Symposium abstract |
| Kalliomäki et al., 2012 | Participants | Healthy |
| Kayser et al., 2020 | Design | Trial description |
| Nakano et al., 1978 | Participants | Healthy |
| Niiranen and Mattson, 1987 | Design | No placebo-controlled |
| Notcutt et al., 2004 | Design | N of 1 |
| Peball et al., 2019 | Design | Protocol |
| Steele et al., 1980 | Not found |  |
| **Cannabidiol (n=33)** | **Reason** | **Explanation** |
| Appiah-Kusi et al., 2020 | Design | Case study |
| Ben-Menachem et al., 2020 | Outcome | Pharmacokinetic |
| Bhattacharyya et al., 2018 | Participants | High risk of psychosis |
| Birnbaum et al., 2019 | Design | Open label |
| Bristot et al., 2020 | Design | Conference abstract |
| Davies et al., 2020 | Participants | High risk of psychosis |
| Devinsky et al., 2018 | Outcome | Safety |
| Efron et al., 2020 | Intervention | Not pure (98%) oil |
| Good et al., 2019 | Design | Protocol |
| Hardy et al., 2020 | Design | Protocol |
| Hindocha et al., 2015 | Outcome | Emotional processing |
| Hundal et al., 2018 | Participants | High trait paranoid group |
| Hussain et al., 2020 | Design | Open label |
| Irving et al., 2018 | Intervention | CBD-rich, with other compounds |
| Klein et al., 2019 | Outcome | Pharmacokinetic |
| Leweke et al., 2018 | Design | Protocol |
| Müller-Vahl, 2003 | Design | Review |
| Naftali et al., 2017 | Intervention | Lebanese hashish (99,5% pure) |
| Nitecka-Buchta et al., 2019 | Intervention | Hemp extract oil with other cannabinoids |
| Santos de Alencar et al., 2021 | Outcome | Tremor |
| Szaflarski et al., 2019 | Design | Open label |
| Szaflarski et al., 2017 | Design | Secondary analysis |
| Thiele et al., 2021 | Design | Abstrac poster |
| Tomida et al., 2006 | Intervention | CBD contained a small amount of THC |
| van Amsterdam et al., 2018 | Intervention | Smoked cannabis |
| Wade et al., 2003 | Intervention | THC-rich, does not specify concentration |
| Wall et al., 2019 | Participants | Healthy |
| Wheless et al., 2019 | Outcome | Pharmacokinetic |
| Wilson et al., 2019 | Participants | High risk of psychosis |
| Winton-Brown et al., 2011 | Participants | Healthy |
| Wright et al., 2012 | Design | Secondary analysis |
| Xu et al., 2020 | Intervention | CBD-rich, does not specify concentration |
| Zajicek et al., 2003 | Intervention | Marinol and Cannador |
| **Nabiximols (n=17)** | **Reason** | **Explanation** |
| Centonze et al., 2009 | Design | No placebo-controlled, randomized |
| Flachenecker et al., 2014 | Design | Observational |
| Haupts et al., 2016 | Design | Secondary analysis |
| Hindocha et al., 2015 | Outcome | Emotional processing |
| Hindocha et al., 2020 | Outcome | Genotype comparison |
| Issa et al., 2014 | Design | Secondary analysis |
| Libzon et al., 2018 | Design | No placebo-controlled |
| Lintzeris et al., 2020 | Design | Secondary analysis |
| Lus et al., 2018 | Design | No placebo-controlled |
| Marinelli et al., 2017 | Design | Protocol |
| Meuth et al., 2020 | Design | Secondary analysis |
| Naftali et al., 2021 | Intervention | Avidekel |
| Schoedel et al., 2011 | Outcome | Abuse potential of the study drug |
| Trigo et al., 2016 | Design | Case series |
| van de Donk et al., 2019 | Intervention | Cannabis |
| Wade et al., 2006 | Design | Open label |
| Wade et al., 2003 | Intervention | CBD:THC, does not specify concentration |

**Supplementary Table 2. Characteristics of included RCTs with dronabinol**

| **Disorder** | **Study** | **№ of participants (treated)** | **Sample characteristics** | **Intervention** | **Comparison** | **Outcome or method (s)** |
| --- | --- | --- | --- | --- | --- | --- |
| **Chronic Pain** | | | | | | |
| Cancer | Johnson 2010 | 117 (58) | Incurable malignancy | Oromucosal THC, max. 48 sprays (129.6 mg)/d x 2w | Placebo | NRS, BPI-SF, responders |
|  | Noyes 1975 | 36 (36)* | Diverse cancer types | THC, 10 mg and 20 mg acute | Placebo | VAS^P^, responders^P^ |
| Cervical dystonia | Zadikoff 2011 | 9 (9)* | Cervical dystonia | Marinol, 15 mg/d x 8w | Placebo | TWSTRS-C, VAP |
| Chronic pancreatitis | De Vries 2017 | 65 (31) | Abdominal pain and postsurgical pain | Namisol, 8 mg/d x 50-52d | Placebo | VAS, questionnaires, PGIC |
|  | De Vries 2016 | 25 (25)* | Chronic abdominal pain | Namisol, 8 mg acute | Placebo | VAS |
| IBS | Wong 2011 | 75 (48) | IBS-related pain | Dronabinol, 2.5 mg and 5 mg acute | Placebo | VAS |
| MS | Ball 2015 | 498 (332) | Primary or secondary progressive MS | THC, max. 28 mg/d x 144 -168w | Placebo | MSSS-88 |
|  | Schimrigk 2017 | 240 (124) | MS with CNP | Dronabinol, 7.5 -15 mg x 16w | Placebo | NRS |
|  | Svendsen 2004 | 24 (24)* | Central NP | Marinol, max. 10 mg/d x 18-21d | Placebo | NRS^P^, responders, preference |
|  | van Amerongen 2018 | 24 (12) | Progressive MS | Namisol, 9-29 mg/d x 4w | Placebo | NRS, diary |
|  | Zajicek 2003 | 438 (216) | MS with problematic spasticity | Marinol, max. 25 mg/d x 14w | Placebo | Rating^P^, questionnaire^P^ |
| Non cardiac chest pain | Malik 2017 | 19 (10) | Esophageal hypersensitivity | Dronabinol, 10 mg/d x 4w | Placebo | Questionnaire^P^, VAS^P^ |
| NP | Berman 2004 | 48 (48)* | Brachial plexus avulsion | Oromusocsal THC, max. 48 sprays (129.6 mg)/d x 2w | Placebo | BS-11^P^, SF-MPQ^P^, PDI^P^, GHQ-12^P^ |
|  | Narang 2008 | 30 (30)* | Neuropathic, nociceptive, mixed pain | Marinol, 10 mg and 20 mg acute | Placebo | TOTPAR^P^, SPID^P^, ESPID^P^ |
|  | Weizman 2018 | 15 (15)* | Chronic lumbar radicular pain | Sublingual THC, 15.4 + 2.2 mg acute | Placebo | VAS^P^ |
| Spinal cord injury | Hagenbach 2007 | 13 (6) | Paraplegic and tetraplegics | Marinol, 15-60 mg/d x 6w | Placebo | Rating |
|  | Rintala 2010 | 14 (14)* | CNP at least three levels below the spinal cord lesion | Dronabinol, max. 20 mg/d x 8w | Dyphenhydramine | BPI |
| **Spasticity with MS and paraplegia** | | | | | | |
| MS | Killestein 2002 | 16 (16)* | 16 (16)* | Primary and secondary progressive MS with severe spasticity | Marinol, 10 mg/d x 4w | Placebo |
|  | Ungerleider 1987 | 13 (13)* | 13 (13)* | MS with significant sclerosis | THC, 2.5-15 mg/d x 5d | Placebo |
|  | Zajicek 2003 | 438 (216) | 438 (216) | MS with problematic spasticity | Marinol, max. 25 mg/d x 14w | Placebo |
|  | van Amerongen 2018 | 24 (12) | 24 (12) | Progressive MS with CNP | Namisol, 9-29 mg/d x 4w | Placebo |
|  | Zajicek 2013 /Ball 2015 | 498 (332) | 498 (332) | Primary or secondary progressive MS | THC, max. 28 mg/d x 36m | Placebo |
| Spinal cord injury | Hagenbach 2007 | 13 (6) | 13 (6) | Paraplegic and tetraplegics | Marinol, 15-60 mg/d x 6w | Placebo |
| **Nausea, vomiting, loss of appetite** | | | | | | |
| Alzheimer | Volicer et al., 1997 | 15 (15)* | Dementia of Alzheimer type with food refusal | Marionol, 5 mg/d x 6w | Placebo | Body weight^P^, intake |
| Amyotrophic lateral sclerosis | Weber et al., 2010 | 27 (27)* | Amyotrophic lateral sclerosis | Dronabinol, 10 mg/d x 2w | Placebo | FAACT |
| Cancer | Brisbois et al., 2011 | 46 (24) | Poor appetite and chemosensory alterations | Marinol, max. 20 mg/d x 18d | Placebo | SLIM^P^, intake, ESAS |
|  | Gilbert et al., 1995 | S162 (31)/S264 (32) | Bone narrow transplant with chemotherapy | Dronabinol 5mg/m2 x1d | Metoclopramide/Prochlorperazine | EPRS, Rhodes INV-2 |
|  | Jatoi et al., 2002 | 311 (152) | Advanced cancer with weight loss and anorexia | Dronabinol, 5 mg/d x 57-80d | Megestrol acetate | NCCTG^N^, FAACT^N^, responders |
|  | Johnson et al., 2010 | 117 (58) | Incurable malignancy | Oromucosal THC, max. 48 sprays (129.6 mg)/d x 2w | Placebo | NRS, EORTC-QLQ-C30 |
|  | Meiri et al., 2007 | 31 (17) | Breast, lung, miscellaneous in chemotherapy | Marinol, max. 20 mg + ondasetron/d x5d | Placebo | VAS~~P~~,responders, presence^P^, episodes |
|  | Neidhardt et al., 1981 | 77 (77)* | Cancer chemotheraputic agent likey to induce intolerable vomiting | THC, max. 100 mg x1d | Haloperidol | Episodes, severity, duration, prevention |
|  | Orr et al., 1981 | 79 (79)* | Repeated vomiting from anticancer agents who falied on standard therapy in chemotherapy | THC, 28 mg/m2 x1d | Placebo | Questionnaire, responders^P^, sign test |
|  | Sallan et al., 1975 | 22 (22)* | Neoplasms refractory to antiemetic therapy in chemotherapy | THC, 15-20 mg/m2 x1d | Placebo | Responders^P^, questionnaires |
|  | Sallan et al., 1980 | 84 (84)* | Neoplasms refractory to antiemetic therapy in chemotherapy | THC, 10 mg/m2 x1d | Prochlorperazine | Responders^P^, rating^P^, preference^P^ |
|  | Strasser et al., 2006 | 148 (100) | Cancer-related anorexia-cachexia syndrome | THC, 5 mg/d x 6w | Placebo | VAS |
|  | Ungerleider et al., 1982 (24) | 214 (214)* | Variety of neoplasms in chemotherapy | THC, 30-50 mg x1d | Prochlorperazine | Rating, intake, appetite, preference |
|  | Lane et al., 1991 | 42 (21) | Breast, lymphoma, colon, lung, miscellaneous in chemotherapy | Marinol, 40 mg x5d | Prochlorperazine | VAS, duration^P^, responders |
| HIV | Beal et al., 1995 | 139 (72) | AIDS | Marinol, 5 mg/d x 6w | Placebo | Body weight, VAS^P^ |
|  | Haney et al., 2005 | 17 (17)* | HIV- positive marijuana smokers with loss of muscle mass | Dronabinol, 10 mg, 20 mg and 30 mg acute | Placebo | VAS^P^, HSQ |
|  | Haney et al., 2007 | 10 (10)* | HIV- positive marijuana smokers | Dronabinol, 20 mg and 40 mg/d x 4d | Placebo | Body weight^P^, food intake^P^, HSQ, VAS |
| Post-operative | Kleine-Brueggeney et al., 2015 | 40 (19) | Laparoscopic/gynecologcal and breast surgery | THC intravenous, max. 10mg x 15min | Placebo | NRS |
| SUDs | Budney et al., 2007 | 22 (22)* | Cannabis users not seeking treatment | Oral THC, 10 mg/d and 30 mg/d x 5d | Placebo | MWC^P^, body weight^P^ |
|  | Lundahl 2015 | 14 (14)* | Cannabis dependent | Marinol, 10 mg and 20 mg acute | Placebo | Appetite VAS |
| **Gastroenterological, neurodegenerative, and other neurological diseases** | | | | | | |
| Amyotrophic lateral sclerosis | Weber 2010 | 27 (27)* | Amyotrophic lateral sclerosis | Dronabinol, 10 mg/d x 2w | Placebo | ALS questionnaire |
| Dystonia | Zadikoff 2011 | 9 (9)* | Cervical dystonia | Marinol, 15 mg/d x 8w | Placebo | TWSTRS, GIC |
| Glaucoma | Tomida et al., 2006 | 6 (6)* | Ocular hypertension, glaucoma | Sublingual THC, 5 mg acute | Placebo | Intraocular pressure^P^ |
| IBS | Klooker et al., 2011 | 10 (10)* | IBS | Marinol, 5 mg and 10 mg acute | Placebo | Visceral sensitivity |
|  | Wong et al., 2011 | 75 (48) | Constipation, diarrhoea or alternating | Dronabinol, 2.5 mg and 5 mg acute | Placebo | PR50^P^,tone, motiliy index^P^,VAS |
| MS | Ball et al., 2015 | 498 (332) | Primary or secondary progressive MS | THC, max. 28 mg/d x 144-168w | Placebo | NRS |
|  | Zajicek 2003 /Freeman 2006 | 438 (216) | MS with urinary symptoms | Marinol, max. 25 mg/d x 14w | Placebo | Diary^P^, questionnaire |
| **Psychiatric disorders** | | | | | | |
| Anorexia Nervosa | Andries 2014/2015 | 24 (24)* | Women with severe, enduring AN | Dronabinol, 5 mg/d x 4w | Placebo | EDI-2^P^, activity^P^ |
|  | Gross 1983 | 11 (11)* | Primary anorexia nervosa | THC, 30 mg/d x 2w | Diazepam | Weight, food intake, GAAQ, SDS, HSCL-90^N^ |
| Anxiety | Bisaga 2015 | 60 (40) | 60 (40) | Opioid dependent seeking treatment | Dronabinol, 30 mg/d x 8d - 5w | Naltrexone |
|  | Budney 2007 | 22 (22)* | 22 (22)* | Cannabis users not seeking treatment | Oral THC, 10 mg/d and 30 mg/d x 5d | Placebo |
|  | De Vries 2017 | 65 (31) | 65 (31) | Chronic pancreatitis and postsurgical pain | Namisol, 8mg/d x 50-52d | Placebo |
|  | Lundahl 2015 | 14 (14)* | Cannabis dependent | Marinol, 10 mg and 20 mg acute | Placebo | Anxiety VAS^P^ |
|  | Malik 2017 | 19 (10) | 19 (10) | CP, Esophageal hypersensitivity | Dronabinol 10 mg/d x 4w | Placebo |
|  | Müller-Vahl 2001/2002 | 12 (12)* | 12 (12)* | Tourette | THC, 5-10 mg acute | Placebo |
|  | Narang 2008 | 30 (30)* | 30 (30)* | Neuropathic, nociceptive, mixed pain | Marinol 10 mg and 20 mg acute | Placebo |
|  | Rabinak 2020 | 61 (32) | 61 (32) | PTSD and trauma-exposed | Dronabinol, 7.5 mg acute | Placebo |
|  | Ungerleider 1982 | 214 (214)* | 214 (214)* | Variety of neoplasms in chemotherapy | THC, 30-50 mg acute | Prochlorperazine |
|  | Weizman 2018 | 15 (15)* | 15 (15)* | Chronic lumbar radicular pain | Sublingual THC, 15.4 + 2.2 mg acute | Placebo |
| Dementia | Van den Elsen 2015 | 22 (22)* | Relevant neurophsyquiatric symptoms, agitation and aggression | Namisol, 1.5 mg and 3mg/d x9d (in 3 days blocks) | Placebo | NPI, CMAI |
|  | Van den Elsen 2015b | 50 (24) | Alzheimer disease, vascular, mixed dementia with relevant neuropsychiatric symptoms | Namisol, 4.5 mg/d x3w | Placebo | NPI, CMAI, CCGIC |
|  | Volicer 1997 | 15 (15)* | Alzheimer type with severe dementia | Marionol, 5 mg/d x 6w | Placebo | CMAI^P^, Lawton^P^ |
| Depression | Ball 2015 | 498 (332) | Primary or secondary progressive MS | THC, max. 28 mg/d x 144-168w | Placebo | NRS |
|  | Bisaga 2015 | 60 (40) | Opioid dependent seeking treatment | Dronabinol, 30 mg x 8d (inpatient) + 5w (outpatient) | Naltrexone | HAM-D 21 |
|  | Budney 2007 | 22 (22)* | Cannabis users not seeking treatment | Oral THC, 10 mg/d and 30 mg/d x 5d | Placebo | MWC^P^ |
|  | De Vries 2017 | 65 (31) | Chronic pancreatitis and postsurgical pain | Namisol, 8mg/d x 50-52d | Placebo | HADS |
|  | Malik 2017 | 19 (10) | CP, Esophageal hypersensitivity | Dronabinol, 10 mg/d x 4w | Placebo | BDI |
|  | Müller-Vahl 2001/2002 | 12 (12)* | Tourette | THC, 5-10 mg acute | Placebo | HDS, SCL-90-R, TSSL |
|  | Narang 2008 | 30 (30)* | Neuropathic, nociceptive, mixed pain | Marinol, 10 mg, 20 mg acute | Placebo | HADS, SPID |
|  | Ungerleider 1982 | 214 (214)* | Variety of neoplasms in chemotherapy | THC, 30-50 mg acute | Prochlorperazine | POMS |
|  | Weber 2010 | 27 (27)* | Amyotrophic lateral sclerosis | Dronabinol, 10 mg/d x 2w | Placebo | HADS |
|  | Zajicek 2003 | 438 (216) | MS with spasticity and CNP | Marinol, max. 25 mg/d x 14w | Placebo | Rating |
| PTSD | Rabinak 2020 | 61 (32) | PTSD and trauma-exposed | Dronabinol, 7.5 mg acute | Placebo | Threat Task^P^ |
| Schizophrenia/ psychosis | D'Souza 2005 | 13 (13)* | Schizophrenia or schizoaffective | Intravenous THC, 2.5 mg and 5 mg acute | Placebo | PANSS^N^, CADSS^N^ |
| Sleep | Berman 2004 | 48 (48)* | CNP | Oromusocsal THC, max. 48 sprays (129.6 mg)/d x 2w | Placebo | BS-11^P^, 4-point score^P^ |
|  | Bisaga 2015 | 60 (40) | Opioid dependent seeking treatment in withdrawal | Dronabinol, 30 mg/d x 8d - 5w | Naltrexone | Rating |
|  | Budney 2007 | 22 (22)* | Cannabis users not seeking treatment | Oral THC, 10 mg/d and 30 mg/d x 5d | Placebo | MWC^P^, SS^P^ |
|  | Carley 2018 | 73 (48) | Obstructive sleep apnea | Dronabinol, 2.5 and 10 mg/d x6w | Placebo | ESS^P^, MWT, latency |
|  | Gross 1983 | 11 (11)* | Primary anorexia nervosa | THC, 30 mg/d x 2w | Diazepam | HSCL-90^N^ |
|  | Haney 2007 | 10 (10)* | HIV- positive marijuana smokers | Dronabinol, 20 mg and 40 mg/d x 4d | Placebo | REM ,VAS |
|  | Johnson 2010 | 117 (58) | Cancer | Oromucosal THC, max. 48 sprays (129.6 mg)/d x 2w | Placebo | NRS, EORTC-QLQ-C30 |
|  | van Amerongen 2018 | 24 (12) | MS with CNP | Namisol, 9-29 mg/d x 4w | Placebo | PSQI |
|  | Weber 2010 | 27 (27)* | Amyotrophic lateral sclerosis | Dronabinol, 10 mg/d x 2w | Placebo | SQD |
|  | Zajicek 2003 | 438 (216) | MS with spasticity and CNP | Marinol, max. 25 mg/d x 14w | Placebo | Rating^P^ |
| SUDs | Bisaga 2015 | 60 (40) | Opioid dependent seeking treatment | Dronabinol, 30 mg/d x 8d - 5w | Naltrexone | SOWS^P^, HAM-D 21, craving, retention |
|  | Budney 2007 | 22 (22)* | Cannabis users not seeking treatment | Oral THC, 10 mg/d and 30 mg/d x 5d | Placebo | MWC^P^, POMS, BSI, MCQ^P^ |
|  | Levin 2011 | 156 (79) | Cannabis dependent seeking treatment | Dronabinol, max. 40 mg/d x 8w | Placebo | Abstinence, retention^P^,TLFB,WDS^P^ |
|  | Lofwall 2016 | 12 (12)* | Opioids and heroin using females | Dronabinol, 5 mg, 10 mg, 20 mg and 30 mg acute | Placebo | SOWS^P^, VAS^P^, OOWS^P^ |
|  | Lundahl 2015 | 14 (14)* | Cannabis dependent | Marinol, 10 mg and 20 mg acute | Placebo | Craving^P^ |
| Tourette | Müller-Vahl 2001/2002 | 12 (12)* | Tourette | THC, 5-10 mg acute | Placebo | GSI, PST, PSDI, TSSL^P^ |
|  | Müller-Vahl 2003/2003b | 24 (12) | Tourette | THC, max. 10 mg/d x 6w | Placebo | MWT-B, BVRT, TAP, TSSL^P^ |

Footnotes: * indicates crossover study; x^P^/x^N^ indicate positive or negative effect on the outcome.

**Supplementary Table 3. Characteristics of included RCTs with nabilone**

| **Disorder** | **Study** | **№ of participants (treated)** | **Sample characteristics** | **Intervention** | **Comparison** | **Outcome or method (s)** |
| --- | --- | --- | --- | --- | --- | --- |
| **Chronic Pain** | | | | | | |
| Alzheimer | Herrmann 2019b | 39 (39)* | Moderate-to-severe AD with agitation and major neurocognitive disorder | Nabilone, max. 2 mg/d x 6w | Placebo | PAINAD |
| Cancer | Côté 2016 | 56 (28) | Radiotherapy for head and neck squamous cell carcinomas | Nabilone, max. 2mg/d x 7w | Placebo | VAS |
|  | Turcott 2018 | 47 (22) | Advanced lung cancer | Nabilone, 1 mg/d x 8w | Placebo | HRQL^P^ |
| Diabetic peripheral NP | Toth 2012 | 26 (13) | CNP with > 30% improvement in pain with nabilone | Nabilone, 1-4 mg/d x 5w | Placebo | NRS^P^, VAS^P^, responders^P^, NPSI^P^, PTSS^P^, PGIC^P^ |
| Fibromyalgia | Skrabek 2008 | 40 (20) | Fibromyalgia | Nabilone, max. 2 mg/d x 4w | Placebo | VAS^P^,tender points, FIQ^P^ |
|  | Ware 2010 | 32 (32)* | Fibromyalgia with chronic insomnia | Nabilone, 0.5-1 mg/d x 2w | Amitriptyline | MPQ |
| Medication oveuse heahache | Pini 2012 | 30 (30)* | Chronic headache with medication overuse | Nabilone, 0.5 mg/d x 8w | Ibuprofen | HI^P^, diary^P^, VAS^P^ |
| MS | Turcotte 2015 | 15 (8) | CNP | Nabilone, max. 2 mg/d x 9w | Placebo | VAS^P^, PGIC^P^ |
| NP | Frank 2008 | 96 (96)* | CNP | Nabilone, max. 2 mg/d x 6w | Dihydrocodeine | VAS^N^, responders |
|  | Pinsger 2006 | 30 (30)* | Chronic therapy-resistant pain with pathologic skeletal and locomotor status system | Nabilone, 0.25-1 mg/d x 4w | Placebo | VAS^P^ |
| Parkinson | Peball 2020 | 38 (19) | Disturbing NMS, responders to open-label nabilone | Nabilone, max. 2mg/d x 4w | Placebo | KPPS, VAS |
| Upper motor neuron syndrome | Wissel 2006 | 13 (13)* | Spasticity-related pain | Nabilone, 1 mg/d x 4w | Placebo | BS-11^P^ |
| **Spasticity with MS and paraplegia** | | | | | | |
| Spinal cord injury | Pooyania 2010 | 12 (12)* | Tetraplegia and paraplegia with spasticity | Nabilone, max. 1 mg/d x 4w | Placebo | VAS, Ashworth^P^, Wartenberf Pendulum, CGI |
| Upper motor neuron syndrome | Wissel 2006 | 13 (13)* | Spasticity | Nabilone, 1 mg/d x 4w | Placebo | Ashworth, RMA, Barthel |
| **Nausea, vomiting, loss of appetite** | | | | | | |
| AD | Herrmann 2019b | 39 (39)* | Moderate-to-severe AD with agitation and major neurocognitive disorder | Nabilone, max. 2 mg/d x 6w | Placebo | MNA-SF^P^, BMI |
| Cancer | Ahmedzai 1983 | 34 (34)* | Lung cancer with chemotherapy | Nabilone, 2mg /d x 3d | Prochlorperazine | Appetite ^P^, nausea/vomiting ^P^, preference^P^ |
|  | Chan 1987 | 40 (40)* | Various pediatric malignancies with chemotherapy | Nabilone, 1-4 mg x1d | Prochlorperazine | Episodes^P^, efficacy^P^, preference^P^ |
|  | Côté 2016 | 56 (28) | Radiotherapy for head and neck squamous cell carcinomas | Nabilone, max. 2mg/d x 7w | Placebo | Appetite, body weigt, questionnaire |
|  | Crawford-Buckman 1986 | 32 (32) * | Ovary adenocarcinoma and germ cell tumors with cisplatin | Nabilone, 3 mg/d x 2d | Metoclopramide | Frequency, VAS, preference, responders^P^, preference |
|  | Dalzell 1986 | 23 (23)* | Various pediatric cancers receiving emetogenic chemotherapy | Nabilone, 1mg-3mg x1d | Domperidone | Frequency^P^,degree^P^, preference^P^ |
|  | Einhorn 1981 | 100 (100)* | Testicular and other cancers with chemotherapy | Nabilone, 8mg/d x 6d | Prochlorperazine | Severity^P^, frequency^P^, preference^P^ |
|  | Johansson 1982 | 26 (26)* | Ovarian carcinoma and other cancers with chemotherapy | Nabilone, 4 mg/d x 2d | Prochlorperazine | Severity^P^, frequency^P^, preference^P^ |
|  | Niederle 1986 | 20 (20)* | Nonseminomatous testicular cancer on cisplatin chemotherapy | Nabilone, 4mg/d x 5d | Alizapride | Nausea^P^, vomiting, intake, preference |
|  | Niiranen 1985 | 32 (32)* | Lung cancer with chemotherapy | Nabilone, 2mg/d x 2d | Prochlorperazine | Severity, frequency^P^, preference^P^ |
|  | Pomeroy 1986 | 38 (19) | Ovary, testis and other tumor types with chemotherapy | Nabilone, 2mg/d x 2d | Domperidone | Severity^P^, frequency^P^ |
|  | Priestman 1987 | 40 (20) | Radiatiation in pelvis, abdomen, thoray, head and neck and others | Nabilone, 2 mg/d x 7d | Metoclopramide | Frequency^P^, severity^P^ |
|  | Turcott 2018 | 47 (22) | Advanced lung cancer with anorexia | Nabilone, 1 mg/d x 8w | Placebo | AC/S, VAS^P^, HRQL, body weight intake^P^ |
|  | Wada 1982 | 114 (114)* | Diverse tumor types with chemotherapy | Nabilone, 4mg acute | Placebo | Severity^P^, frequency^P^, responders^P^, preference^P^ |
|  | Lewis 1994 | 60 (30) | Females with abdominal hysterectomy | Nabilone, 2 mg acute | Metohlopramide | VAS, preference |
|  | Levin 2017 | 340 (172) | Females with different surgeries with 3-4 risk factors for post-operative nausea and vomiting | Nabilone, 0.5 mg acute | Placebo | PONV |
|  | Herrmann 2016 | 15 (15)* | Cannabis users, non-treatment seeking | Nabilone, 6 mg/d x 6d | Placebo | Intake^P^, body weight^P^ |
| **Gastroenterological, neurodegenerative, and other neurological diseases** | | | | | | |
| Chorea Huntington | Curtis 2009 | 44 (44)* | Huntington disease | Nabilone, 1-2 mg/d x 5w | Placebo | Scores^P^, UHDRS, NPI^P^ |
| Distonya | Fox 2002 | 15 (15)* | Generalised or segmental (cranial, axial, braquial) dystonia | Nabilone, 0.03 mg/kg acute | Placebo | Burke, Fahn, Marsden |
| Parkinson | Peball 2020 | 38 (19) | Disturbing NMS, responders to open-label nabilone | Nabilone, max. 2mg/d x 4w | Placebo | MDS-UPDRS-I^P^, NMSS, CGI-I^P^ |
|  | Sieradzan 2001 | 9 (9)* | Idiopathic Parknson disease with stable levodopa-induced dyskinesia 25%-50% of the day | Nabilone, 0.06 mg/kg acute | Placebo | RDDS^P,^ Webster Scale, latency and duration, % dyskinesia |
| **Psychiatric disorders** | | | | | | |
| Anxiety | Fabre 1981 | 20 (10) | Psychoneurotic anxiety | Nabilone, 2 mg/d x 28d | Placebo | SCL-56^P^, HADS^P^, global impression^P^ |
|  | Frank 2008 | 96 (96)* | CNP | Nabilone, max. 2 mg/d x 6w | Dihydrocodeine | HADS |
|  | Glass 1981 | 8 (8)* | Anxiety neurosis, general anxiety | Nabilone, 1 mg, 2 mg and 4 mg or 2 mg, 4 mg and 5 mg acute | Placebo | POMS |
|  | Herrmann 2016 | 15 (15)* | Cannabis users, non-treatment seeking | Nabilone, 6 mg/d x 6d | Placebo | VAS^P^ |
|  | Herrmann 2019a | 33 (33)* | Cannabis+tobacco users, non-treatment seeking | Nabilone, 8 mg/d x 6d | Placebo | VAS |
|  | Hill 2017 | 18 (10) | Cannabis dependent | Nabilone, 2mg/d x 10w | Placebo | BAI |
|  | Peball 2020 | 38 (19) | PD with disturbing NMS, responders to open-label nabilone | Nabilone, max. 2mg/d x 4w | Placebo | HADS |
|  | Pini 2012 | 30 (30)* | Chronic headache with medication overuse | Nabilone, 0.5 mg/d x 8w | Ibuprofen | ZAS |
|  | Skrabek 2008 | 40 (20) | Fibromyalgia | Nabilone, max. 2 mg/d x 4w | Placebo | FIQ^P^ |
|  | Toth 2012 | 26 (13) | Diabethic peripheral CNP with > 30% improvement in pain with nabilone | Nabilone, 1-4 mg/d x 5w | Placebo | HADS^P^ |
| Dementia | Herrmann 2019b | 39 (39)* | Moderate-to-severe AD with agitation and major neurocognitive disorder | Nabilone, max. 2 mg/d x 6w | Placebo | CMAI^P^, sMMSE^P^/SIB^N^, NPI-NH^P,^ CGI^P^/CGIC^P^ |
| Depression | Frank 2008 | 96 (96)* | CNP | Nabilone, max. 2 mg/d x 6w | Dihydrocodeine | HADS |
|  | Glass 1981 | 8 (8)* | Anxiety neurosis, general anxiety | Nabilone, 1 mg, 2 mg and 4 mg or 2 mg, 4 mg and 5 mg acute | Placebo | POMS |
|  | Hill 2017 | 18 (10) | Cannabis dependent | Nabilone, 2mg/d x 10w | Placebo | QIDS |
|  | Pini 2012 | 30 (30)* | Chronic headache with medication overuse | Nabilone, 0.5 mg/d x 8w | Ibuprofen | ZDS |
|  | Peball 2020 | 38 (19) | PD with disturbing NMS, responders to open-label nabilone | Nabilone, max. 2mg/d x 4w | Placebo | HADS |
|  | Skrabek 2008 | 40 (20) | Fibromyalgia | Nabilone, max. 2 mg/d x 4w | Placebo | FIQ |
|  | Toth 2012 | 26 (13) | Diabethic peripheral CNP with > 30% improvement in pain with nabilone | Nabilone, 1-4 mg/d x 5w | Placebo | HADS |
| PTSD | Jetly 2015 | 10 (10)* | Male military with trauma-related nightmares despite treatment | Cesamet, max. 3mg/d x 7w | Placebo | CAPS^P^, CGI-C^P^ |
| Sleep | Frank 2008 | 96 (96)* | CNP | Nabilone, max. 2 mg/d x 6w | Dihydrocodeine | Tick box |
|  | Herrmann 2016 | 15 (15)* | Cannabis users, non-treatment seeking | Nabilone, 6 mg/d x 6d | Placebo | Monitoring System^P^,VAS^P^ |
|  | Herrmann 2019a | 33 (33)* | Cannabis+tobacco users, non-treatment seeking | Nabilone, 8 mg/d x 6d | Placebo | Monitoring System^P^,VAS^P^ |
|  | Jetly 2015 | 10 (10)* | Male military with trauma-related nightmares despite treatment | Cesamet, max. 3 mg/d x 7w | Placebo | Diary |
|  | Peball 2020 | 38 (19) | PD with disturbing NMS, responders to open-label nabilone | Nabilone, max. 2 mg/d x 4w | Placebo | EES |
|  | Skrabek 2008 | 40 (20) | Fibromyalgia | Nabilone, max. 2 mg/d x 4w | Placebo | FIQ |
|  | Toth 2012 | 26 (13) | Diabethic peripheral CNP with > 30% improvement in pain with nabilone | Nabilone, 1-4 mg/d x 5w | Placebo | NRS-11^P^, MOSSS^P^ |
|  | Turcott 2018 | 47 (22) | Advanced lung cancer | Nabilone, 1 mg/d x 8w | Placebo | HRQL^P^ |
|  | Ware 2010 | 32 (32)* | Fibromyalgia with chronic insomnia | Nabilone, 0.5-1 mg/d x 2w | Amitriptyline | ISIS^P^, LSEQ, questionnaire |
| SUDs | Herrmann 2016 | 15 (15)* | Cannabis users, non-treatment seeking | Nabilone, 6 mg/d x 6d | Placebo | VAS^P^, relapse^P^ |
|  | Herrmann 2019a | 33 (33)* | Cannabis+tobacco users, non-treatment seeking | Nabilone, 8 mg/d x 6d | Placebo | Mood VAS^P^, craving VAS, relapse |
|  | Hill 2017 | 18 (10) | Cannabis dependent | Nabilone, 2 mg/d x 10w | Placebo | TLFB, diary, MCQ |

Footnotes: * indicates crossover study; x^P^/x^N^ indicate positive or negative effect on the outcome.

**Supplementary Table 4. Characteristics of included RCTs with CBD**

| **Disorder** | **Study** | **№ of participants (treated)** | **Sample characteristics** | **Intervention** | **Comparison** | **Outcome or method (s)** |
| --- | --- | --- | --- | --- | --- | --- |
| **Chronic Pain** | | | | | | |
| Osteoarthritis | Vela 2021 | 136 (70) | Hand osteoarthristis or psoriatic arthritis | CBD, 20-30 mg/d x 12w | Placebo | VAS, PCS, HAQ-DI |
| **Nausea, vomiting, loss of appetite** | | | | | | |
| Type 2 diabetes | Jadoon 2016 | 27 (13) | Type 2 diabetes | CBD, 200 mg/d x 13w | Placebo | NRS, body weight, CGIC |
| **Gastroenterological, neurodegenerative, and other neurological diseases** | | | | | | |
| Chorea Huntington | Consroe 1991 | 18 (18)* | Chorea Huntington | CBD, 10 mg/kg/d x 6w | Placebo | M and Q, SCL-90-R |
| Epilepsy | Cunha 1980 | 15 (7) | Temporal lobe epilepsy with secondarily feneralized seizures | CBD, 200-300 mg/d x 3-18w | Placebo | Frequency^P^ |
|  | Devinsky 2017 | 120 (61) | Children and young adults with Dravet syndrome | Epidiolex, max. 20 mg/kg/d x14w | Placebo | Frequency^P^, responders, CGICSD^P^ |
|  | Devinsky 2018 | 225 (149) | Lennox- Gastaut syndrome | Epidiolex, 10 mg/kg/d and 20 mg/kg/d x 14w | Placebo | Frequency^P^, responders^P^, CGICSD^P^ |
|  | Miller 2020 | 199 (134) | Children and young adults with Dravet syndrome | Epidiolex, 10 mg/kg/d and 20 mg/kg/d x 14w | Placebo | Frequency^P^, responders^P^, CGICSD^P^ |
|  | Thiele 2018 | 171 (86) | Lennox- Gastaut syndrome | Epidiolex, 20 mg/kg/d x 14w | Placebo | Frequency^P^, responders^P^, CGICSD^P^ |
|  | Thiele 2020 | 224 (148) | Tuberous sclerosis complex | Epidiolex, 25 mg/kg/d and 50 mg/kg/d x 16w | Placebo | Frequency^P^, responders^P^, CGICSD^P^ |
| Parkinson | Chagas 2014 | 21 (14) | PD between stage 1 and stage 3 (Hoehn and Yahr scale) | CBD, 75 mg/d and 300 mg/d x 6w | Placebo | UPDRS, PDQ-39^P^ |
|  | de Almeida 2021 | 36 (20) | REM sleep behavior disorder and Parkinson's disease | CBD, 75-300 mg/d x 12w | Placebo | MDS-UPDRS, UPDRS |
|  | de Faria 2020 | 24 (24)* | PD between stage 1 and stage 2.5 (Hoehn and Yahr scale) | CBD, 300 mg acute | Placebo | Tapping test^P^, accelerometer |
| **Psychiatric disorders** | | | | | | |
| Anxiety | Bergamaschi 2011 | 24 (12) | Social anxiety disorder | CBD, 600 mg acute | Placebo | VAMS^P^, SSPS-N^P^, BSS |
|  | Crippa 2011 | 10 (10)* | Social anxiety disorder | CBD, 400 mg acute | Placebo | VAMS^P^ |
|  | de Almeida 2021 | 36 (20) | REM sleep behavior disorder and Parkinson's disease | CBD, 75-300 mg/d x 12w | Placebo | PAS, Zung |
|  | de Faria 2020 | 24 (24)* | PD between stage 1 and stage 2.5 (Hoehn and Yahr scale) | CBD, 300 mg acute | Placebo | VAMS^P^, SSPS |
|  | Freeman 2020 | 82 (47) | Cannabis use disorder | Oral CBD, 400 and 800 mg x 4w | Placebo | BAI^P^ |
|  | Hurd 2019 | 50 (31) | Heroin use disorder | Epidiolex, 400 and 800 mg/d x 3d | Placebo | VAS^P^ |
|  | Masataka 2019 | 40 (20) | Adolescents with social anxiety disorder | CBD, 300 mg/d x 4w | Placebo | FNE^P^,LSAS^P^ |
|  | Meneses-Gaya 2020 | 31 (14) | Crack-cocaine dependence | Oral CBD, 300 mg /d x 10d | Placebo | BAI |
|  | Morgan 2013 | 24 (12) | Cigarette smokers | Inhaler CBD, 400 microgram/d x 1w | Placebo | MRS |
|  | O'Neill 2020 | 15 (15) | Psychosis | Oral CBD, 600 mg acute | Placebo | STAI-S |
|  | Vela 2021 | 136 (70) | Hand osteoarthristis or psoriatic arthritis | CBD, 20-30 mg/d x 12w | Placebo | HADS |
| Depression | de Almeida 2021 | 36 (20) | REM sleep behavior disorder and Parkinson's disease | CBD, 75-300 mg/d x 12w | Placebo | BDI |
|  | Freeman 2020 | 82 (47) | Cannabis use disorder | Oral CBD, 400 mg and 800 mg x 4w | Placebo | BDI |
|  | Jadoon 2016 | 27 (13) | Type 2 diabetes | CBD, 200 mg/d x 13w | Placebo | BDI-II |
|  | Meneses-Gaya 2020 | 31 (14) | Crack-cocaine dependence | Oral CBD, 300 mg /d x 10d | Placebo | BDI |
|  | Morgan 2013 | 24 (12) | Cigarette smokers | Inhaler CBD, 400 microgram/d x 1w | Placebo | MRS |
|  | Vela 2021 | 136 (70) | Hand osteoarthristis or psoriatic arthritis | CBD, 20-30 mg/d x 12w | Placebo | HADS |
| Schizophrenia/psychosis | Boggs 2018 | 41 (21) | Schizophrenia | Oral CBD, 600 mg/d x6w | Placebo | MCCB-T, PANSS |
|  | Hallak 2010 | 28 (18) | Schizophrenia | Oral CBD, 300 mg and 600 mg acute | Placebo | Stroop color word test |
|  | Leweke 2012 | 42 (21) | Schizophrenia | Oral CBD, 800 mg/d x4w | Amisulpride 800 mg | PANSS, BPRS, respondes |
|  | McGuire 2018 | 88 (43) | Schizophrenia | Oral CBD, 1000 mg/d x6w | Placebo | PANSS^P^, SANS, responders, CGI-I^P^, GAF, BACS |
|  | O'Neill 2020 | 15 (15)* | Psychosis | Oral CBD, 600 mg acute | Placebo | VPA, PANSS |
| Sleep | de Almeida 2021 | 36 (20) | REM sleep behavior disorder and Parkinson's disease | CBD, 75-300 mg/d x 12w | Placebo | PSQi, PDSS, RBDSQ, ESS, Sleep satisfaction^P^, CGI-I, CGI-S |
|  | Devinsky 2017 | 120 (61) | Children and young adults with Dravet syndrome | Epidiolex, max. 20 mg/kg/d x14w | Placebo | NRS, ESS |
|  | Devinsky 2018 | 225 (149) | Lennox- Gastaut syndrome | Epidiolex, 10 mg/kg/d and 20 mg/kg/d x 14w | Placebo | NRS^P^, ESS |
|  | Freeman 2020 | 82 (47) | Cannabis use disorder | Oral CBD, 400 and 800 mg x 4w | Placebo | PSQI^N^ |
|  | McGuire 2018 | 88 (43) | Schizophrenia | Oral CBD, 1000 mg/d x6w | Placebo | CGIC, PGIC |
|  | Meneses-Gaya 2020 | 31 (14) | Crack-cocaine dependence | Oral CBD, 300 mg /d x 10d | Placebo | VAS |
|  | Miller 2020 | 199 (134) | Children and yooung adults with Dravet syndrome | Epidiolex, 10 mg/kg/d and 20 mg/kg/d x 14w | Placebo | NRS, ESS |
|  | Thiele 2018 | 171 (86) | Lennox- Gastaut syndrome | Epidiolex, 20 mg/kg/d x 14w | Placebo | NRS, ESS |
|  | Vela 2021 | 136 (70) | Hand osteoarthristis or psoriatic arthritis | CBD, 20-30 mg/d x 12w | Placebo | PSQI |
| SUDs | Freeman 2020 | 82 (47) | Cannabis use disorder | Oral CBD, 400 and 800 mg x 4w | Placebo | TLFB^P^, use^P^, CWS^P^ |
|  | Haney 2016 | 31 (31)* | Cannabis users | Oral CBD 200 mg, 400 mg and 800 mg acute | Placebo | Self-administration |
|  | Hindocha 2018 | 44 (44)* | Dependent cigarette smokers | Oral CBD, 800 mg acute | Placebo | Visual probe task^P^, MPSS, QSU-B |
|  | Hurd 2019 | 50 (31) | Heroin use disorder | Epidiolex, 400 mg and 800 mg/d x 3d | Placebo | VAS^P^ |
|  | Meneses-Gaya 2020 | 31 (14) | Crack-cocaine dependence | Oral CBD, 300 mg /d x 10d | Placebo | CCQ-Brief, MCCS |
|  | Mongueau-Perusse 2021 | 78 (40) | Cocaine use disorder | Oral CBD, 800 mg/d x 92d | Placebo | VAS, CCQ-Brief, CSSA, TLFB, abstinence |
|  | Morgan 2013 | 24 (12) | Cigarette smokers | Inhaler CBD, 400 microgram/d x 1w | Placebo | Use ^P^, TCQ: 11 |

Footnotes: * indicates crossover study; x^P^/x^N^ indicate positive or negative effect on the outcome.

**Supplementary Table 5. Characteristics of included RCTs with nabiximols**

| **Disorder** | **Study** | **№ of participants (treated)** | **Sample characteristics** | **Intervention** | **Comparison** | **Outcome or method (s)** |
| --- | --- | --- | --- | --- | --- | --- |
| **Chronic Pain** | | | | | | |
| Cancer | Fallon 2017 | S1: 399 (200)/S2: 206 (103) | Advanced cancer with CP | Sativex , max. 10 sprays/d (27 mg THC: 25 mg CBD) x 5w | Placebo | NRS, SGIC^P(S1)^, PGIC, PSQ |
|  | Johnson 2010 | 119 (60) | Incurable malignancy | Sativex ,max. 48 sprays/d (129.6 mg THC: 120 mg CBD) x 2w | Placebo | NRS^P^, BPI-SF, responders |
|  | Lichtman 2018 | 397 (199) | Advanced cancer with CP | Sativex, max. 10 sprays/d (27 mg THC: 25 mg CBD) x 5w | Placebo | NRS, SGIC^P^, PSQ^P^, PGIC^P^ |
|  | Lynch 2014 | 18 (18)* | Chemotherpy-induced NP | Sativex, max. 12 sprays/d (32.4mg THC: 30 mg CBD) x 4w | Placebo | NRS-PI, QST |
|  | Portenoy 2012 | 360 (269) | Cancer with poorly controlled CP | Sativex, max. 4/6/16 sprays/d (43.2 mg THC: 10/40 mg CBD) x 5w | Placebo | NRS^P^, BPI-SF, responders |
| Cerebral palsy | Fairhurst 2020 | 72 (47) | Paedriatic spasticity in cerebral palsy or traumatic brain injury | Sativex, max. 12 sprays/d (32.4 mg THC: 30 mg CBD) x 12w | Placebo | PPP^P^ |
| Diabetic peripheral neuropathy | Selvarajah 2010 | 30 (15) | Chronic painful DPN | Sativex, max. 4 sprays/d (10.8 mg THC: 10 mg CBD) x 12w | Placebo | VAS, NPS, responders, MPQ, SF-36 |
| Motor neuron disease | Riva 2019 | 60 (30) | Amyotrophic lateral sclerosis | Nabiximols, max. 12 sprays/d (32.4 mg THC: 30 mg CBD) x 6w | Placebo | NRS^P^ |
| MS | Collin 2010 | 337 (167) | Advanced MS with severe spasticity | Sativex max. 24 sprays/d (64.8mg THC: 60 mg CBD) x1 4w | Placebo | NRS |
|  | Conte 2009 | 18 (18)* | Secondary progessive MS | Sativex max 48 sprays/d (129.6mg THC: 120 mg CBD) x 3w | Placebo | VAS |
|  | Langford 2013 | 339 (167) | MS | Sativex, max. 12 sprays/d (32.4 mg THC: 30 mg CBD) x 14w | Placebo | NRS, NPS, BPI, responders, SGIC |
|  | Leocani 2015 | 44 (44)* | MS | Sativex, max. 12 sprays/d (32.4 mg THC: 30 mg CBD) x 4w | Placebo | NRS |
|  | Markovà 2019 | 106 (53) | Secondaty progressive and relapsing remiting MS responders to sativex | Sativex, max. 12 sprays/d (32.4 mg THC: 30 mg CBD) x 12w | Placebo | NRS^P^ |
|  | Novotna 2011 | 241 (124) | MS with refractory spasticity resonders to sativex | Sativex, max. 12 sprays/d (32.4 mg THC: 30 mg CBD) x 12w | Placebo | SF-36 |
|  | Rog 2005 | 66 (34) | MS with dysesthetic pain and painful spasms | Sativex ,max. 48 sprays/d (129.6 mg THC: 120 mg CBD) x 5w | Placebo | NRS^P^, NPS^P^, PGIC |
|  | Wade 2004 | 160 (80) | MS | Sativex, max. 120mg THC:120 mg CBD x6w | Placebo | VAS |
| NP | Berman 2004 | 50 (50)* | Brachial plexus avulsion | Sativex max. 48 sprays/d (129.6mg THC: 120 mg CBD) x 2w | Placebo | BS-11^P^, SF-MPQ^P^, PDI^P^, GHQ-12^P^ |
|  | Nurmikko 2007 | 125 (63) | Neuropathic pain with allodynia | Sativex, max. 48 sprays/d (129.6mg THC: 120 mg CBD) x 5w | Placebo | NRS^P^, NPS^P^,PDI^P^, responders^P^, PGIC^P^ |
|  | Serpell 2014 | 246 (128) | Peripheral NP with allodynia | Sativex, max. 24 sparys/d (64.8 mg THC: 60 mg CBD) x14w | Placebo | NRS, NPS, BPI-SF, responders^P^, SGIC^P^ |
| Rheumathoid arthritis | Blake 2006 | 58 (31) | Rheumathoid arthritis | Sativex max. 6 sprays/d (16.2 mg THC: 15mg CBD) x 5w | Placebo | NRS^P^, SF-MPQ^P^ |
| **Spasticity with MS and paraplegia** | | | | | | |
| Cerebral palsy | Fairhurst 2020 | 72 (47) | Paedriatic spasticity in cerebral palsy or traumatic brain injury | Sativex, max. 12 sprays/d (32.4 mg THC: 30 mg CBD) x 12w | Placebo | NRS, MAS, MTS |
| Motor neuron disease | Riva 2019 | 60 (30) | Amyotrophic lateral sclerosis | Nabiximols, max. 12 sprays/d (32.4 mg THC: 30 mg CBD) x 6w | Placebo | MAS^P^, NRS,T10MW, Barthel, PGIC^P^, CGIC, Neurologists's GIC |
| MS | Aragona 2009 | 17 (17)* | MS with spasticity | Sativex, 15 + 4 sprays/d (40.5 mg THC: 37.5 mg CBD) x 3w | Placebo | MSFCS-T25FW, MSFC-9HPT, MSIS-29 |
|  | Collin 2007 | 189 (124) | MS | Sativex, max. 48 sprays/d (129.6mg THC: 120 mg CBD) x 6w | Placebo | NRS^P^, Ashworth, frequency, motricity, responders^P^, PGIC^P^ |
|  | Collin 2010 | 337 (167) | Advanced MS with severe spasticity | Sativex max. 24 sprays/d (64:8mg THC: 60 mg CBD) x1 4w | Placebo | NRS, Ashwoth, NRS, T10MW, Barthel, CGIG, responders |
|  | Conte 2009 | 18 (18)* | Secondary progessive MS | Sativex, max. 48 sprays/d (129.6mg THC: 120 mg CBD) x 3w | Placebo | NRS, Ashworth, H/R ratio |
|  | De Blasiis 2021 | 32 (22) | Relapsing remitting or progressive MS | Sativex, max. 3 sprays/d (8,1 mg THC: 7,5 mg CBD) x 4w | Placebo | MTS, NRS^P,^ 2MWT, MSSS-88, responders^P^, 10MWT^P,^ BBS^P,^ TUG, MSWS-12, MFIS^P,^ optoelectronic^P^ |
|  | Langford 2013 | 339 (167) | MS with CNP | Sativex, max. 12 sprays/d (32.4 mg THC: 30 mg CBD) x 14w | Placebo | NRS |
|  | Leocani 2015 | 44 (44)* | MS | Sativex, max. 12 sprays/d (32.4 mg THC: 30 mg CBD) x 4w | Placebo | MAS^P^, NRS, responders^P^, T10MW, 9HPT |
|  | Markovà 2019 | 106 (53) | Secondaty progressive and relapsing remiting MS responders to sativex (EERW) | Sativex, max. 12 sprays/d (32.4 mg THC: 30 mg CBD) x 12w | Placebo | NRS^P^, MAS^P^, responders^P^, T10MW, Barthel, SGIC, PGIC |
|  | Notcutt 2012 | 36 (18) | Ms with spasticity responders to sativex (EERW) | Sativex, max. 9,2 sprays/d (24.8 mg THC: 23 mg CBD) x 4w | Placebo | Time to failure^P^, NRS, MAS, T10MW, SGIC^P^, CGIC^P^ |
|  | Novotna 2011 | 241 (124) | MS with refractory spasticity resonders to sativex (EERW) | Sativex, max. 12 sprays/d (32.4 mg THC: 30 mg CBD) x 12w | Placebo | MAS, NRS^P^, frequency^P^, responders^P^, T10MW, Barthel^P^, SGIC^P^, CGIC^P^, PGIC^P^ |
|  | Vaney 2004 | 57 (57)* | Primary and secondary progressive, relapsing-remitting MS | Cannabis extract, 12 capsules/d (30 mg THC: 10.8 mg CBD) x 14d | Placebo | Ashworth, RMI, T10MTW, 9HPT |
|  | Wade 2004 | 160 (80) | MS | Sativex, max. 120mg THC:120 mg CBD x6w | Placebo | VAS^P^, MAS, T10MW, Barthel, 9HPT |
| **Nausea, vomiting, loss of appetite** | | | | | | |
| Cancer | Duran 2010 | 16 (7) | Breast and other cancers with or without metastases | Sativex, max. 8 sprays/d (21,6 mg THC: 20 mg CBD) x 4d | Placebo | Responders^P^, duration and severity^P^ |
|  | Grimison 2020 | 81 (81)* | Any malignancy of any stage receiving chemotherapy of moderate or high emetogenic risk | Oral THC:CBD, max. (THC 100 mg: CBD 100 mg)/d x 5d | Placebo | Responders^P^, frequency^P^, intensity^P^, preference^P^ |
|  | Johnson 2010 | 119 (60) | Incurable malignancy | Sativex ,max. 48 sprays/d (129.6 mg THC: 120 mg CBD) x 2w | Placebo | NRS^P^, EORTC-QLQ-C30^N^ |
|  | Strasser 2006 | 143 (95) | Cancer-related anorexia-cachexia syndrome | THC 2.5 mg: CBD 1 mg x 2/d (THC 5 mg: CBD 2 mg) x 6w | Placebo | VAS |
| Motor neuron disease | Riva 2019 | 60 (30) | Amyotrophic lateral sclerosis | Nabiximols, max. 12 sprays/d (32.4 mg THC: 30 mg CBD) x 6w | Placebo | Body weight |
| SUDs | Allsop 2014 | 51 (27) | Cannabis dependent | Sativex, max. 24 sprays/d (64.8mg THC: 60 mg CBD) x 6d | Placebo | CWS |
|  | Trigo 2016 | 16 (16)* | Cannabis dependent during withdrawal | Sativex, fixed and max. 40 sprays/d (108 mg THC: 100 mg CBD), 5d x 4 (ABACADAE design) | Placebo | MWC, body weight^P^ |
|  | Trigo 2018 | 40 (20) | Cannabis dependent | Sativex, max. 42 sprays/d (113.4 mg THC: 105 mg CBD) x 12w | Placebo | MWC, body weight |
| **Gastroenterological, neurodegenerative, and other neurological diseases** | | | | | | |
| Amyotrophic lateral sclerosis | Riva 2019 | 60 (30) | Amyotrophic lateral sclerosis | Nabiximols, max. 12 sprays/d (32.4 mg THC: 30 mg CBD) x 6w | Placebo | ALSFRS |
| Chorea Hunington | López-Sendón 2016 | 26 (26)* | Huntington | Sativex, max. 12 sprays/d (32.4 mg THC: 30 mg CBD) x 12w | Placebo | UHDRS |
| Dystonia | López-Sendón 2016 | 26 (26)* | Huntington | Sativex, max. 12 sprays/d (32.4 mg THC: 30 mg CBD) x 12w | Placebo | UHDRS |
| Glaucoma | Tomida 2006 | 6 (6)* | Ocular hypertension, glaucoma | Sublingual CBD: THC (1:21). 20, 40 mg acute | Placebo | Intraocular pressure^N^ |
| MS | Collin 2010 | 337 (167) | Advanced MS with severe spasticity | Sativex max. 24 sprays/d (64.8mg THC: 60 mg CBD) x1 4w | Placebo | NRS |
|  | Kavia 2010 | 135 (67) | MS with overactive bladder | Sativex, max. 48 sprays/d (129.6mg THC: 120 mg CBD)/d x 10w | Placebo | Incontinence, voids^P^, nocturnia^P^, OBC^P^, PGIC^P^ |
|  | Langford 2013 | 339 (167) | MS with CNP | Sativex, max. 12 sprays/d (32.4 mg THC: 30 mg CBD) x 14w | Placebo | NRS |
|  | Vaney 2004 | 57 (57)* | Primary and secondary progressive, relapsing-remitting MS | Cannabis extract, 12 capsules/d (30 mg THC: 10.8 mg CBD) x 14d | Placebo | Diary |
|  | Wade 2004 | 160 (80) | MS | Sativex, max. 120mg THC:120 mg CBD x6w | Placebo | VAS, ADL questionnaire |
| **Psychiatric disorders** | | | | | | |
| ADHD | Cooper 2017 | 30 (15) | ADHD | Sativex, max. 14 sprays/d (37.8 mg THC: 35 mg CBD) x 6w | Placebo | QbTest^P^, CAARS^P^,WRAADS, SART |
| Anxiety | Allsop 2014 | 51 (27) | Cannabis dependent | Sativex, max. 24 sprays/d (64.8mg THC: 60 mg CBD) x 6d | Placebo | CWS^P^ |
|  | Aragona 2009 | 17 (17)* | MS with spasticity | Sativex, 15 + 4 sprays/d (40.5 mg THC: 37.5 mg CBD) x 3w | Placebo | SAS, SCL-90-R |
|  | López-Sendón 2016 | 26 (26)* | HD | Sativex, max. 12 sprays/d (32.4 mg THC: 30 mg CBD) x 12w | Placebo | HADS |
|  | Rog 2005 | 66 (34) | MS with dysesthetic pain and painful spasms | Sativex ,max. 48 sprays/d (129.6 mg THC: 120 mg CBD) x 5w | Placebo | HADS |
|  | Trigo 2016 | 16 (16)* | Cannabis dependent during withdrawal | Sativex, fixed and max. 40 sprays/d (108 mg THC: 100 mg CBD), 5d x 4 (ABACADAE design) | Placebo | MWC |
|  | Trigo 2018 | 40 (20) | Cannabis dependent | Sativex, max. 42 sprays/d (113.4 mg THC: 105 mg CBD) x 12w | Placebo | HAM-A^P^ |
| Depression | Allsop 2014 | 51 (27) | Cannabis dependent | Sativex, max. 24 sprays/d (64.8mg THC: 60 mg CBD) x 6d | Placebo | CWS |
|  | Aragona 2009 | 17 (17)* | MS with spasticity | Sativex, 15 + 4 sprays/d (40.5 mg THC: 37.5 mg CBD) x 3w | Placebo | SCL-90-R |
|  | López-Sendón 2016 | 26 (26)* | HD | Sativex, max. 12 sprays/d (32.4 mg THC: 30 mg CBD) x 12w | Placebo | HADS |
|  | Novotna 2011 | 241 (124) | MS with refractory spasticity resonders to sativex (EERW) | Sativex, max. 12 sprays/d (32.4 mg THC: 30 mg CBD) x 12w | Placebo | BDI-II |
|  | Rog 2005 | 66 (34) | MS with dysesthetic pain and painful spasms | Sativex ,max. 48 sprays/d (129.6 mg THC: 120 mg CBD) x 5w | Placebo | HADS |
|  | Trigo 2016 | 16 (16)* | Cannabis dependent during withdrawal | Sativex, fixed and max. 40 sprays/d (108 mg THC: 100 mg CBD), 5d x 4 (ABACADAE design) | Placebo | MWC, HAM-D |
|  | Trigo 2018 | 40 (20) | Cannabis dependent | Sativex, max. 42 sprays/d (113.4 mg THC: 105 mg CBD) x 12w | Placebo | HAM-D, BDI |
|  | Wade 2004 | 160 (80) | MS | Sativex, max. 120mg THC:120 mg CBD x6w | Placebo | BDI |
| Sleep | Allsop 2014 | 51 (27) | Cannabis dependent | Sativex, max. 24 sprays/d (64.8mg THC: 60 mg CBD) x 6d | Placebo | CWS |
|  | Berman 2004 | 50 (50)* | CNP | Sativex max. 48 sprays/d (129.6mg THC: 120 mg CBD) x 2w | Placebo | BS-11^P^, 4-point score^P^ |
|  | Blake 2006 | 58 (31) | Rheumathoid arthritis | Sativex max. 6 sprays/d (16.2 mg THC: 15mg CBD) x 5w | Placebo | NRS^P^ |
|  | Collin 2010 | 337 (167) | Advanced MS with severe spasticity | Sativex max. 24 sprays/d (64:8mg THC: 60 mg CBD) x1 4w | Placebo | NRS |
|  | Fairhurst 2020 | 72 (47) | Paedriatic spasticity in cerebral palsy or traumatic brain injury | Sativex, max. 12 sprays/d (32.4 mg THC: 30 mg CBD) x 12w | Placebo | NRS |
|  | Fallon 2017 | S1: 399 (200)/S2: 206 (103) | Advanced cancer with CP | Sativex , max. 10 sprays/d (27 mg THC: 25 mg CBD) x 5w | Placebo | NRS |
|  | Johnson 2010 | 119 (60) | Cancer | Sativex ,max. 48 sprays/d (129.6 mg THC: 120 mg CBD) x 2w | Placebo | NRS, EORTC-QLQ-C30 |
|  | Langford 2013 | 339 (167) | MS with CNP | Sativex, max. 12 sprays/d (32.4 mg THC: 30 mg CBD) x 14w | Placebo | NRS |
|  | Leocani 2015 | 44 (44)* | MS | Sativex, max. 12 sprays/d (32.4 mg THC: 30 mg CBD) x 4w | Placebo | NRS |
|  | Lichtman 2018 | 397 (199) | Advanced cancer with CP | Sativex, max. 10 sprays/d (27 mg THC: 25 mg CBD) x 5w | Placebo | NRS^P^ |
|  | Markovà 2019 | 106 (53) | Secondaty progressive and relapsing remiting MS responders to sativex | Sativex, max. 12 sprays/d (32.4 mg THC: 30 mg CBD) x 12w | Placebo | NRS^P^ |
|  | Notcutt 2012 | 36 (18) | Ms with spasticity responders to sativex | Sativex max 9,2 sprays/d (24.8 mg THC: 23 mg CBD) x 4w | Placebo | NRS |
|  | Novotna 2011 | 241 (124) | MS with refractory spasticity resonders to sativex | Sativex, max. 12 sprays/d (32.4 mg THC: 30 mg CBD) x 12w | Placebo | NRS^P^ |
|  | Nurmikko 2007 | 125 (63) | Neuropathic pain with allodynia | Sativex, max. 48 sprays/d (129.6mg THC: 120 mg CBD) x 5w | Placebo | NRS^P^ |
|  | Portenoy 2012 | 360 (269) | Cancer with poorly controlled CP | Sativex, max. 4/6/16 sprays/d (43.2 mg THC: 10/40 mg CBD) x 5w | Placebo | NRS^P^ |
|  | Riva 2019 | 60 (30) | ALS | Nabiximols, max. 12 sprays/d (32.4 mg THC: 30 mg CBD) x 6w | Placebo | NRS |
|  | Rog 2005 | 66 (34) | MS with dysesthetic pain and painful spasms | Sativex ,max. 48 sprays/d (129.6 mg THC: 120 mg CBD) x 5w | Placebo | NRS^P^ |
|  | Serpell 2014 | 246 (128) | Peripheral NP with allodynia | Sativex, max. 24 sparys/d (64.8 mg THC: 60 mg CBD) x14w | Placebo | NRS^P^ |
|  | Trigo 2016 | 16 (16)* | Cannabis dependent | Sativex, fixed and max. 40 sprays/d (108 mg THC: 100 mg CBD), 5d x 4 (ABACADAE design) | Placebo | MWC, SMHSQ |
|  | Trigo 2018 | 40 (20) | Cannabis dependent | Sativex, max. 42 sprays/d (113.4 mg THC: 105 mg CBD) x 12w | Placebo | SMHSQ |
|  | Vaney 2004 | 57 (57)* | Primary and secondary progressive, relapsing-remitting MS | Cannabis extract, 12 capsules/d (30 mg THC: 10.8 mg CBD) x 14d | Placebo | Diary |
|  | Wade 2004 | 160 (80) | MS | Sativex, max. 120mg THC:120 mg CBD x6w | Placebo | VAS^P^ |
| SUDs | Allsop 2014 | 51 (27) | Cannabis dependent | Sativex, max. 24 sprays/d (64.8mg THC: 60 mg CBD) x 6d | Placebo | CWS^P^, retention^P^ |
|  | Lintzeris 2019 | 137 (64) | Cannabis dependent | Sativex, max. 32 sprays/d (86.4 mg THC: 80 mg CBD) x 12w | Placebo | Use^P^, TLFB, abstinence, CWS, MCQ |
|  | Trigo 2016 | 16 (16)* | Cannabis dependent | Sativex, fixed and max. 40 sprays/d (108 mg THC: 100 mg CBD), 5d x 4 (ABACADAE design) | Placebo | CWS^P^, MWC^P^, MCQ |
|  | Trigo 2018 | 40 (20) | Cannabis dependent | Sativex, max. 42 sprays/d (113.4 mg THC: 105 mg CBD) x 12w | Placebo | Use, MWC, rate, MCQ |

Footnotes: * indicates crossover study; x^P^/x^N^ indicate positive or negative effect on the outcome. S (study).
