# Supplementary material for: Medical cannabinoids: a pharmacology-based systematic review and meta-analysis for all relevant medical indications
Source: BMC Med. 2022 Aug 19;20:259. doi: 10.1186/s12916-022-02459-1 (PMC9389720; doi:10.1186/s12916-022-02459-1)
Supplement: Supplementary file 3 — Additional file 3. Risk of bias assessments of included studies. [file 12916_2022_2459_MOESM3_ESM.docx]

**Risk of bias assessment**

**Supplementary Table 6. Risk of bias assessment of included studies**

| **Ahmedzai 1983** | | |
| --- | --- | --- |
| **Bias** | **Authors’**  **judgement** | **Support for judgement** |
| Random sequence generation (selection bias) | Unclear | No information |
| Allocation concealment (selection bias) | Unclear | No information |
| Blinding of participants and personnel (performance bias) | Unclear | No information |
| Blinding of outcome assessment (detection bias) | Unclear | No information |
| Incomplete outcome data (attrition bias) | High | Droupouts excluded, carry over test not performed |
| Selective reporting (reporting bias) | Unclear | All outcomes reported but not for each period |
| Other bias | Unclear | Small sample size |
| **Allsop 2014** |  |  |
| **Bias** | **Authors’**  **judgement** | **Support for judgement** |
| Random sequence generation (selection bias) | Low | Random block sizes in Stata |
| Allocation concealment (selection bias) | Low | Matched placebo developed by GW Pharmaceuticals |
| Blinding of participants and personnel (performance bias) | Low | Patients, investigators and outcome assessor were blind to treatment allocation |
| Blinding of outcome assessment (detection bias) | Low | Blind to treatment allocation until all research procedures were complete |
| Incomplete outcome data (attrition bias) | Low | All patients evaluated. 14,4% missing data, assessed with Little test |
| Selective reporting (reporting bias) | Low | All outcomes reported |
| Other bias | Unclear | Patients were administered sleep medications |
| **Andries 2014/2015** | | |
| **Bias** | **Authors’**  **judgement** | **Support for judgement** |
| Random sequence generation (selection bias) | Low | Computer generated |
| Allocation concealment (selection bias) | Low | The allocation was concealed from the enrolling and assessing researches in opaque sealed and stapled envelopes |
| Blinding of participants and personnel (performance bias) | Low | Blinding was strictly maintained by emphasizing to both intervention staff and participants. The personnel were also blind |
| Blinding of outcome assessment (detection bias) | Low | Blinding was strictly maintained by emphasizing to both intervention staff and participants. The personnel were also blind |
| Incomplete outcome data (attrition bias) | Low | No dropouts, carry over test performed./ Missing data due to technical reasons, replaced with the LOCF |
| Selective reporting (reporting bias) | Low | All outcomes reported |
| Other bias | Unclear | Small sample size |
| **Aragona 2009** |  |  |
| **Bias** | **Authors’**  **judgement** | **Support for judgement** |
| Random sequence generation (selection bias) | Low | Independent statistician-generated randomization code |
| Allocation concealment (selection bias) | Unclear | No information |
| Blinding of participants and personnel (performance bias) | Low | Identical appearance, smell and taste |
| Blinding of outcome assessment (detection bias) | Unclear | No information |
| Incomplete outcome data (attrition bias) | Unclear | No statistical period effect |
| Selective reporting (reporting bias) | Low | All outcomes reported |
| Other bias | Unclear | Small sample size |
| **Beal 1995** |  |  |
| **Bias** | **Authors’**  **judgement** | **Support for judgement** |
| Random sequence generation (selection bias) | Unclear | No information |
| Allocation concealment (selection bias) | Unclear | No information |
| Blinding of participants and personnel (performance bias) | Low | Identical capsules |
| Blinding of outcome assessment (detection bias) | Unclear | No information |
| Incomplete outcome data (attrition bias) | Unclear | Analysis of both evaluable and non evaluable patients |
| Selective reporting (reporting bias) | Low | All outcomes reported |
| Other bias | Low |  |
| **Bergamaschi 2011** |  |  |
| **Bias** | **Authors’**  **judgement** | **Support for judgement** |
| Random sequence generation (selection bias) | Low | According to gender, age, years, socioeconomic status and social phobia |
| Allocation concealment (selection bias) | Low | The first participant had his treatment blindy chosed between the two options, the next participant (whose characteristics were matched) had his treatment drawn from the remaining option |
| Blinding of participants and personnel (performance bias) | Low | Identical gelatine capsules |
| Blinding of outcome assessment (detection bias) | Unclear | No information |
| Incomplete outcome data (attrition bias) | Unclear | No information on missing data handling |
| Selective reporting (reporting bias) | Low | All outcomes reported |
| Other bias | Unclear | Small sample size |
| **Berman 2004** |  |  |
| **Bias** | **Authors’**  **judgement** | **Support for judgement** |
| Random sequence generation (selection bias) | Low | Computer generated |
| Allocation concealment (selection bias) | Low | Sealed code break envelopes containing information on the treatment sequence |
| Blinding of participants and personnel (performance bias) | Low | Treatment sequence was blinded |
| Blinding of outcome assessment (detection bias) | Low | Blinding was maintained throughout the study |
| Incomplete outcome data (attrition bias) | Low | All patients were analysed, period analysis |
| Selective reporting (reporting bias) | Low | All outcomes reported |
| Other bias | Unclear | Small sample size |
| **Bisaga 2015** |  |  |
| **Bias** | **Authors’**  **judgement** | **Support for judgement** |
| Random sequence generation (selection bias) | Low | Stratification on opioid use and age |
| Allocation concealment (selection bias) | Unclear | No information |
| Blinding of participants and personnel (performance bias) | Unclear | No information |
| Blinding of outcome assessment (detection bias) | Unclear | No information |
| Incomplete outcome data (attrition bias) | Low | ITT |
| Selective reporting (reporting bias) | Unclear | Treatment and study week interactions are not shown in some cases |
| Other bias | Unclear | Laboratory study, short term inpatient |
| **Blake 2006** |  |  |
| **Bias** | **Authors’**  **judgement** | **Support for judgement** |
| Random sequence generation (selection bias) | Low | Randomized treatment allocation using permutted blocks of four |
| Allocation concealment (selection bias) | Unclear | No information |
| Blinding of participants and personnel (performance bias) | Unclear | No information |
| Blinding of outcome assessment (detection bias) | Unclear | No information |
| Incomplete outcome data (attrition bias) | Unclear | Dropouts not balanced, no information about missing data handling |
| Selective reporting (reporting bias) | Low | All outcomes reported |
| Other bias | Unclear | Short communication |
| **Boggs 2018** |  |  |
| **Bias** | **Authors’**  **judgement** | **Support for judgement** |
| Random sequence generation (selection bias) | Unclear | No information |
| Allocation concealment (selection bias) | Unclear | No information |
| Blinding of participants and personnel (performance bias) | Unclear | No information |
| Blinding of outcome assessment (detection bias) | Unclear | No information |
| Incomplete outcome data (attrition bias) | Unclear | Only completers, no information about missing data |
| Selective reporting (reporting bias) | Low | All outcomes reported |
| Other bias | Unclear | Small sample size |
| **Brisbois 2011** |  |  |
| **Bias** | **Authors’**  **judgement** | **Support for judgement** |
| Random sequence generation (selection bias) | Unclear | Computer generated |
| Allocation concealment (selection bias) | Low | By a third party pharmacist |
| Blinding of participants and personnel (performance bias) | Unclear | No information |
| Blinding of outcome assessment (detection bias) | Unclear | No information |
| Incomplete outcome data (attrition bias) | Unclear | PP analysis |
| Selective reporting (reporting bias) | Unclear | Nausea not reported |
| Other bias | Unclear | Pilot study, small sample size |
| **Budney 2007** |  |  |
| **Bias** | **Authors’**  **judgement** | **Support for judgement** |
| Random sequence generation (selection bias) | Unclear | No information |
| Allocation concealment (selection bias) | Low | Capsules in unidose packaging |
| Blinding of participants and personnel (performance bias) | Unclear | No information |
| Blinding of outcome assessment (detection bias) | Unclear | No information |
| Incomplete outcome data (attrition bias) | Unclear | Only the data from patients showing withdrawal were analysed |
| Selective reporting (reporting bias) | Unclear | Many outcomes listed are not completely reported (WDS, POMS) |
| Other bias | Unclear | Pilot study, small sample size |
| **Carley 2018** |  |  |
| **Bias** | **Authors’**  **judgement** | **Support for judgement** |
| Random sequence generation (selection bias) | Low | Prepared and maintained by the investigational pharmacy |
| Allocation concealment (selection bias) | Low | Coded blister cards labelled with the data for each pill |
| Blinding of participants and personnel (performance bias) | Low | Blinded study agent in white capsules |
| Blinding of outcome assessment (detection bias) | Low | A single board-certified polysomnigrapher, blinded to participant and treatment, score all PSG and MWT studies |
| Incomplete outcome data (attrition bias) | Low | ITT; direct likelihood estimation method |
| Selective reporting (reporting bias) | Low | All outcomes reported |
| Other bias | Low |  |
| **Chagas 2014** |  |  |
| **Bias** | **Authors’**  **judgement** | **Support for judgement** |
| Random sequence generation (selection bias) | Low | Patients matched according to age, gender, PD duration and total UPDRS score |
| Allocation concealment (selection bias) | Unclear | No information |
| Blinding of participants and personnel (performance bias) | Low | Identical capsules |
| Blinding of outcome assessment (detection bias) | Unclear | No information |
| Incomplete outcome data (attrition bias) | Low | No dropouts |
| Selective reporting (reporting bias) | Low | All outcomes reported |
| Other bias | Unclear | Small sample size |
| **Chan 1987** |  |  |
| **Bias** | **Authors’**  **judgement** | **Support for judgement** |
| Random sequence generation (selection bias) | Unclear | Not fully described: sequence randomly assigned |
| Allocation concealment (selection bias) | Low | Neither the medical personnel nor the patients were aware of the order |
| Blinding of participants and personnel (performance bias) | Low | Identical capsules in appearance |
| Blinding of outcome assessment (detection bias) | Low | Outcomes were assessed by nurses and parents |
| Incomplete outcome data (attrition bias) | Unclear | No information about missing data handling |
| Selective reporting (reporting bias) | High | Data from the 10 dropouts are not included, which had major adverse effects |
| Other bias | Unclear | There was a change in the dosage schedule |
| **Collin 2007** |  |  |
| **Bias** | **Authors’**  **judgement** | **Support for judgement** |
| Random sequence generation (selection bias) | Unclear | No information |
| Allocation concealment (selection bias) | Unclear | No information |
| Blinding of participants and personnel (performance bias) | Low | The control preparation was identically flavoured incipient to reduce the risk of unblinding |
| Blinding of outcome assessment (detection bias) | Low | The control preparation was identically flavoured incipient to reduce the risk of unblinding |
| Incomplete outcome data (attrition bias) | Unclear | ITT, no information about missing data handling |
| Selective reporting (reporting bias) | Low | All outcomes reported |
| Other bias | Low |  |
| **Collin 2010** |  |  |
| **Bias** | **Authors’**  **judgement** | **Support for judgement** |
| Random sequence generation (selection bias) | Unclear | No information |
| Allocation concealment (selection bias) | Unclear | No information |
| Blinding of participants and personnel (performance bias) | Low | Each actuation of placebo delivered excipients plus colourants |
| Blinding of outcome assessment (detection bias) | Low | Each actuation of placebo delivered excipients plus colourants |
| Incomplete outcome data (attrition bias) | Unclear | ITT, LOCF |
| Selective reporting (reporting bias) | Low | All outcomes reported |
| Other bias | Low |  |
| **Consroe 1991** |  |  |
| **Bias** | **Authors’**  **judgement** | **Support for judgement** |
| Random sequence generation (selection bias) | Unclear | No information |
| Allocation concealment (selection bias) | Unclear | No information |
| Blinding of participants and personnel (performance bias) | Low | Identically appearing capsules |
| Blinding of outcome assessment (detection bias) | Low | Videotapes were evaluated blindly and independently by 2 neurologists |
| Incomplete outcome data (attrition bias) | Unclear | Period analysis, but no information about missing data |
| Selective reporting (reporting bias) | Low | Statistics from both periods shown, all outcomes reported |
| Other bias | Unclear | Small sample size |
| **Conte 2009** |  |  |
| **Bias** | **Authors’**  **judgement** | **Support for judgement** |
| Random sequence generation (selection bias) | Low | Computer generated |
| Allocation concealment (selection bias) | Unclear | No information |
| Blinding of participants and personnel (performance bias) | Unclear | No information |
| Blinding of outcome assessment (detection bias) | Unclear | No information |
| Incomplete outcome data (attrition bias) | Unclear | No carry over or period analysis |
| Selective reporting (reporting bias) | High | Data from Ashworth, NRS not reported. Data from pain VAS baseline not reported. |
| Other bias | Unclear | Small sample size |
| **Cooper 2017** |  |  |
| **Bias** | **Authors’**  **judgement** | **Support for judgement** |
| Random sequence generation (selection bias) | Low | Independent statistician |
| Allocation concealment (selection bias) | Low | The allocation was concealed in opaque sealed envelopes |
| Blinding of participants and personnel (performance bias) | Low | Treatments were identical in appearance and taste |
| Blinding of outcome assessment (detection bias) | Low | Both investigators and participants were blind to treatment allocation |
| Incomplete outcome data (attrition bias) | Low | ITT, sensitivity analysis with Missing at Random and Missing Not at Random |
| Selective reporting (reporting bias) | Low | All outcomes reported |
| Other bias | Unclear | Small sample size, pilot study |
| **Côté 2016** |  |  |
| **Bias** | **Authors’**  **judgement** | **Support for judgement** |
| Random sequence generation (selection bias) | Low | Blocks of 8, with an allocation ratio of 1:1 |
| Allocation concealment (selection bias) | Low | Both drugs looked identical |
| Blinding of participants and personnel (performance bias) | Low | The physicians, nurse, and subjects were blinded: The hospital pharmacist was the only one who knew patients’grouping. |
| Blinding of outcome assessment (detection bias) | Low | For the entire trial, physicians, nurse, and subjects were blinded |
| Incomplete outcome data (attrition bias) | Unclear | More dropouts in the placebo, most of them were receiving radiochemotherapy |
| Selective reporting (reporting bias) | Low | All data reported |
| Other bias |  |  |
| **Crawford-Buckman 1986** |  |  |
| **Bias** | **Authors’**  **judgement** | **Support for judgement** |
| Random sequence generation (selection bias) | Unclear | No information |
| Allocation concealment (selection bias) | Unclear | No information |
| Blinding of participants and personnel (performance bias) | Unclear | No information |
| Blinding of outcome assessment (detection bias) | Unclear | No information |
| Incomplete outcome data (attrition bias) | High | High dropout, no information about missing data handling |
| Selective reporting (reporting bias) | Low | All outcomes reported |
| Other bias | Unclear | Small sample size |
| **Crippa 2011** |  |  |
| **Bias** | **Authors’**  **judgement** | **Support for judgement** |
| Random sequence generation (selection bias) | Unclear | No information |
| Allocation concealment (selection bias) | Unclear | No information |
| Blinding of participants and personnel (performance bias) | Low | Identical gelatin capsules |
| Blinding of outcome assessment (detection bias) | Low | Identical gelatin capsules |
| Incomplete outcome data (attrition bias) | Unclear | No carry over or period statistics |
| Selective reporting (reporting bias) | Low | All outcomes reported |
| Other bias | Unclear | Small sample size |
| **Cunha 1980** |  |  |
| **Bias** | **Authors’**  **judgement** | **Support for judgement** |
| Random sequence generation (selection bias) | Unclear | No information |
| Allocation concealment (selection bias) | Unclear | No information |
| Blinding of participants and personnel (performance bias) | Unclear | No information |
| Blinding of outcome assessment (detection bias) | Unclear | No information |
| Incomplete outcome data (attrition bias) | High | No iITT, no period or carry over analysis, no information about outcome time |
| Selective reporting (reporting bias) | Low | All outcomes reported |
| Other bias | Unclear | Small sample size |
| **Curtis 2009** |  |  |
| **Bias** | **Authors’**  **judgement** | **Support for judgement** |
| Random sequence generation (selection bias) | Low | Central randomization service |
| Allocation concealment (selection bias) | Low | Patients and assessor were blind to treatment allocation |
| Blinding of participants and personnel (performance bias) | Low | Supplied nabilone and identical placebo capsules |
| Blinding of outcome assessment (detection bias) | Low | All assessments were made by a single assessor blind to treatment order |
| Incomplete outcome data (attrition bias) | Unclear | No information about missing data or dropouts handling |
| Selective reporting (reporting bias) | Low | All data reported, crossover analysis |
| Other bias | Unclear | Pilot study, small sample size |
| **D’Souza 2005** |  |  |
| **Bias** | **Author’s judgement** | **Support for judgement** |
| Random sequence generation (selection bias) | Unclear | No information |
| Allocation concealment (selection bias) | Unclear | No information |
| Blinding of participants and personnel (performance bias) | Unclear | No information |
| Blinding of outcome assessment (detection bias) | Unclear | No information |
| Incomplete outcome data (attrition bias) | Unclear | Only those retrospective data that conflict with data collected at other time points were reported |
| Selective reporting (reporting bias) | Low | All outcomes reported |
| Other bias | Unclear | Small sample size, study was terminated |
| **Dalzell 1986** |  |  |
| **Bias** | **Author’s judgement** | **Support for judgement** |
| Random sequence generation (selection bias) | Low | Patients stratified according to weight |
| Allocation concealment (selection bias) | Unclear | No information |
| Blinding of participants and personnel (performance bias) | Low | Identical looking capsules or white powders |
| Blinding of outcome assessment (detection bias) | Unclear | No information |
| Incomplete outcome data (attrition bias) | High | Most dropouts with nabilones, no period analysis |
| Selective reporting (reporting bias) | Low | All outcomes reported |
| Other bias | Unclear | Small sample size |
| **De Almeida 2021** |  |  |
| **Bias** | **Author’s judgement** | **Support for judgement** |
| Random sequence generation (selection bias) | Low | Computer blocked sequence (1:1) |
| Allocation concealment (selection bias) | Low | Capsules stored in cartons containing the patients initials |
| Blinding of participants and personnel (performance bias) | Low | Identical capsules |
| Blinding of outcome assessment (detection bias) | Unclear | No information |
| Incomplete outcome data (attrition bias) | Low | ITT, no missing data |
| Selective reporting (reporting bias) | Low | All outcomes reported |
| Other bias | Unclear | Small sample size |
| **De Blasiis 2021** |  |  |
| **Bias** | **Author’s judgement** | **Support for judgement** |
| Random sequence generation (selection bias) | Low | Randomized blocks methodology |
| Allocation concealment (selection bias) | Unclear | No information |
| Blinding of participants and personnel (performance bias) | Unclear | No information |
| Blinding of outcome assessment (detection bias) | Unclear | No information |
| Incomplete outcome data (attrition bias) | Unclear | No size calculation |
| Selective reporting (reporting bias) | Unclear | MTS scores are not shown |
| Other bias | Unclear | Small sample size, pilot study |
| **De Faria 2020** |  |  |
| **Bias** | **Author’s judgement** | **Support for judgement** |
| Random sequence generation (selection bias) | Unclear | No information |
| Allocation concealment (selection bias) | Unclear | No information |
| Blinding of participants and personnel (performance bias) | Low | Identical capsules |
| Blinding of outcome assessment (detection bias) | Low | Identical capsules |
| Incomplete outcome data (attrition bias) | Low | Phase and ordr analysis done |
| Selective reporting (reporting bias) | Low | All outcomes reported |
| Other bias | Unclear | Small sample size |
| **Devinsky 2017** |  |  |
| **Bias** | **Author’s judgement** | **Support for judgement** |
| Random sequence generation (selection bias) | Low | Details in Devinsky 2018 |
| Allocation concealment (selection bias) | Low | Randomization was performed and assigned independently, held centrally, and not divulged to any other person involved in the trial until after database lock |
| Blinding of participants and personnel (performance bias) | Low | Placebo solution was identical to the cannabidiol solution |
| Blinding of outcome assessment (detection bias) | Low | Details in Devinsky 2018 |
| Incomplete outcome data (attrition bias) | Low | ITT, sensitivity analysis |
| Selective reporting (reporting bias) | Low | All outcomes reported |
| Other bias | Low |  |
| **Devinsky 2018** |  |  |
| **Bias** | **Author’s judgement** | **Support for judgement** |
| Random sequence generation (selection bias) | Low | Computer generated |
| Allocation concealment (selection bias) | Low | Held centrally by an independent statistician |
| Blinding of participants and personnel (performance bias) | Low | Both drugs were provided in identical amber glass bottles |
| Blinding of outcome assessment (detection bias) | Low | Patients or caregivers recorded using an interactive voice-response system |
| Incomplete outcome data (attrition bias) | Low | ITT, sensitivity analysis |
| Selective reporting (reporting bias) | Low | All outcomes reported |
| Other bias | Low |  |
| **De Vries 2016** |  |  |
| **Bias** | **Author’s judgement** | **Support for judgement** |
| Random sequence generation (selection bias) | Low | Computer generated |
| Allocation concealment (selection bias) | Low | Identical capsules |
| Blinding of participants and personnel (performance bias) | Low | Low dose diazepam was used as active placebo to prevent unblinding |
| Blinding of outcome assessment (detection bias) | Low | Low dose diazepam was used as active placebo to prevent unblinding |
| Incomplete outcome data (attrition bias) | Low | 1 patient was withdrawn and replaced, period analysis done |
| Selective reporting (reporting bias) | Low | All outcomes reported |
| Other bias | Unclear | Small sample size, pilot study |
| **De Vries 2017** |  |  |
| **Bias** | **Author’s judgement** | **Support for judgement** |
| Random sequence generation (selection bias) | Low | Computer generated |
| Allocation concealment (selection bias) | Low | Treatment allocation was concealed |
| Blinding of participants and personnel (performance bias) | Low | Treatment allocation was strictly concealed from participants, investigators and all other study personnel involved in the stuy |
| Blinding of outcome assessment (detection bias) | Low | Treatment allocation concealed until end of study and database lock |
| Incomplete outcome data (attrition bias) | Low | mITT analysis, including with and without dropouts |
| Selective reporting (reporting bias) | Low | All outcomes reported |
| Other bias | Unclear | Exploratory study |
| **Duran 2010** |  |  |
| **Bias** | **Author’s judgement** | **Support for judgement** |
| Random sequence generation (selection bias) | Low | Randomization stratified by sex and hospital, permuted blocks |
| Allocation concealment (selection bias) | Unclear | No information |
| Blinding of participants and personnel (performance bias) | Low | Placebo designed to match appearance, smell and taste of sativex |
| Blinding of outcome assessment (detection bias) | Unclear | No information |
| Incomplete outcome data (attrition bias) | Low | All patients analyzed |
| Selective reporting (reporting bias) | Unclear | Severity and duration of nausea; percentage of satisfaction not reported |
| Other bias | Unclear | Pilot study, small sample size |
| **Einhorn 1981** |  |  |
| **Bias** | **Author’s judgement** | **Support for judgement** |
| Random sequence generation (selection bias) | Unclear | No information |
| Allocation concealment (selection bias) | Unclear | No information |
| Blinding of participants and personnel (performance bias) | Low | Identically prepared capsules |
| Blinding of outcome assessment (detection bias) | Low | Identically prepared capsules |
| Incomplete outcome data (attrition bias) | Unclear | 20% dropouts, reasons not related to trial, no information about missing data |
| Selective reporting (reporting bias) | Low | All outcomes reported |
| Other bias | Unclear | Study design was altered allowing more dosages administration |
| **Fabre 1981** |  |  |
| **Bias** | **Author’s judgement** | **Support for judgement** |
| Random sequence generation (selection bias) | Unclear | No information |
| Allocation concealment (selection bias) | Unclear | No information |
| Blinding of participants and personnel (performance bias) | Unclear | No information |
| Blinding of outcome assessment (detection bias) | Unclear | No information |
| Incomplete outcome data (attrition bias) | Unclear | Analysis included dropouts from placebo |
| Selective reporting (reporting bias) | Low | All outcomes reported |
| Other bias | Unclear | Small sample size |
| **Fairhurst 2020** |  |  |
| **Bias** | **Author’s judgement** | **Support for judgement** |
| Random sequence generation (selection bias) | Low | Independent statistician |
| Allocation concealment (selection bias) | Low | Treatment assignment was availaboe only to the trial statistician |
| Blinding of participants and personnel (performance bias) | Low | Patients, investigational site personnel and all other trial personnel remained blinded until the database had been locked |
| Blinding of outcome assessment (detection bias) | Low | Patients, investigational site personnel and all other trial personnel remained blinded until the database had been locked |
| Incomplete outcome data (attrition bias) | Low | ITT, per protocol and sensitivity analyses |
| Selective reporting (reporting bias) | Unclear | Data from Childrens Depression Inventory and CGIC are not shown |
| Other bias | Unclear | Study in children. Outcomes validated as self-report are assessed by caregivers |
| **Fallon 2017** |  |  |
| **Bias** | **Author’s judgement** | **Support for judgement** |
| Random sequence generation (selection bias) | Unclear | No information |
| Allocation concealment (selection bias) | Unclear | No information |
| Blinding of participants and personnel (performance bias) | Unclear | No information |
| Blinding of outcome assessment (detection bias) | Unclear | No information |
| Incomplete outcome data (attrition bias) | Unclear | ITT, no information about missing data handling |
| Selective reporting (reporting bias) | Low | All outcomes reported |
| Other bias | Low |  |
| **Fox 2002** |  |  |
| **Bias** | **Author’s judgement** | **Support for judgement** |
| Random sequence generation (selection bias) | Low | Table generated by the hospital pharmacy |
| Allocation concealment (selection bias) | Low | Allocation by a pharmacist not involved in any patient contact |
| Blinding of participants and personnel (performance bias) | Low | The allocation remained concealed from investigators until the conclusion of the study |
| Blinding of outcome assessment (detection bias) | Low | Assesed by posthoc video with a blinded neurologist |
| Incomplete outcome data (attrition bias) | Unclear | No period analysis |
| Selective reporting (reporting bias) | Low | All outcomes reported |
| Other bias | Unclear | Small sample size, heterogeneity of the sample |
| **Frank 2008** |  |  |
| **Bias** | **Author’s judgement** | **Support for judgement** |
| Random sequence generation (selection bias) | Low | Treatment was allocated by random permuted blocks |
| Allocation concealment (selection bias) | Low | Code breaking envelopes |
| Blinding of participants and personnel (performance bias) | Low | Patients and all clinical personnel involved in the trial were unaware of treatment allocation at all times |
| Blinding of outcome assessment (detection bias) | Low | Patients and all clinical personnel involved in the trial were unaware of treatment allocation at all times |
| Incomplete outcome data (attrition bias) | Unclear | Missing data were substituted from the preceeding week,high drop out |
| Selective reporting (reporting bias) | Low | All outcomes reported |
| Other bias | Low |  |
| **Freeman 2020** |  |  |
| **Bias** | **Author’s judgement** | **Support for judgement** |
| Random sequence generation (selection bias) | Low | By the trial statistician by use of block randomisation |
| Allocation concealment (selection bias) | Low | Code was held by the emergency inblinding service. Medication packages were labelled and sent with anonymous participant numbers |
| Blinding of participants and personnel (performance bias) | Low | All investigators and participants remained masked throughout the duration of the trial |
| Blinding of outcome assessment (detection bias) | Low | Unmasking did not occur until after the database had been locked |
| Incomplete outcome data (attrition bias) | Low | ITT, missing data handled with Bayesian multiple imputation |
| Selective reporting (reporting bias) | Low | All outcomes reported |
| Other bias | Low |  |
| **Gilbert 1995** |  |  |
| **Bias** | **Author’s judgement** | **Support for judgement** |
| Random sequence generation (selection bias) | Low | Computer generated |
| Allocation concealment (selection bias) | Unclear | No information |
| Blinding of participants and personnel (performance bias) | Unclear | No information |
| Blinding of outcome assessment (detection bias) | Unclear | No information |
| Incomplete outcome data (attrition bias) | Unclear | High dropouts, no sensitivity analysis |
| Selective reporting (reporting bias) | Low | All outcomes reported |
| Other bias | Low |  |
| **Glass 1981** |  |  |
| **Bias** | **Author’s judgement** | **Support for judgement** |
| Random sequence generation (selection bias) | Unclear | No information |
| Allocation concealment (selection bias) | Unclear | No information |
| Blinding of participants and personnel (performance bias) | Low | Identical capsules |
| Blinding of outcome assessment (detection bias) | High | The subjects guessed correctly two thirds of the time what they received |
| Incomplete outcome data (attrition bias) | Low | All 8 subjects were evaluated, carry over analysis done |
| Selective reporting (reporting bias) | Unclear | POMS score data only reported in 4 (out of 8) |
| Other bias | High | Small size sample, with differences in dosages |
| **Grimison 2020** |  |  |
| **Bias** | **Author’s judgement** | **Support for judgement** |
| Random sequence generation (selection bias) | Low | Central web-based |
| Allocation concealment (selection bias) | Low | Central |
| Blinding of participants and personnel (performance bias) | Low | Matching placebo |
| Blinding of outcome assessment (detection bias) | Unclear | No information |
| Incomplete outcome data (attrition bias) | Low | ITT, carry-over or period effec |
| Selective reporting (reporting bias) | Low | All outcomes reported |
| Other bias | Unclear | Pilot study |
| **Gross 1983** |  |  |
| **Bias** | **Author’s judgement** | **Support for judgement** |
| Random sequence generation (selection bias) | Unclear | No information |
| Allocation concealment (selection bias) | Unclear | No information |
| Blinding of participants and personnel (performance bias) | Low | Identical appearing capsules |
| Blinding of outcome assessment (detection bias) | Unclear | No information |
| Incomplete outcome data (attrition bias) | High | Last recorded values were assumed to be the final values, no carry over analysis |
| Selective reporting (reporting bias) | Low | All outcomes reported |
| Other bias | High | Too low sample size, no washout period |
| **Hagenbach 2007** |  |  |
| **Bias** | **Author’s judgement** | **Support for judgement** |
| Random sequence generation (selection bias) | Unclear | No information |
| Allocation concealment (selection bias) | Unclear | No information |
| Blinding of participants and personnel (performance bias) | Unclear | No information |
| Blinding of outcome assessment (detection bias) | Unclear | No information |
| Incomplete outcome data (attrition bias) | Low | All patients were analyzed |
| Selective reporting (reporting bias) | Unclear | Direct comparison of phase 3 between both groups is missing |
| Other bias | Unclear | Small sample size, several protocol changes |
| **Hallak 2010** |  |  |
| **Bias** | **Author’s judgement** | **Support for judgement** |
| Random sequence generation (selection bias) | Low | Patients matched in terms of sex, years of education, age and symptom profile |
| Allocation concealment (selection bias) | Unclear | No information |
| Blinding of participants and personnel (performance bias) | Low | Drugs were prepared in identical gelatin capsules |
| Blinding of outcome assessment (detection bias) | Unclear | No information |
| Incomplete outcome data (attrition bias) | Low | Completers, no dropouts |
| Selective reporting (reporting bias) | Low | All outcomes reported |
| Other bias | Unclear | Small sample size |
| **Haney 2005** |  |  |
| **Bias** | **Author’s judgement** | **Support for judgement** |
| Random sequence generation (selection bias) | Low | Drug dose order was randomized and counterbalanced across participants |
| Allocation concealment (selection bias) | Unclear | No information |
| Blinding of participants and personnel (performance bias) | Low | Capsule dosing preceded marijuana to make it diffult to distinghush what was active |
| Blinding of outcome assessment (detection bias) | Low | Participants and research assistants were blind to capsule and marijuana strenght |
| Incomplete outcome data (attrition bias) | Unclear | Only data from completers |
| Selective reporting (reporting bias) | Unclear | Results for HSQ not reported |
| Other bias | Unclear | Laboratory study, small sample size |
| **Haney 2007** |  |  |
| **Bias** | **Author’s judgement** | **Support for judgement** |
| Random sequence generation (selection bias) | Low | Details in Haney 2005 |
| Allocation concealment (selection bias) | Unclear | No information |
| Blinding of participants and personnel (performance bias) | Low | Details in Haney 2005 |
| Blinding of outcome assessment (detection bias) | Low | Details in Haney 2005 |
| Incomplete outcome data (attrition bias) | Low | All patients analysed |
| Selective reporting (reporting bias) | Unclear | Sleep assessment not completely reported |
| Other bias | Unclear | Laboratory study, small sample size |
| **Haney 2016** |  |  |
| **Bias** | **Author’s judgement** | **Support for judgement** |
| Random sequence generation (selection bias) | Low | Details in Haney 2005 |
| Allocation concealment (selection bias) | Unclear | No information |
| Blinding of participants and personnel (performance bias) | Low | Opaque capsules, administered under double-blind conditions under observation of research staff |
| Blinding of outcome assessment (detection bias) | Low | Opaque capsules, administered under double-blind conditions under observation of research staff |
| Incomplete outcome data (attrition bias) | Unclear | No carry over or period effect analysis |
| Selective reporting (reporting bias) | Low | All outcomes reported |
| Other bias | Unclear | Laboratory study, small sample size |
| **Herrmann 2016** |  |  |
| **Bias** | **Author’s judgement** | **Support for judgement** |
| Random sequence generation (selection bias) | Unclear | No information |
| Allocation concealment (selection bias) | Unclear | No information |
| Blinding of participants and personnel (performance bias) | Unclear | No information |
| Blinding of outcome assessment (detection bias) | Unclear | No information |
| Incomplete outcome data (attrition bias) | Unclear | Only completers analyzed, no period analysis |
| Selective reporting (reporting bias) | Low | All outcomes reported |
| Other bias | Unclear | Laboratory study, small sample size |
| **Herrmann 2019a** |  |  |
| **Bias** | **Author’s judgement** | **Support for judgement** |
| Random sequence generation (selection bias) | Unclear | Randomized without stratification |
| Allocation concealment (selection bias) | Unclear | No information |
| Blinding of participants and personnel (performance bias) | Unclear | No information |
| Blinding of outcome assessment (detection bias) | Unclear | No information |
| Incomplete outcome data (attrition bias) | Low | Order analysis done |
| Selective reporting (reporting bias) | Low | All outcomes reported |
| Other bias | Unclear | Laboratory study, small sample size |
| **Herrmann 2019b** |  |  |
| **Bias** | **Author’s judgement** | **Support for judgement** |
| Random sequence generation (selection bias) | Low | Computer-generated |
| Allocation concealment (selection bias) | Low | All were blinded to treatment allocation and block size, identical-appearing placebo |
| Blinding of participants and personnel (performance bias) | Low | Study staff were unblinded only when the final patient completed all study assessments and the database was locked |
| Blinding of outcome assessment (detection bias) | Low | Study staff were unblinded only when the final patient completed all study assessments and the database was locked |
| Incomplete outcome data (attrition bias) | Low | 38 of 39 participants were analysed, no imputation, modified ITT |
| Selective reporting (reporting bias) | Low | All outcomes reported |
| Other bias | Unclear | Proportion of males greater, small sample size |
| **Hill 2017** |  |  |
| **Bias** | **Author’s judgement** | **Support for judgement** |
| Random sequence generation (selection bias) | Unclear | No information |
| Allocation concealment (selection bias) | Unclear | No information |
| Blinding of participants and personnel (performance bias) | Unclear | No information |
| Blinding of outcome assessment (detection bias) | Unclear | No information |
| Incomplete outcome data (attrition bias) | Unclear | No information about missing datan handling, ITT |
| Selective reporting (reporting bias) | Low | All outcomes reported |
| Other bias | Unclear | Pilot study, small sample size |
| **Hindocha 2018** |  |  |
| **Bias** | **Author’s judgement** | **Support for judgement** |
| Random sequence generation (selection bias) | Unclear | Balanced for gender |
| Allocation concealment (selection bias) | Low | Participant-numbered, opaque, selaed envelpes |
| Blinding of participants and personnel (performance bias) | Low | Identical capsules |
| Blinding of outcome assessment (detection bias) | Low | Concelaed from experimenters untill all data were collected and entered |
| Incomplete outcome data (attrition bias) | Low | Period and carry-over analysis |
| Selective reporting (reporting bias) | Low | All outcomes reported |
| Other bias | Unclear | Small sample size |
| **Hurd 2019** |  |  |
| **Bias** | **Author’s judgement** | **Support for judgement** |
| Random sequence generation (selection bias) | Low | By an investigator independent from the study |
| Allocation concealment (selection bias) | Low | Held centrally and not divulged to anyone involved in the trial |
| Blinding of participants and personnel (performance bias) | Low | CBD and placebo containers were indentical in appearance, taste amd composition |
| Blinding of outcome assessment (detection bias) | Low | CBD and placebo containers were indentical in appearance, taste amd composition |
| Incomplete outcome data (attrition bias) | Unclear | Only completers, no description of missing data |
| Selective reporting (reporting bias) | Low | All outcomes reported |
| Other bias | Low |  |
| **Jadoon 2016** |  |  |
| **Bias** | **Author’s judgement** | **Support for judgement** |
| Random sequence generation (selection bias) | Low | Held centrally by an independent statistician |
| Allocation concealment (selection bias) | Low | Pack number according to the randomization |
| Blinding of participants and personnel (performance bias) | Low | Not divulged to any other person involved in the study until the database had been locked |
| Blinding of outcome assessment (detection bias) | Low | Not divulged to any other person involved in the study until the database had been locked |
| Incomplete outcome data (attrition bias) | Unclear | ITT, no sample size calculation, no imputation method |
| Selective reporting (reporting bias) | Low | All outcomes reported |
| Other bias | Unclear | Pilot study, small sample size |
| **Jatoi 2002** |  |  |
| **Bias** | **Author’s judgement** | **Support for judgement** |
| Random sequence generation (selection bias) | Unclear | No information |
| Allocation concealment (selection bias) | Unclear | No information |
| Blinding of participants and personnel (performance bias) | Unclear | No information |
| Blinding of outcome assessment (detection bias) | Unclear | No information |
| Incomplete outcome data (attrition bias) | Unclear | 45% of patients completed both a baseline and 1-month follow-up. No information about missing data handling |
| Selective reporting (reporting bias) | Low | All outcomes reported |
| Other bias | Low |  |
| **Jetly 2015** |  |  |
| **Bias** | **Author’s judgement** | **Support for judgement** |
| Random sequence generation (selection bias) | Low | By the pharmacist |
| Allocation concealment (selection bias) | Unclear | No information |
| Blinding of participants and personnel (performance bias) | Low | Investigators and subjects were blind as to which period contained active medication vs placebo |
| Blinding of outcome assessment (detection bias) | Low | Investigators and subjects were blind as to which period contained active medication vs placebo |
| Incomplete outcome data (attrition bias) | Low | All participants were analyzed. mITT, period analysis done |
| Selective reporting (reporting bias) | Low | All outcomes reported |
| Other bias | Unclear | Small sample size |
| **Johansson 1982** |  |  |
| **Bias** | **Author’s judgement** | **Support for judgement** |
| Random sequence generation (selection bias) | Unclear | No information |
| Allocation concealment (selection bias) | Unclear | No information |
| Blinding of participants and personnel (performance bias) | Unclear | No information |
| Blinding of outcome assessment (detection bias) | High | No information, patient rating |
| Incomplete outcome data (attrition bias) | Unclear | High dropouts |
| Selective reporting (reporting bias) | Low | All data are reported, different periods shown |
| Other bias | Unclear | The second crossover period was worse for vomiting in general, small sample size |
| **Johnson 2010** |  |  |
| **Bias** | **Author’s judgement** | **Support for judgement** |
| Random sequence generation (selection bias) | Unclear | No information |
| Allocation concealment (selection bias) | Unclear | No information |
| Blinding of participants and personnel (performance bias) | Low | Placebo delivered only excipients plus colorants |
| Blinding of outcome assessment (detection bias) | Unclear | No information |
| Incomplete outcome data (attrition bias) | Unclear | ITT, no description of missing data handling |
| Selective reporting (reporting bias) | Low | All outcomes reported |
| Other bias | Low |  |
| **Kavia 2010** |  |  |
| **Bias** | **Author’s judgement** | **Support for judgement** |
| Random sequence generation (selection bias) | Low | Permuted blocks of four |
| Allocation concealment (selection bias) | Low | Study medication in selaed envelopes |
| Blinding of participants and personnel (performance bias) | Low | Placebo contained peppermint oil to blind the smell and taste and colorants to match the colour |
| Blinding of outcome assessment (detection bias) | Unclear | No information |
| Incomplete outcome data (attrition bias) | Unclear | High dropouts, ITT but no despcription of missing data |
| Selective reporting (reporting bias) | Low | All outcomes reported |
| Other bias | Low |  |
| **Killestein 2002** |  |  |
| **Bias** | **Author’s judgement** | **Support for judgement** |
| Random sequence generation (selection bias) | Unclear | No information |
| Allocation concealment (selection bias) | Unclear | No information |
| Blinding of participants and personnel (performance bias) | Low | 2 different persons resposible for safety and neurological tests |
| Blinding of outcome assessment (detection bias) | Low | 2 different persons resposible for safety and neurological tests |
| Incomplete outcome data (attrition bias) | Unclear | Data for all participants, no carry over test |
| Selective reporting (reporting bias) | Unclear | VAS outcomes are not fully described |
| Other bias | Unclear | Short communication format, small sample size |
| **Kleine-Brueggeney 2015** |  |  |
| **Bias** | **Author’s judgement** | **Support for judgement** |
| Random sequence generation (selection bias) | Low | Computer generated |
| Allocation concealment (selection bias) | Low | Sealed opaque envelopes |
| Blinding of participants and personnel (performance bias) | Low | The patient and all involved personnel were blinded |
| Blinding of outcome assessment (detection bias) | Low | The patient and all involved personnel were blinded |
| Incomplete outcome data (attrition bias) | Low | All patients analyzed, no dropouts |
| Selective reporting (reporting bias) | Low | All outcomes reported |
| Other bias | Unclear | Small sample size, study was discontinued due to THC side effects |
| **Klooker 2011** |  |  |
| **Bias** | **Author’s judgement** | **Support for judgement** |
| Random sequence generation (selection bias) | Unclear | No information |
| Allocation concealment (selection bias) | Unclear | No information |
| Blinding of participants and personnel (performance bias) | Unclear | No information |
| Blinding of outcome assessment (detection bias) | Unclear | No information |
| Incomplete outcome data (attrition bias) | Unclear | No order or period analysis |
| Selective reporting (reporting bias) | Unclear | Small sample size, study was discontinued due to THC side effects |
| Other bias | Unclear | Small sample size, laboratory study |
| **Lane 1991** |  |  |
| **Bias** | **Author’s judgement** | **Support for judgement** |
| Random sequence generation (selection bias) | Unclear | No information |
| Allocation concealment (selection bias) | Unclear | No information |
| Blinding of participants and personnel (performance bias) | Unclear | No information |
| Blinding of outcome assessment (detection bias) | Unclear | No information |
| Incomplete outcome data (attrition bias) | Unclear | Highest dropouts due to dronabinol side -effects. No sensitivity analysis |
| Selective reporting (reporting bias) | Low | All outcomes reported |
| Other bias | Unclear | Small sample size |
| **Langford 2013** |  |  |
| **Bias** | **Author’s judgement** | **Support for judgement** |
| Random sequence generation (selection bias) | Low | Computer generated |
| Allocation concealment (selection bias) | Low | Patients, investigators and those assesing the data were therefore blinded to the patients treatment allocation |
| Blinding of participants and personnel (performance bias) | Low | Patients, investigators and those assesing the data were therefore blinded to the patients treatment allocation |
| Blinding of outcome assessment (detection bias) | Low | Patients, investigators and those assesing the data were therefore blinded to the patients treatment allocation |
| Incomplete outcome data (attrition bias) | Unclear | ITT, no description of missing data handling |
| Selective reporting (reporting bias) | Low | All outcomes reported |
| Other bias | Low |  |
| **Leocani 2015** |  |  |
| **Bias** | **Author’s judgement** | **Support for judgement** |
| Random sequence generation (selection bias) | Unclear | No information |
| Allocation concealment (selection bias) | Unclear | No information |
| Blinding of participants and personnel (performance bias) | Unclear | No information |
| Blinding of outcome assessment (detection bias) | Unclear | No information |
| Incomplete outcome data (attrition bias) | High | No description of missing data handling or period or carry-over analysis |
| Selective reporting (reporting bias) | Unclear | Spasms are not reported |
| Other bias | Unclear | Small sample size |
| **Levin 2011** |  |  |
| **Bias** | **Author’s judgement** | **Support for judgement** |
| Random sequence generation (selection bias) | Low | Conducted by a research pharmacist independent of the research team |
| Allocation concealment (selection bias) | Low | Conducted by a research pharmacist independent of the research team |
| Blinding of participants and personnel (performance bias) | Low | Medication prepared by the pharmacy |
| Blinding of outcome assessment (detection bias) | Unclear | No information |
| Incomplete outcome data (attrition bias) | Low | All patients analyted, ITT, sensitivity analysis |
| Selective reporting (reporting bias) | Unclear | Craving (MCQ) is not shown |
| Other bias | Low |  |
| **Levin 2017** |  |  |
| **Bias** | **Author’s judgement** | **Support for judgement** |
| Random sequence generation (selection bias) | Low | Computer generated |
| Allocation concealment (selection bias) | Low | Seal opaque envelopes |
| Blinding of participants and personnel (performance bias) | Low | Pills dentical in appearance |
| Blinding of outcome assessment (detection bias) | Low | Data analysis were blinded to group allocation for the duration of the study |
| Incomplete outcome data (attrition bias) | Low | Data were availble for all participants, ITT. no imputation. |
| Selective reporting (reporting bias) | Low | All results are reported |
| Other bias | Low |  |
| **Leweke 2012** |  |  |
| **Bias** | **Author’s judgement** | **Support for judgement** |
| Random sequence generation (selection bias) | Low | Randomization (1:1) sequence prepared by a person otherwise not involved in the study |
| Allocation concealment (selection bias) | Low | The hospital pharmacy provided individula medication kits according to the randomization |
| Blinding of participants and personnel (performance bias) | Unclear | No information |
| Blinding of outcome assessment (detection bias) | Unclear | No information |
| Incomplete outcome data (attrition bias) | Low | mITT, PP |
| Selective reporting (reporting bias) | Low | All outcomes reported |
| Other bias | Unclear | Small sample size, early termination of the study, low power |
| **Lewis 1994** |  |  |
| **Bias** | **Author’s judgement** | **Support for judgement** |
| Random sequence generation (selection bias) | Unclear | No information |
| Allocation concealment (selection bias) | Unclear | No information |
| Blinding of participants and personnel (performance bias) | Low | The anaesthetist, recovery and nursing staff were all blinded to the allocated premedication |
| Blinding of outcome assessment (detection bias) | Low | Nursing staff were all blinded to the allocated premedication |
| Incomplete outcome data (attrition bias) | Low | 7 out of 60 excluded, due to non clinical reasons, equal from both groups |
| Selective reporting (reporting bias) | Low | All outcomes reported |
| Other bias | Low |  |
| **Lichtman 2018** |  |  |
| **Bias** | **Author’s judgement** | **Support for judgement** |
| Random sequence generation (selection bias) | Unclear | No information |
| Allocation concealment (selection bias) | Unclear | No information |
| Blinding of participants and personnel (performance bias) | Unclear | No information |
| Blinding of outcome assessment (detection bias) | Unclear | No information |
| Incomplete outcome data (attrition bias) | Unclear | ITT, no description of missing data handling |
| Selective reporting (reporting bias) | Low | All outcomes reported |
| Other bias | Low |  |
| **Lintzeris 2019** |  |  |
| **Bias** | **Author’s judgement** | **Support for judgement** |
| Random sequence generation (selection bias) | Low | Independent researcher, 8-block permuted randomizatuion |
| Allocation concealment (selection bias) | Low | Matching placebo used the same carrier and flavoring, in canisters indentically labeled |
| Blinding of participants and personnel (performance bias) | Low | Participants, clinicians, and researchers were blind to allocation |
| Blinding of outcome assessment (detection bias) | Low | Participants, clinicians, and researchers were blind to allocation |
| Incomplete outcome data (attrition bias) | Low | ITT with sensitivity analysis (multilevel multiple-imputation) |
| Selective reporting (reporting bias) | Low | All outcomes reported |
| Other bias | Low |  |
| **Lofwall 2016** |  |  |
| **Bias** | **Author’s judgement** | **Support for judgement** |
| Random sequence generation (selection bias) | Unclear | Dose order was fully randomized except that 20mg always preceded 30 mg |
| Allocation concealment (selection bias) | Low | All active doses were over-encapsulated and loose-filled with lactose |
| Blinding of participants and personnel (performance bias) | Unclear | All doses in blue/white gelatin capsules |
| Blinding of outcome assessment (detection bias) | Unclear | No information |
| Incomplete outcome data (attrition bias) | High | 3 missing data due to adverse effect in the high dose, no carry over test, no sensitivity analysis |
| Selective reporting (reporting bias) | Unclear | The highest dose was excluded from all analyses |
| Other bias | High | Change in doses due to adverse effects, all females, small sample size |
| **López-Sendón 2016** |  |  |
| **Bias** | **Author’s judgement** | **Support for judgement** |
| Random sequence generation (selection bias) | Low | By Biostatistical Service |
| Allocation concealment (selection bias) | Low | The placebo was a solvent packed in containers identic to those of the studied drug |
| Blinding of participants and personnel (performance bias) | Low | Patients and clinical raters were blind to treatment allocation |
| Blinding of outcome assessment (detection bias) | Low | Patients and clinical raters were blind to treatment allocation |
| Incomplete outcome data (attrition bias) | Low | Data from two periods analyzed |
| Selective reporting (reporting bias) | Low | All outcomes reported |
| Other bias | Unclear | Small sample size, baseline differences |
| **Lundahl 2015** |  |  |
| **Bias** | **Author’s judgement** | **Support for judgement** |
| Random sequence generation (selection bias) | Unclear | No information |
| Allocation concealment (selection bias) | Unclear | No information |
| Blinding of participants and personnel (performance bias) | Low | Each dose was encapsulated in opaque, similarly colored capsules |
| Blinding of outcome assessment (detection bias) | Unclear | No information |
| Incomplete outcome data (attrition bias) | Unclear | No period analysis |
| Selective reporting (reporting bias) | Low | All outcomes reported |
| Other bias | Unclear | Small sample size, laboratory study |
| **Lynch 2014** |  |  |
| **Bias** | **Author’s judgement** | **Support for judgement** |
| Random sequence generation (selection bias) | Low | Computer generated |
| Allocation concealment (selection bias) | Low | Randomization code Placebo was packaged in exaclty the same way |
| Blinding of participants and personnel (performance bias) | Low | Placebo was packaged in exaclty the same way |
| Blinding of outcome assessment (detection bias) | Low | Participants and study staff were blinded to the randomization cose, which was not broken until the completion of the study |
| Incomplete outcome data (attrition bias) | Unclear | No description of missing data |
| Selective reporting (reporting bias) | Low | All outcomes reported |
| Other bias | Unclear | Pilot study, small sample size |
| **Malik 2017** |  |  |
| **Bias** | **Author’s judgement** | **Support for judgement** |
| Random sequence generation (selection bias) | Unclear | No information |
| Allocation concealment (selection bias) | Low | By the providing pharmacist |
| Blinding of participants and personnel (performance bias) | Low | Placebo capsules were matched accordingly to resemble dronabinol capsules |
| Blinding of outcome assessment (detection bias) | Low | Performed by a physician blinded to patient treatment |
| Incomplete outcome data (attrition bias) | Unclear | Dropouts were not analyzed, no period analysis |
| Selective reporting (reporting bias) | Low | All outcomes reported |
| Other bias | Unclear | Pilot study, small sample size |
| **Markova 2019** |  |  |
| **Bias** | **Author’s judgement** | **Support for judgement** |
| Random sequence generation (selection bias) | Unclear | No information |
| Allocation concealment (selection bias) | Unclear | No information |
| Blinding of participants and personnel (performance bias) | Unclear | No information |
| Blinding of outcome assessment (detection bias) | Unclear | No information |
| Incomplete outcome data (attrition bias) | Low | ITT, PP, LOCF, sensitivity analysis |
| Selective reporting (reporting bias) | Low | All outcomes reported |
| Other bias | Low |  |
| **Masataka 2019** |  |  |
| **Bias** | **Author’s judgement** | **Support for judgement** |
| Random sequence generation (selection bias) | Unclear | No information |
| Allocation concealment (selection bias) | Low | Container provided to a clinician who did not know whether the bottle contained CBD |
| Blinding of participants and personnel (performance bias) | Low | The participants and the investigators were blinded regarding which condition under which eah participant was studied |
| Blinding of outcome assessment (detection bias) | Low | The participants and the investigators were blinded regarding which condition under which eah participant was studied |
| Incomplete outcome data (attrition bias) | Unclear | No ITT, only completers |
| Selective reporting (reporting bias) | Low | All outcomes reported |
| Other bias | Unclear | Pilot study, small sample size |
| **McGuire 2018** |  |  |
| **Bias** | **Author’s judgement** | **Support for judgement** |
| Random sequence generation (selection bias) | Low | Produced by an independent statistician held centrally |
| Allocation concealment (selection bias) | Low | Cartons and bottles were identical in appearance |
| Blinding of participants and personnel (performance bias) | Low | Not divulged to anyone inviolved in the trial |
| Blinding of outcome assessment (detection bias) | Low | Participants or investigators did not distinguish preparations or treatment groups on the bases of adverce effects |
| Incomplete outcome data (attrition bias) | Unclear | ITT, missing data with LOCF |
| Selective reporting (reporting bias) | Unclear | Patients and caregiver global impression of change not reported |
| Other bias | Low |  |
| **Meiri 2007** |  |  |
| **Bias** | **Author’s judgement** | **Support for judgement** |
| Random sequence generation (selection bias) | Unclear | No information. Difficulties from reluctance on the part of patients to potential randomization to placebo |
| Allocation concealment (selection bias) | Unclear | No information |
| Blinding of participants and personnel (performance bias) | Low | Patients were dosed QID to preserve the blind |
| Blinding of outcome assessment (detection bias) | Unclear | No information |
| Incomplete outcome data (attrition bias) | Unclear | ITT, missing data with LOCF |
| Selective reporting (reporting bias) | Low | All outcomes reported |
| Other bias | Unclear | Small sample size |
| **Meneses-Gaya 2020** |  |  |
| **Bias** | **Author’s judgement** | **Support for judgement** |
| Random sequence generation (selection bias) | Unclear | No information |
| Allocation concealment (selection bias) | Unclear | No information |
| Blinding of participants and personnel (performance bias) | Low | Identical gelatin capsules |
| Blinding of outcome assessment (detection bias) | Unclear | No information |
| Incomplete outcome data (attrition bias) | Low | ITT with mixed model approach for missing data |
| Selective reporting (reporting bias) | Low | All outcomes reported |
| Other bias | Unclear | Small sample size |
| **Miller 2020** |  |  |
| **Bias** | **Author’s judgement** | **Support for judgement** |
| Random sequence generation (selection bias) | Low | Computer generated |
| Allocation concealment (selection bias) | Unclear | No information |
| Blinding of participants and personnel (performance bias) | Low | Treatments provided in identical amber glass bottles |
| Blinding of outcome assessment (detection bias) | Low | Caregivers, and individuals assessing the data, were unaware of the allocation of the patients and remained unaware until trial completion |
| Incomplete outcome data (attrition bias) | Low | ITT, sensitivity analysis |
| Selective reporting (reporting bias) | Low | All outcomes reported |
| Other bias | Low |  |
| **Mongeau-Perusse 2021** |  |  |
| **Bias** | **Author’s judgement** | **Support for judgement** |
| Random sequence generation (selection bias) | Low | An independent biostatistician created a computer generated randomized sequence |
| Allocation concealment (selection bias) | Low | The pharmacy kept treatment assigments in separate envelopes |
| Blinding of participants and personnel (performance bias) | Low | Solutions looked and tasted exaclty alike |
| Blinding of outcome assessment (detection bias) | Low | Participant and research staff were blinded to treatment allocation, James blinding index was used to evaluate treatment blinding |
| Incomplete outcome data (attrition bias) | Low | Data avalable for all participants, sensitivity analysis |
| Selective reporting (reporting bias) | Low | All outcomes reported |
| Other bias | Unclear | Lost to follow-up without relapse events were considered as having relapsed, missing urine tests as positive |
| **Morgan 2013** |  |  |
| **Bias** | **Author’s judgement** | **Support for judgement** |
| Random sequence generation (selection bias) | Unclear | No information |
| Allocation concealment (selection bias) | Unclear | No information |
| Blinding of participants and personnel (performance bias) | Unclear | No information |
| Blinding of outcome assessment (detection bias) | Unclear | No information |
| Incomplete outcome data (attrition bias) | Unclear | No information about missing data or dropouts |
| Selective reporting (reporting bias) | Low | All outcomes reported |
| Other bias | Unclear | Pilot study, small sample size |
| **Müller-Vahl 2001/2002** |  |  |
| **Bias** | **Author’s judgement** | **Support for judgement** |
| Random sequence generation (selection bias) | Low | By a psychiatrist not involved in the study |
| Allocation concealment (selection bias) | Low | Randomization codes |
| Blinding of participants and personnel (performance bias) | Low | Visually identical placebo and THC capsules |
| Blinding of outcome assessment (detection bias) | Low | Noone had access to the randimozation codes during the study |
| Incomplete outcome data (attrition bias) | Low | All participants analysed, carry over test |
| Selective reporting (reporting bias) | Low | All outcomes reported |
| Other bias | Unclear | Pilot study, small sample size |
| **Müller-Vahl 2003/2003b** |  |  |
| **Bias** | **Author’s judgement** | **Support for judgement** |
| Random sequence generation (selection bias) | Low | By a psychiatrist not involved in the study |
| Allocation concealment (selection bias) | Low | Randomization codes |
| Blinding of participants and personnel (performance bias) | Low | Visually identical placebo and THC capsules |
| Blinding of outcome assessment (detection bias) | Low | Noone had access to the randimozation codes during the study |
| Incomplete outcome data (attrition bias) | Unclear | 4 dropuouts, analysis include only data from patients who completed the study |
| Selective reporting (reporting bias) | Low | All outcomes reported |
| Other bias | Unclear | Pilot study, small sample size |
| **Narang 2008** |  |  |
| **Bias** | **Author’s judgement** | **Support for judgement** |
| Random sequence generation (selection bias) | Unclear | Randomization scheme |
| Allocation concealment (selection bias) | Low | By the Investigational Drug Service Pharmacy of the hospital |
| Blinding of participants and personnel (performance bias) | Low | Study personnel and subjects were blinded until all the subjects had completed the phase 1 |
| Blinding of outcome assessment (detection bias) | Low | Results were not examined until the subjects had finished both phases |
| Incomplete outcome data (attrition bias) | Low | All participants were evaluated, 1 droupout |
| Selective reporting (reporting bias) | Low | All outcomes reported |
| Other bias | Unclear | Heterogeneous population with opioid and THC exposure history, small sample size |
| **Neidhardt 1981** |  |  |
| **Bias** | **Author’s judgement** | **Support for judgement** |
| Random sequence generation (selection bias) | Unclear | No information |
| Allocation concealment (selection bias) | Low | Antiemetics were dispensed by the pharmacy from a randomized list |
| Blinding of participants and personnel (performance bias) | Low | Identical capsules |
| Blinding of outcome assessment (detection bias) | Unclear | No information |
| Incomplete outcome data (attrition bias) | High | High dropouts, no ITT or sensitivity analysis |
| Selective reporting (reporting bias) | Unclear | All outcomes reported but preference reported only for 13 completer patients |
| Other bias | Unclear | No statistcs described |
| **Niederle 1986** |  |  |
| **Bias** | **Author’s judgement** | **Support for judgement** |
| Random sequence generation (selection bias) | Unclear | No information |
| Allocation concealment (selection bias) | Unclear | No information |
| Blinding of participants and personnel (performance bias) | Unclear | No information |
| Blinding of outcome assessment (detection bias) | Unclear | No information |
| Incomplete outcome data (attrition bias) | Low | No dropoupts |
| Selective reporting (reporting bias) | Low | All outcomes reported |
| Other bias | High | Significant differences only in one of the 2 crossover periods, small sample size |
| **Niiranen 1985** |  |  |
| **Bias** | **Author’s judgement** | **Support for judgement** |
| Random sequence generation (selection bias) | Unclear | No information |
| Allocation concealment (selection bias) | Unclear | No information |
| Blinding of participants and personnel (performance bias) | Low | Identically appearing capsules |
| Blinding of outcome assessment (detection bias) | Unclear | No information |
| Incomplete outcome data (attrition bias) | High | 8 dropouts (3 from nabilone), no ITT or sensitivity analysis.Only patients who had completed the cross-over study were evaluated |
| Selective reporting (reporting bias) | Low | All outcomes reported |
| Other bias | Unclear | Small sample size |
| **Notcutt 2012** |  |  |
| **Bias** | **Author’s judgement** | **Support for judgement** |
| Random sequence generation (selection bias) | Low | Independent statistician using a computer algorithm |
| Allocation concealment (selection bias) | Unclear | No information |
| Blinding of participants and personnel (performance bias) | Low | Blinding was maintained throughout and beyond the study |
| Blinding of outcome assessment (detection bias) | Low | Blinding was maintained throughout and beyond the study |
| Incomplete outcome data (attrition bias) | Unclear | ITT, no description of data handling method |
| Selective reporting (reporting bias) | Low | All outcomes reported |
| Other bias | Unclear | Small sample size, limited power |
| **Novotna 2011** |  |  |
| **Bias** | **Author’s judgement** | **Support for judgement** |
| Random sequence generation (selection bias) | Unclear | No information |
| Allocation concealment (selection bias) | Unclear | No information |
| Blinding of participants and personnel (performance bias) | Unclear | No information |
| Blinding of outcome assessment (detection bias) | Unclear | No information |
| Incomplete outcome data (attrition bias) | Unclear | ITT, no description of data handling method |
| Selective reporting (reporting bias) | Low | All outcomes reported |
| Other bias | Low |  |
| **Noyes 1975** |  |  |
| **Bias** | **Author’s judgement** | **Support for judgement** |
| Random sequence generation (selection bias) | Unclear | No information |
| Allocation concealment (selection bias) | Unclear | No information |
| Blinding of participants and personnel (performance bias) | Low | Medication Identical appearance |
| Blinding of outcome assessment (detection bias) | Unclear | No information |
| Incomplete outcome data (attrition bias) | Unclear | Only completers, few dropouts, no period analysis |
| Selective reporting (reporting bias) | Low | All outcomes reported |
| Other bias | Unclear | Small sample size |
| **Nurmikko 2007** |  |  |
| **Bias** | **Author’s judgement** | **Support for judgement** |
| Random sequence generation (selection bias) | Low | Computer generated |
| Allocation concealment (selection bias) | Low | Held by the sponsor, medication provided in identical amber vials |
| Blinding of participants and personnel (performance bias) | Low | Placebo was identical in composition, appearance, odour and taste, peppermint oil in both preparations. |
| Blinding of outcome assessment (detection bias) | Low | Placebo was identical in composition, appearance, odour and taste, peppermint oil in both preparations. |
| Incomplete outcome data (attrition bias) | Unclear | ITT, no description of data handling method |
| Selective reporting (reporting bias) | Low | All outcomes reported |
| Other bias | Low |  |
| **O’Neil 2020** |  |  |
| **Bias** | **Author’s judgement** | **Support for judgement** |
| Random sequence generation (selection bias) | Low | Generated by a research pharmacist |
| Allocation concealment (selection bias) | Unclear | No information |
| Blinding of participants and personnel (performance bias) | Low | Visually identical placebo capsule |
| Blinding of outcome assessment (detection bias) | Unclear | No information |
| Incomplete outcome data (attrition bias) | Unclear | No period or carry-over analysis |
| Selective reporting (reporting bias) | Low | All outcomes reported |
| Other bias | Unclear | Small sample size |
| **Orr 1981** |  |  |
| **Bias** | **Author’s judgement** | **Support for judgement** |
| Random sequence generation (selection bias) | Low | Courses of drugs in any of six possible sequences |
| Allocation concealment (selection bias) | Unclear | No information |
| Blinding of participants and personnel (performance bias) | Unclear | No information |
| Blinding of outcome assessment (detection bias) | Unclear | No information |
| Incomplete outcome data (attrition bias) | High | High droupouts, no ITT or sensitivity analysis |
| Selective reporting (reporting bias) | Low | All outcomes reported |
| Other bias | Unclear | No information about wash out periods |
| **Peball 2020** |  |  |
| **Bias** | **Author’s judgement** | **Support for judgement** |
| Random sequence generation (selection bias) | Low | Computer generated |
| Allocation concealment (selection bias) | Low | boxes were labeled consecutively according to the randomization list |
| Blinding of participants and personnel (performance bias) | Low | Neither a member of the study team nor the participants were informed about treatment assigments |
| Blinding of outcome assessment (detection bias) | Low | Blinding of safety was performed during the trial via de safety data monitoring board |
| Incomplete outcome data (attrition bias) | Low | All participants included (no dropouts) |
| Selective reporting (reporting bias) | Low | All outcomes reported |
| Other bias | Unclear | Small sample size |
| **Pini 2012** |  |  |
| **Bias** | **Author’s judgement** | **Support for judgement** |
| Random sequence generation (selection bias) | Low | Computer generated |
| Allocation concealment (selection bias) | Low | Identical white capsules, randomized in two containers |
| Blinding of participants and personnel (performance bias) | Unclear | No information |
| Blinding of outcome assessment (detection bias) | Unclear | No information |
| Incomplete outcome data (attrition bias) | Unclear | 4 out of 30 participants excluded, no information about missing data |
| Selective reporting (reporting bias) | Unclear | Data of 2 periods are partially mentioned, not fully reported |
| Other bias | Unclear | Small sample size |
| **Pinsger 2006** |  |  |
| **Bias** | **Author’s judgement** | **Support for judgement** |
| Random sequence generation (selection bias) | Unclear | No information |
| Allocation concealment (selection bias) | Unclear | No information |
| Blinding of participants and personnel (performance bias) | Unclear | No information |
| Blinding of outcome assessment (detection bias) | Unclear | No information |
| Incomplete outcome data (attrition bias) | Unclear | Missing data where handled with LOCF, no carry over or period analysis |
| Selective reporting (reporting bias) | Low | All outcomes reported |
| Other bias | Unclear | Small sample size |
| **Pomeroy 1986** |  |  |
| **Bias** | **Author’s judgement** | **Support for judgement** |
| Random sequence generation (selection bias) | Unclear | No information |
| Allocation concealment (selection bias) | Unclear | No information |
| Blinding of participants and personnel (performance bias) | Low | White capsules of identical appearance |
| Blinding of outcome assessment (detection bias) | Unclear | No information |
| Incomplete outcome data (attrition bias) | Unclear | Missing data not handled |
| Selective reporting (reporting bias) | Low | All outcomes reported |
| Other bias | Unclear | Small sample size |
| **Pooyania 2010** |  |  |
| **Bias** | **Author’s judgement** | **Support for judgement** |
| Random sequence generation (selection bias) | Low | Computer generated by a pharmacist |
| Allocation concealment (selection bias) | Low | By a pharmacist |
| Blinding of participants and personnel (performance bias) | Unclear | No information |
| Blinding of outcome assessment (detection bias) | Unclear | No information |
| Incomplete outcome data (attrition bias) | Unclear | 1 drouput; no carry over test shown |
| Selective reporting (reporting bias) | Low | All outcomes reported |
| Other bias | High | Small sample size, pilot study, different baselines |
| **Portenoy 2012** |  |  |
| **Bias** | **Author’s judgement** | **Support for judgement** |
| Random sequence generation (selection bias) | Low | By computer using a block approach |
| Allocation concealment (selection bias) | Unclear | No information |
| Blinding of participants and personnel (performance bias) | Low | Matched pills |
| Blinding of outcome assessment (detection bias) | Unclear | No information |
| Incomplete outcome data (attrition bias) | Unclear | ITT, no description of missing data handling |
| Selective reporting (reporting bias) | Low | All outcomes reported |
| Other bias | Low |  |
| **Priestman 1987** |  |  |
| **Bias** | **Author’s judgement** | **Support for judgement** |
| Random sequence generation (selection bias) | Unclear | No information |
| Allocation concealment (selection bias) | Unclear | No information |
| Blinding of participants and personnel (performance bias) | Low | Identical capsules |
| Blinding of outcome assessment (detection bias) | Unclear | No information |
| Incomplete outcome data (attrition bias) | High | No carry over test shown, 7 patients were crossed over to the other drug, no information how was this statistically analysed. |
| Selective reporting (reporting bias) | Unclear | Not all the days are reported |
| Other bias | High | Patients who failed to respond to therapy or who experienced adverse reaction crossed over to the altenative agent; insufficient information on patients and methods, small sample size |
| **Rabinak 2020** |  |  |
| **Bias** | **Author’s judgement** | **Support for judgement** |
| Random sequence generation (selection bias) | Unclear | No information |
| Allocation concealment (selection bias) | Unclear | No information |
| Blinding of participants and personnel (performance bias) | Low | Participants and research staff were blinded to the contents of the capsules and subsequent drug grouping |
| Blinding of outcome assessment (detection bias) | Low | Participants and research staff were blinded to the contents of the capsules and subsequent drug grouping |
| Incomplete outcome data (attrition bias) | Low | Exclusions not related to treatments |
| Selective reporting (reporting bias) | Low | All outcomes reported |
| Other bias | Unclear | Laboratory study |
| **Rintala 2010** |  |  |
| **Bias** | **Author’s judgement** | **Support for judgement** |
| Random sequence generation (selection bias) | Low | Table of random numbers |
| Allocation concealment (selection bias) | Low | Both capsules looked identical, with a blinded code |
| Blinding of participants and personnel (performance bias) | Low | Medication identifiable only by the Veterans Affaris research pharmacy and the principal investigator |
| Blinding of outcome assessment (detection bias) | Low | Co-investigators were blinded to the order in which the medications were received |
| Incomplete outcome data (attrition bias) | Low | 2 excluded before randomization, order effect analysis |
| Selective reporting (reporting bias) | Low | All outcomes reported |
| Other bias | Unclear | Small sample size, pilot study |
| **Riva 2019** |  |  |
| **Bias** | **Author’s judgement** | **Support for judgement** |
| Random sequence generation (selection bias) | Low | Centrally, computer based |
| Allocation concealment (selection bias) | Low | Treatment allocation code was kept in sealed opaque envelope. Both solutions transparent and idistinguishable |
| Blinding of participants and personnel (performance bias) | Low | All participants, investigators, site personnel, steering committee members, and the study statistician were masked to the treatment allocation |
| Blinding of outcome assessment (detection bias) | Low | All participants, investigators, site personnel, steering committee members, and the study statistician were masked to the treatment allocation |
| Incomplete outcome data (attrition bias) | Low | mITT |
| Selective reporting (reporting bias) | Low | All outcomes reported |
| Other bias | Low |  |
| **Rog 2005** |  |  |
| **Bias** | **Author’s judgement** | **Support for judgement** |
| Random sequence generation (selection bias) | Low | Predetermined code drawn by a statistician |
| Allocation concealment (selection bias) | Low | Contained in individually sealed envelopes |
| Blinding of participants and personnel (performance bias) | Low | Placebo was designed to match the appearance, smell, and taste of the active formulation |
| Blinding of outcome assessment (detection bias) | Low | The identity of study medication, to which all study personnel remained blinded |
| Incomplete outcome data (attrition bias) | Unclear | ITT, no description of missing data handling |
| Selective reporting (reporting bias) | Low | All outcomes reported |
| Other bias | Low |  |
| **Sallan 1975** |  |  |
| **Bias** | **Author’s judgement** | **Support for judgement** |
| Random sequence generation (selection bias) | Low | Randomization to one of the 4 sequences |
| Allocation concealment (selection bias) | Unclear | No information |
| Blinding of participants and personnel (performance bias) | Low | Capsules identical in appearance |
| Blinding of outcome assessment (detection bias) | Unclear | No information |
| Incomplete outcome data (attrition bias) | Unclear | High dropouts,no period analysis |
| Selective reporting (reporting bias) | Unclear | Washout period not described, statistics not fully reported |
| Other bias | Unclear | Small sample size |
| **Sallan 1980** |  |  |
| **Bias** | **Author’s judgement** | **Support for judgement** |
| Random sequence generation (selection bias) | Low | Randomization to one of the six distinct ways |
| Allocation concealment (selection bias) | Unclear | No information |
| Blinding of participants and personnel (performance bias) | Low | Neither the person administering the drug nor the one recording the patient's response knew which drug the patient received |
| Blinding of outcome assessment (detection bias) | Low | The one recording the patient's response did not know which drug the patient received |
| Incomplete outcome data (attrition bias) | Unclear | High dropouts,no period analysis |
| Selective reporting (reporting bias) | Unclear | Washout period not described, statistics not fully reported |
| Other bias | Low |  |
| **Schimrigk 2017** |  |  |
| **Bias** | **Author’s judgement** | **Support for judgement** |
| Random sequence generation (selection bias) | Low | Computer generated |
| Allocation concealment (selection bias) | Low | Block packs allocated in chronological order |
| Blinding of participants and personnel (performance bias) | Low | Blinding of patients, investigators and staff involved in the study was maintained thriughout the trial |
| Blinding of outcome assessment (detection bias) | Low | Blinding of patients, investigators and staff involved in the study was maintained thriughout the trial |
| Incomplete outcome data (attrition bias) | Unclear | No description on missing data handling |
| Selective reporting (reporting bias) | Low | All outcomes reported |
| Other bias | Low |  |
| **Selvarajah 2010** |  |  |
| **Bias** | **Author’s judgement** | **Support for judgement** |
| Random sequence generation (selection bias) | Unclear | No information |
| Allocation concealment (selection bias) | Unclear | No information |
| Blinding of participants and personnel (performance bias) | Unclear | No information |
| Blinding of outcome assessment (detection bias) | Unclear | No information |
| Incomplete outcome data (attrition bias) | Unclear | ITT, no description of missing data handling |
| Selective reporting (reporting bias) | Low | All outcomes reported |
| Other bias | Unclear | Brief report, not enough information, small sample size |
| **Serpell 2014** |  |  |
| **Bias** | **Author’s judgement** | **Support for judgement** |
| Random sequence generation (selection bias) | Low | Computer generated |
| Allocation concealment (selection bias) | Low | The investigator staff held sealed code break envelope |
| Blinding of participants and personnel (performance bias) | Low | Both sprays contained peppermint oil to blind smell and taste |
| Blinding of outcome assessment (detection bias) | Low | As such, participants, investigators and caregivers were all blinded to the treatment allocation |
| Incomplete outcome data (attrition bias) | Unclear | ITT, no description of missing data handling |
| Selective reporting (reporting bias) | Low | All outcomes reported |
| Other bias | Low |  |
| **Sieradzan 2001** |  |  |
| **Bias** | **Author’s judgement** | **Support for judgement** |
| Random sequence generation (selection bias) | Unclear | No information |
| Allocation concealment (selection bias) | Unclear | No information |
| Blinding of participants and personnel (performance bias) | Low | Same color and taste |
| Blinding of outcome assessment (detection bias) | Low | By an assessor blinded to the treatment |
| Incomplete outcome data (attrition bias) | Unclear | No carry over test |
| Selective reporting (reporting bias) | Unclear | Some secondary outcomes are not reported |
| Other bias | Unclear | Small sample size, pilot study |
| **Skrabek 2008** |  |  |
| **Bias** | **Author’s judgement** | **Support for judgement** |
| Random sequence generation (selection bias) | Low | By the HSC pharmacy |
| Allocation concealment (selection bias) | Unclear | No information |
| Blinding of participants and personnel (performance bias) | Low | Physicians and subjects were blinded to the randomization |
| Blinding of outcome assessment (detection bias) | Low | Medication was identical to placebo |
| Incomplete outcome data (attrition bias) | Unclear | 7 dropouts, 5 from nabilone, no information about missing data, no sensitivity analysis |
| Selective reporting (reporting bias) | High | Only values and stats from nabilone are shown |
| Other bias | Unclear | Small sample size |
| **Strasser 2006** |  |  |
| **Bias** | **Author’s judgement** | **Support for judgement** |
| Random sequence generation (selection bias) | Low | By a naive statistician |
| Allocation concealment (selection bias) | Low | Sealed envelopes |
| Blinding of participants and personnel (performance bias) | Low | Statistician and data manager were naive to clinical evaluations |
| Blinding of outcome assessment (detection bias) | Low | Investigators remained blinded until the study ended |
| Incomplete outcome data (attrition bias) | Low | ITT with missing substituted by the nearest-neighbor, sentitivity with LOCF and worst-case approach |
| Selective reporting (reporting bias) | Low | All outcomes reported |
| Other bias | Unclear | Study termination after interim analysis |
| **Svendsen 2004** |  |  |
| **Bias** | **Author’s judgement** | **Support for judgement** |
| Random sequence generation (selection bias) | Low | Computer generated |
| Allocation concealment (selection bias) | Low | Identical containers, taste and smell. Sealed, completely opaque code envelopes containing information about treatment sequence for each patient were present at the study site |
| Blinding of participants and personnel (performance bias) | Low | The code envelopes were returned unopened to the monitor after termination of the study |
| Blinding of outcome assessment (detection bias) | Low | We maintained blinding until the data analysis was completed |
| Incomplete outcome data (attrition bias) | Low | Data from all patients and period analysis |
| Selective reporting (reporting bias) | Low | All outcomes reported |
| Other bias | Unclear | Small sample size |
| **Thiele 2018** |  |  |
| **Bias** | **Author’s judgement** | **Support for judgement** |
| Random sequence generation (selection bias) | Low | Produced by an independent statistician and stratified by age group |
| Allocation concealment (selection bias) | Low | Drug supplied by QW Pharmaceuticals |
| Blinding of participants and personnel (performance bias) | Low | Identical amber glass bottles |
| Blinding of outcome assessment (detection bias) | Low | Individuals assesing data were masked to group assigment |
| Incomplete outcome data (attrition bias) | Low | ITT, PP, sensitivity analyses |
| Selective reporting (reporting bias) | Low | All outcomes reported |
| Other bias | Low |  |
| **Thiele 2020** |  |  |
| **Bias** | **Author’s judgement** | **Support for judgement** |
| Random sequence generation (selection bias) | Low | Produced by an independent statistician and stratified by age group |
| Allocation concealment (selection bias) | Low | Details in Thiele 2018 |
| Blinding of participants and personnel (performance bias) | Low | Details in Thiele 2018 |
| Blinding of outcome assessment (detection bias) | Low | Details in Thiele 2018 |
| Incomplete outcome data (attrition bias) | Low | ITT, PP, sensitivity analyses |
| Selective reporting (reporting bias) | Low | All outcomes reported |
| Other bias | Low |  |
| **Tomida 2006** |  |  |
| **Bias** | **Author’s judgement** | **Support for judgement** |
| Random sequence generation (selection bias) | Unclear | No information |
| Allocation concealment (selection bias) | Unclear | No information |
| Blinding of participants and personnel (performance bias) | Unclear | No information |
| Blinding of outcome assessment (detection bias) | Low | IOP measurements were obtained by the same investigator |
| Incomplete outcome data (attrition bias) | Unclear | No period analysis |
| Selective reporting (reporting bias) | Low | All outcomes reported |
| Other bias | Unclear | Pilot study, small sample size |
| **Toth 2012** |  |  |
| **Bias** | **Author’s judgement** | **Support for judgement** |
| Random sequence generation (selection bias) | Low | Block randomization |
| Allocation concealment (selection bias) | Low | By an outside coordinator |
| Blinding of participants and personnel (performance bias) | Low | Capsules identical size, color, taste and smell |
| Blinding of outcome assessment (detection bias) | Unclear | Potential unmasking occured in 62% of both groups |
| Incomplete outcome data (attrition bias) | Unclear | Missing values handled with last value carried forward |
| Selective reporting (reporting bias) | Low | All outcomes reported |
| Other bias | Unclear | Small sample size |
| **Trigo 2016** |  |  |
| **Bias** | **Author’s judgement** | **Support for judgement** |
| Random sequence generation (selection bias) | Unclear | No information |
| Allocation concealment (selection bias) | Unclear | No information |
| Blinding of participants and personnel (performance bias) | Unclear | No information |
| Blinding of outcome assessment (detection bias) | Unclear | No information |
| Incomplete outcome data (attrition bias) | Unclear | Only completers were analyzed, no period analysis |
| Selective reporting (reporting bias) | Low | All outcomes reported |
| Other bias | Unclear | Laboratory pilot study, small sample size |
| **Trigo 2018** |  |  |
| **Bias** | **Author’s judgement** | **Support for judgement** |
| Random sequence generation (selection bias) | Low | Blocks of 10 to one of the two groups in a 1.1 ratio |
| Allocation concealment (selection bias) | Low | Randomization double blind manner by the participating pharmacy |
| Blinding of participants and personnel (performance bias) | Low | All study staff except for the participating pharmacy were blinded after assignment to interventions |
| Blinding of outcome assessment (detection bias) | Low | All study staff except for the participating pharmacy were blinded after assignment to interventions |
| Incomplete outcome data (attrition bias) | Unclear | ITT, missing data handled by Maximum Likehood stimation, no sensitivity analysis |
| Selective reporting (reporting bias) | Low | All outcomes reported |
| Other bias | Unclear | Pilot study, small sample size |
| **Turcott 2018** |  |  |
| **Bias** | **Author’s judgement** | **Support for judgement** |
| Random sequence generation (selection bias) | Low | Randomization by the protocol coordinator |
| Allocation concealment (selection bias) | Unclear | No information |
| Blinding of participants and personnel (performance bias) | Unclear | No information |
| Blinding of outcome assessment (detection bias) | Unclear | No information |
| Incomplete outcome data (attrition bias) | Low | Droupouts due to death or clinical deterioration |
| Selective reporting (reporting bias) | Unclear | PG-SGA not reported |
| Other bias | Unclear | Pilot study, small sample size |
| **Turcotte 2015** |  |  |
| **Bias** | **Author’s judgement** | **Support for judgement** |
| Random sequence generation (selection bias) | Low | Computer generated |
| Allocation concealment (selection bias) | Low | By independent pharmacist. Patients, study investigators and trating psysicians were blinded to the assigments |
| Blinding of participants and personnel (performance bias) | Low | There was no information on the label to indicate which treatments patients were receiving |
| Blinding of outcome assessment (detection bias) | Low | There was no information on the label to indicate which treatments patients were receiving |
| Incomplete outcome data (attrition bias) | Low | Missing data imputed separately by calculating the the midpoint of the average of preceding and following day |
| Selective reporting (reporting bias) | Low | All outcomes reported |
| Other bias | Unclear | Small sample size |
| **Ungerleider 1982** |  |  |
| **Bias** | **Author’s judgement** | **Support for judgement** |
| Random sequence generation (selection bias) | Low | Table of random numbers assigned by the pharmacist |
| Allocation concealment (selection bias) | Unclear | No information |
| Blinding of participants and personnel (performance bias) | Unclear | No information |
| Blinding of outcome assessment (detection bias) | Unclear | No information |
| Incomplete outcome data (attrition bias) | Unclear | 75 droupouts from 214, analyzed separately |
| Selective reporting (reporting bias) | Low | All outcomes reported |
| Other bias | Unclear | Unusual analyses, divided in 3 groups |
| **Ungerleider 1987** |  |  |
| **Bias** | **Author’s judgement** | **Support for judgement** |
| Random sequence generation (selection bias) | Unclear | No information |
| Allocation concealment (selection bias) | Unclear | No information |
| Blinding of participants and personnel (performance bias) | Unclear | No information |
| Blinding of outcome assessment (detection bias) | Unclear | No information |
| Incomplete outcome data (attrition bias) | Unclear | No clear description of droputs or missing data |
| Selective reporting (reporting bias) | Unclear | Patients rating results n=2, while physician rating n=8 |
| Other bias | Unclear | Pilot study, small sample size |
| **Van Amerongen 2018** |  |  |
| **Bias** | **Author’s judgement** | **Support for judgement** |
| Random sequence generation (selection bias) | Low | By independent statistician not involved in the execution of the study |
| Allocation concealment (selection bias) | Low | Sealed envelopes for code breaking were available for investigators |
| Blinding of participants and personnel (performance bias) | Low | All staff involved in the clinical execution of the study were blinded until all data were collected and database was locked |
| Blinding of outcome assessment (detection bias) | Low | All staff involved in the clinical execution of the study were blinded until all data were collected and database was locked |
| Incomplete outcome data (attrition bias) | Low | All patients analyzed |
| Selective reporting (reporting bias) | Low | All outcomes reported |
| Other bias | Unclear | Small sample size |
| **Van den Elsen 2015** |  |  |
| **Bias** | **Author’s judgement** | **Support for judgement** |
| Random sequence generation (selection bias) | Low | Computer generated |
| Allocation concealment (selection bias) | Low | Allocation sequence concealed from participants, caregivers, investigators, and all other personnel directly involved in the study |
| Blinding of participants and personnel (performance bias) | Low | Placebo tablets were matched to the active treatment for weight, taste, color and size |
| Blinding of outcome assessment (detection bias) | Low | Treatment allocations were not made available unti study completion and database lock |
| Incomplete outcome data (attrition bias) | Low | Data of all subjects was used in the analysuis without imputation |
| Selective reporting (reporting bias) | Low | All outcomes reported, period effects shown |
| Other bias | Unclear | Small sample size, baseline NPI were higher in the ambulatory and hospital groups |
| **Van den Elsen 2015b** |  |  |
| **Bias** | **Author’s judgement** | **Support for judgement** |
| Random sequence generation (selection bias) | Low | Computer generated |
| Allocation concealment (selection bias) | Low | Allocation sequence concealed from participants, caregivers, investigators, and all other personnel directly involved in the study |
| Blinding of participants and personnel (performance bias) | Low | Matched placebo tablets |
| Blinding of outcome assessment (detection bias) | Low | Treatment allocations were not made available unti study completion and database lock |
| Incomplete outcome data (attrition bias) | Unclear | ITT, no description of missing data handling |
| Selective reporting (reporting bias) | Low | All outcomes reported |
| Other bias | Unclear | Fewer than expected patients had clnically relevant NPS and pain |
| **Vaney 2004** |  |  |
| **Bias** | **Author’s judgement** | **Support for judgement** |
| Random sequence generation (selection bias) | Low | Computer generated by the trial statistician |
| Allocation concealment (selection bias) | Unclear | No information |
| Blinding of participants and personnel (performance bias) | Low | Placebo capsules were identical in shape, taste and colour |
| Blinding of outcome assessment (detection bias) | Unclear | No information |
| Incomplete outcome data (attrition bias) | Unclear | ITT, no period data or no carry over test |
| Selective reporting (reporting bias) | Low | All outcomes reported |
| Other bias | Unclear | No sample size calculation |
| **Vela 2021** |  |  |
| **Bias** | **Author’s judgement** | **Support for judgement** |
| Random sequence generation (selection bias) | Low | Computer generated |
| Allocation concealment (selection bias) | Low | blocks |
| Blinding of participants and personnel (performance bias) | Low | Allocation concelaed from patientss and investigators |
| Blinding of outcome assessment (detection bias) | Low | Allocation concelaed from patientss and investigators |
| Incomplete outcome data (attrition bias) | Low | ITT, sensitivity analyses |
| Selective reporting (reporting bias) | Low | All outcomes reported |
| Other bias | Low |  |
| **Volicer 1997** |  |  |
| **Bias** | **Author’s judgement** | **Support for judgement** |
| Random sequence generation (selection bias) | Unclear | No information |
| Allocation concealment (selection bias) | Unclear | No information |
| Blinding of participants and personnel (performance bias) | Unclear | Identical capsules but the staff knew the objectives of the study |
| Blinding of outcome assessment (detection bias) | High | Although the study was double-blind, the staff knew the objectives of the study |
| Incomplete outcome data (attrition bias) | Low | data from 12 out of 15 participants |
| Selective reporting (reporting bias) | Low | No selective reporting, period effects shown |
| Other bias | Unclear | Small sample size |
| **Wada 1982** |  |  |
| **Bias** | **Author’s judgement** | **Support for judgement** |
| Random sequence generation (selection bias) | Unclear | No information |
| Allocation concealment (selection bias) | Unclear | No information |
| Blinding of participants and personnel (performance bias) | Low | Identical capsules |
| Blinding of outcome assessment (detection bias) | Unclear | No information |
| Incomplete outcome data (attrition bias) | Unclear | No information about missing data handling |
| Selective reporting (reporting bias) | Low | All outcomes are reported, period analysis included |
| Other bias | Low |  |
| **Wade 2004** |  |  |
| **Bias** | **Author’s judgement** | **Support for judgement** |
| Random sequence generation (selection bias) | Low | By permuted blocks of size four |
| Allocation concealment (selection bias) | Low | The pharmacist assigned the treatments in sequential patient number order from the randomization list |
| Blinding of participants and personnel (performance bias) | Low | Peppermint flavour and colouring to disguise the taste and appearance |
| Blinding of outcome assessment (detection bias) | Low | Assessments was undertaken by a research nurse who was not involved in dosing advice and home contact with that patient, to ensure blinding |
| Incomplete outcome data (attrition bias) | Unclear | No information about missing data handling |
| Selective reporting (reporting bias) | Low | All outcomes reported |
| Other bias | Low |  |
| **Ware 2010** |  |  |
| **Bias** | **Author’s judgement** | **Support for judgement** |
| Random sequence generation (selection bias) | Low | Computer generated |
| Allocation concealment (selection bias) | Low | A coded script was given to the subject with instructions on the use of the allocated treatment |
| Blinding of participants and personnel (performance bias) | Low | Subjects were blinded to the allocated treatment order |
| Blinding of outcome assessment (detection bias) | Low | The study physician and study nurse were blinded to the allocated treatment order |
| Incomplete outcome data (attrition bias) | Low | Data are available for all patients, period analysis included |
| Selective reporting (reporting bias) | Low | All outcomes reported |
| Other bias | Unclear | Small sample size |
| **Weber 2010** |  |  |
| **Bias** | **Author’s judgement** | **Support for judgement** |
| Random sequence generation (selection bias) | Low | Computer generated |
| Allocation concealment (selection bias) | Unclear | No information |
| Blinding of participants and personnel (performance bias) | Low | All investigators and patients were blinded to treatment allocation |
| Blinding of outcome assessment (detection bias) | Low | All investigators and patients were blinded to treatment allocation for the duration of the study |
| Incomplete outcome data (attrition bias) | Low | ITT, drop outs not related to treaments |
| Selective reporting (reporting bias) | Low | All outcomes reported, period effects shown |
| Other bias | High | Small sample size, THC effects persisted during the washout and placebo periods |
| **Weizman 2018** |  |  |
| **Bias** | **Author’s judgement** | **Support for judgement** |
| Random sequence generation (selection bias) | Low | By a physician |
| Allocation concealment (selection bias) | Low | Selaed envelope website |
| Blinding of participants and personnel (performance bias) | Unclear | No information |
| Blinding of outcome assessment (detection bias) | Unclear | No information |
| Incomplete outcome data (attrition bias) | Unclear | Data from all participants, no period analysis |
| Selective reporting (reporting bias) | Low | All outcomes reported |
| Other bias | Unclear | Laboratory study, small sample size |
| **Wissel 2006** | | |
| **Bias** | **Author’s judgement** | **Support for judgement** |
| Random sequence generation (selection bias) | Unclear | No information |
| Allocation concealment (selection bias) | Unclear | No information |
| Blinding of participants and personnel (performance bias) | Low | Identical color and taste capsules |
| Blinding of outcome assessment (detection bias) | Unclear | No information |
| Incomplete outcome data (attrition bias) | Unclear | 2 droup outs in nabilone. No bias analysis |
| Selective reporting (reporting bias) | Unclear | Not complete numerical data |
| Other bias | Unclear | Small sample size |
| **Wong 2011** | | |
| **Bias** | **Author’s judgement** | **Support for judgement** |
| Random sequence generation (selection bias) | Low | Computer generated |
| Allocation concealment (selection bias) | Low | Allocation was concealed |
| Blinding of participants and personnel (performance bias) | Low | Participant and investigators were blinded to all treatment assignments |
| Blinding of outcome assessment (detection bias) | Low | At study completion the randomization code was communicated to the study statistician |
| Incomplete outcome data (attrition bias) | Low | ITT. Missing data had the corresponding values imputed using the overall subjects mean |
| Selective reporting (reporting bias) | Low | All outcomes reported |
| Other bias | Low |  |
| **Zadikoff 2011** |  |  |
| **Bias** | **Author’s judgement** | **Support for judgement** |
| Random sequence generation (selection bias) | Low | Computer generated |
| Allocation concealment (selection bias) | Low | Numbers table remained withing the hospital pharmacy until the end of the trial |
| Blinding of participants and personnel (performance bias) | Low | Matching placebo |
| Blinding of outcome assessment (detection bias) | Unclear | No information |
| Incomplete outcome data (attrition bias) | Low | Data from each period analyzed |
| Selective reporting (reporting bias) | Low | All outcomes reported |
| Other bias | Unclear | Small sample size |
| **Zajicek 2003/Freeman 2006** | | |
| **Bias** | **Author’s judgement** | **Support for judgement** |
| Random sequence generation (selection bias) | Low | Adaptative randomisaton, dedicated stand-alone computer |
| Allocation concealment (selection bias) | Low | Treament allocation codes was kept at the central trial pharmacy |
| Blinding of participants and personnel (performance bias) | Low | No obvious difference between treatments |
| Blinding of outcome assessment (detection bias) | Low | The study coordinating team, all investigators, the data monitoirng comittee, and patinets were inaware of the treatment allocation for the duration of the study |
| Incomplete outcome data (attrition bias) | Low | ITT of 97% participants |
| Selective reporting (reporting bias) | Low | All outcomes reported |
| Other bias | Low |  |
| **Zajicek 2013/Ball 2015** | | |
| **Bias** | **Author’s judgement** | **Support for judgement** |
| Random sequence generation (selection bias) | Low | Computer generated |
| Allocation concealment (selection bias) | Low | Assigment of treatment independent of research team |
| Blinding of participants and personnel (performance bias) | Low | Participants and all other personnel directly involved in the study were masked to treatment allocation |
| Blinding of outcome assessment (detection bias) | Low | Participants and all other personnel directly involved in the study were masked to treatment allocation |
| Incomplete outcome data (attrition bias) | Low | ITT, missing data as censored observations, sensitivity analysis |
| Selective reporting (reporting bias) | Low | All outcomes reported |
| Other bias | Low |  |

**Supplementary Figure 1. Risk of bias summary for dronabinol**

**
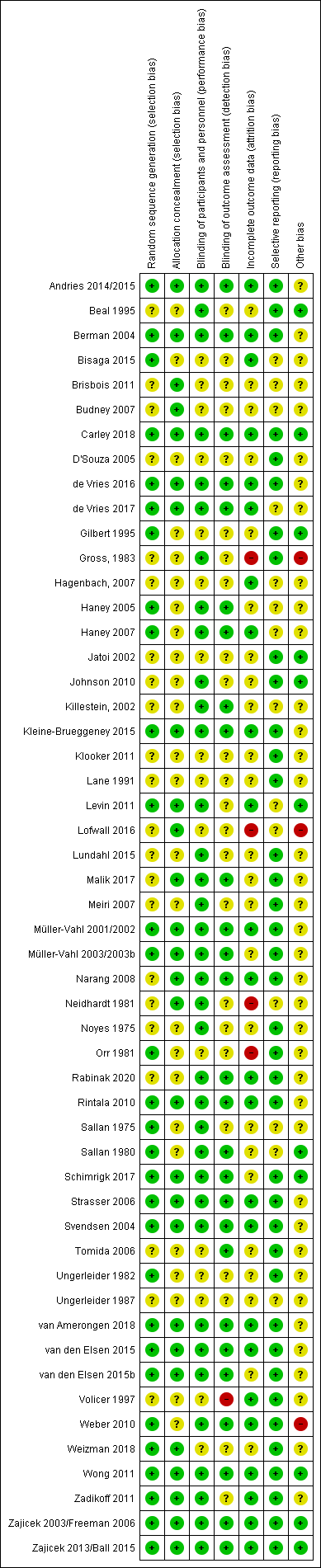
**

**Supplementary Figure 2. Risk of bias graph for dronabinol**

**
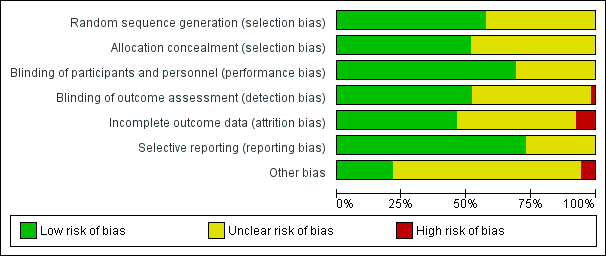
**

**Supplementary Figure 3. Risk of bias summary for nabilone**


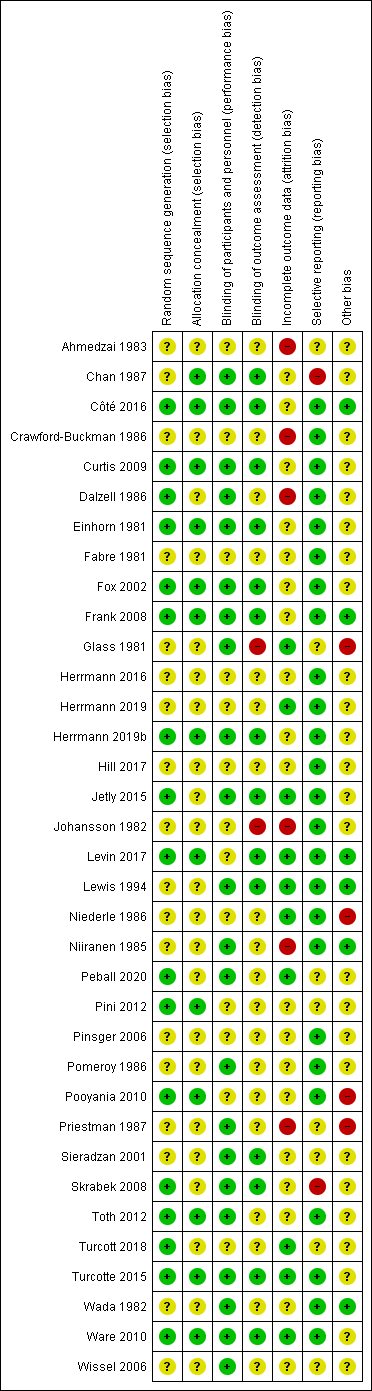


**Supplementary Figure 4. Risk of bias graph for nabilone**


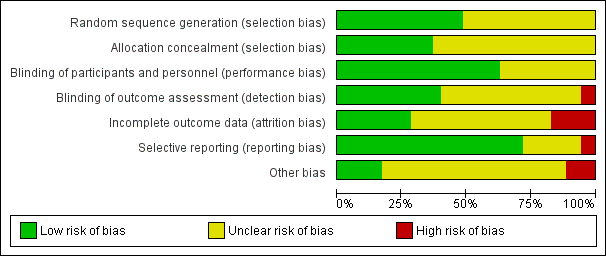


**Supplementary Figure 5. Risk of bias summary for CBD**


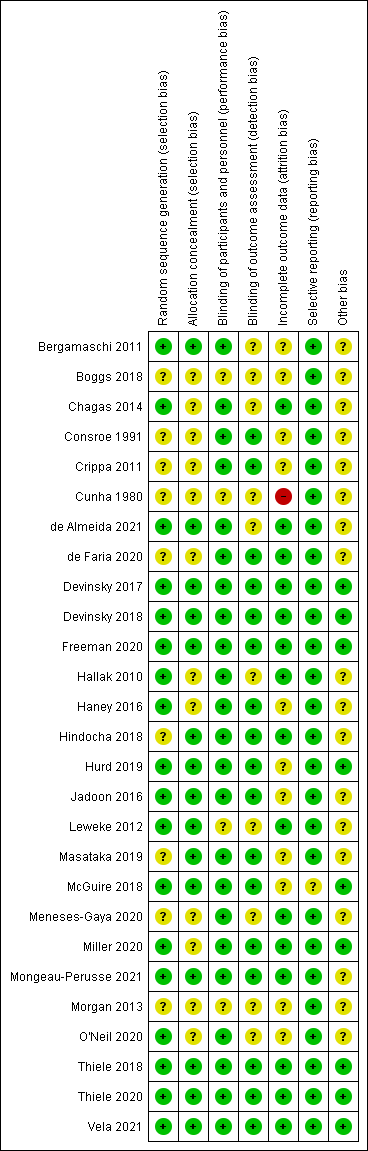


**Supplementary Figure 6. Risk of bias graph for CBD**


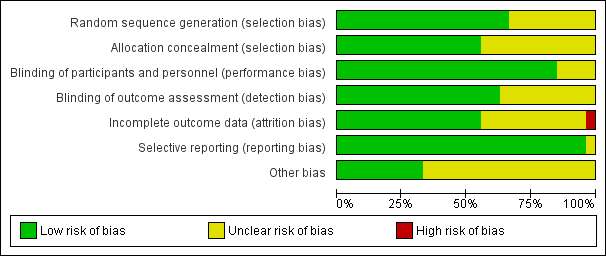


**Supplementary Figure 7. Risk of bias summary for nabiximols**

**
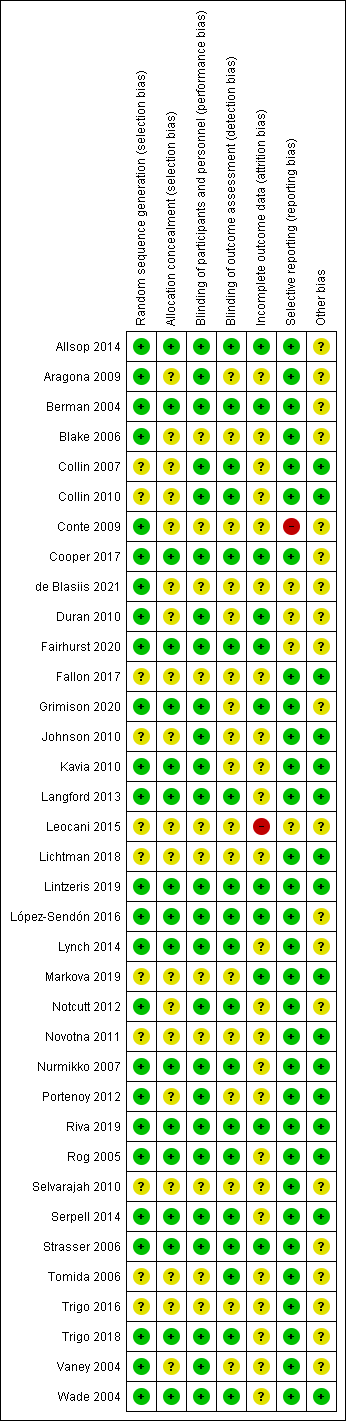
**

**Supplementary Figure 8. Risk of bias graph for nabiximols**

**
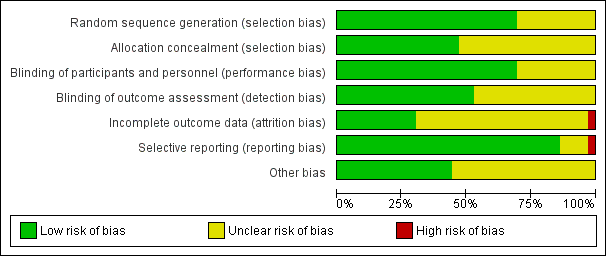
**
