# Supplementary material for: Medical cannabinoids: a pharmacology-based systematic review and meta-analysis for all relevant medical indications
Source: BMC Med. 2022 Aug 19;20:259. doi: 10.1186/s12916-022-02459-1 (PMC9389720; doi:10.1186/s12916-022-02459-1)
Supplement: Supplementary file 4 — Additional file 4. Forest-plot for primary outcomes. [file 12916_2022_2459_MOESM4_ESM.docx]

**Forest plots for relevant medical conditions (primary outcomes) stratified by type of cannabinoids**

**Supplementary Figure 9. Forest plot for nausea and vomiting**

**
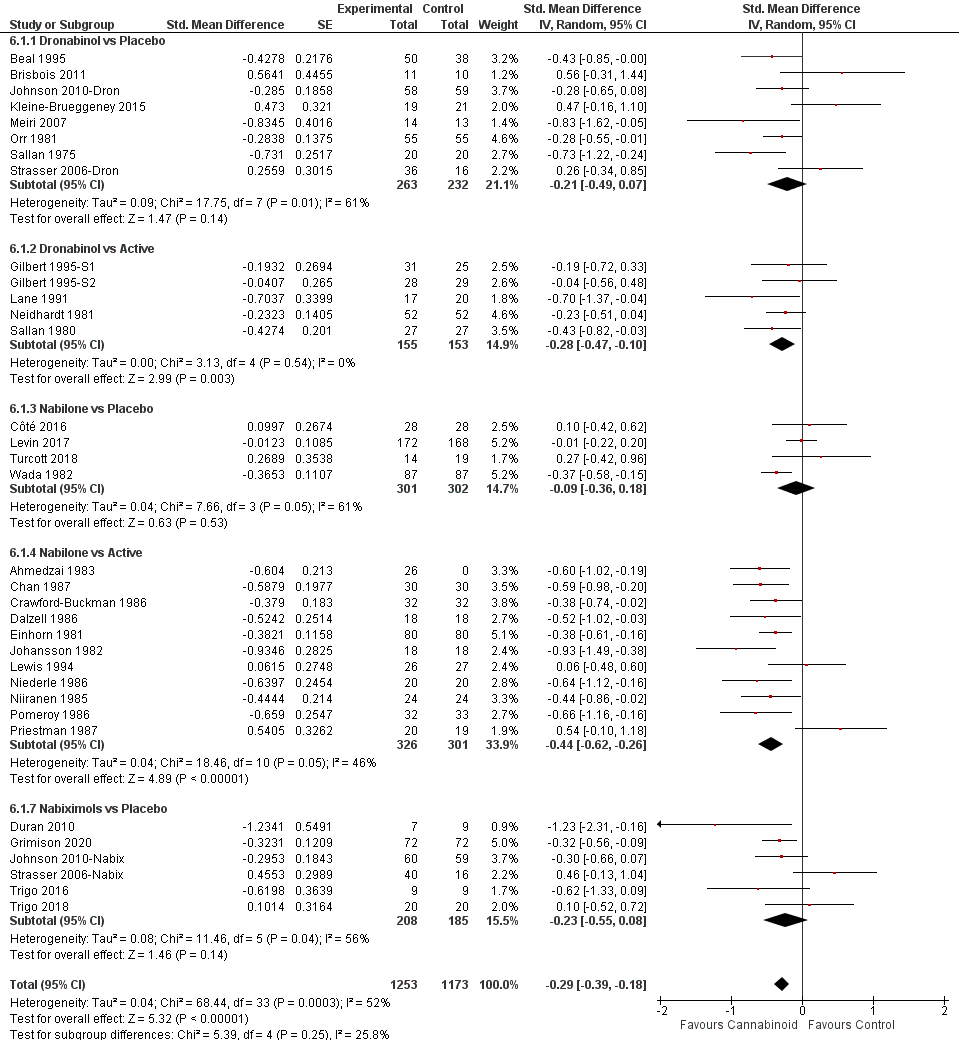
**

**Supplementary Figure 10. Forest plot for amyotrophic lateral sclerosis**


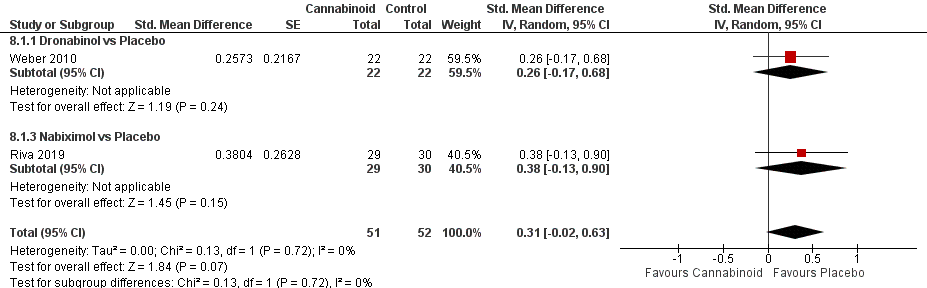


**Supplementary Figure 11. Forest plot for Chorea Huntington**


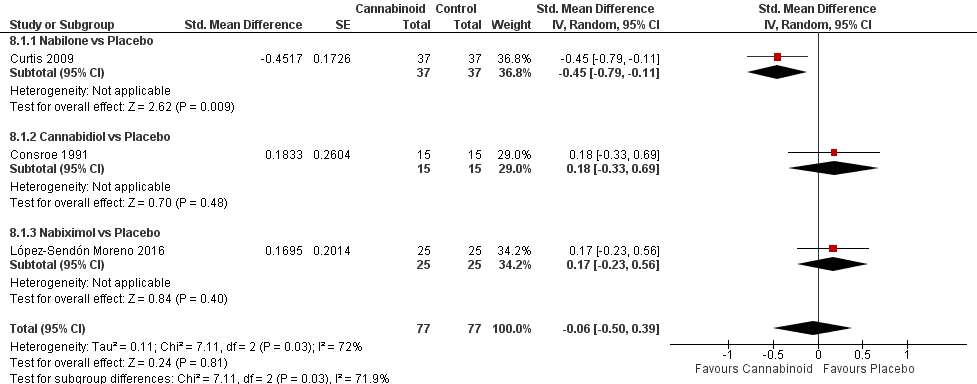


**Supplementary Figure 12. Forest plot for dystonia**

**
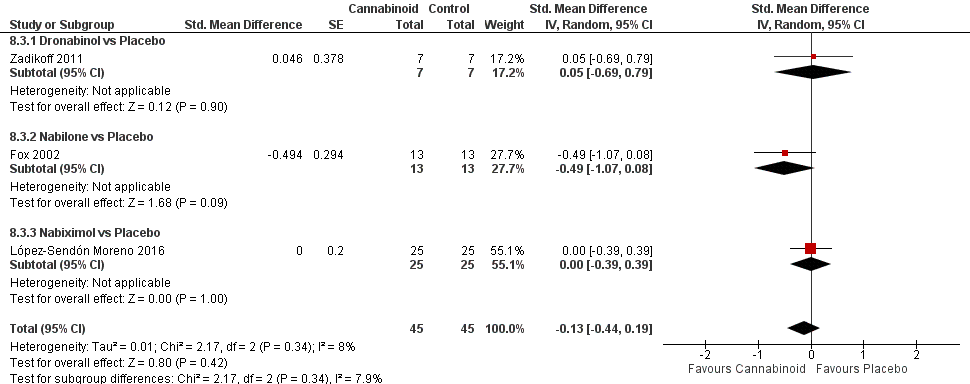
**

**Supplementary Figure 13. Forest plot for glaucoma**


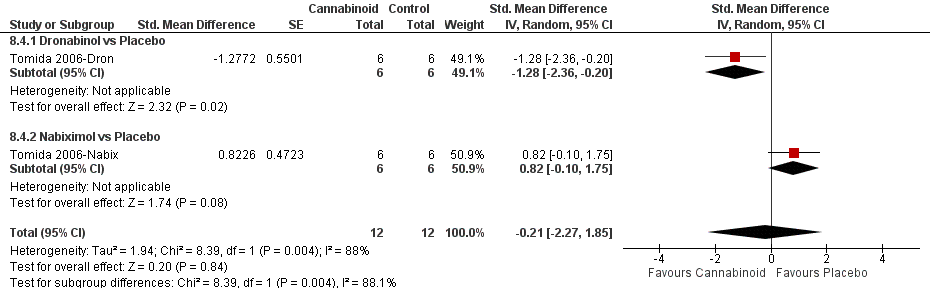


**Supplementary Figure 14. Forest plot for irritable bowel syndrome**


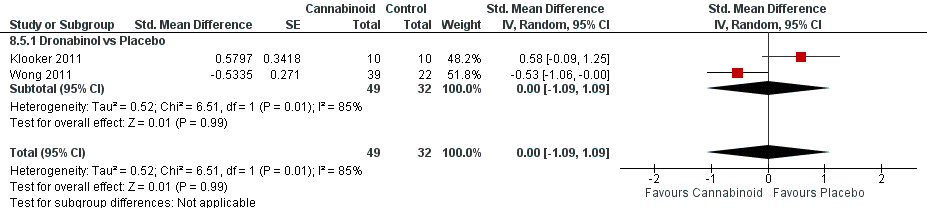


**Supplementary Figure 15. Forest plot for multiple sclerosis**


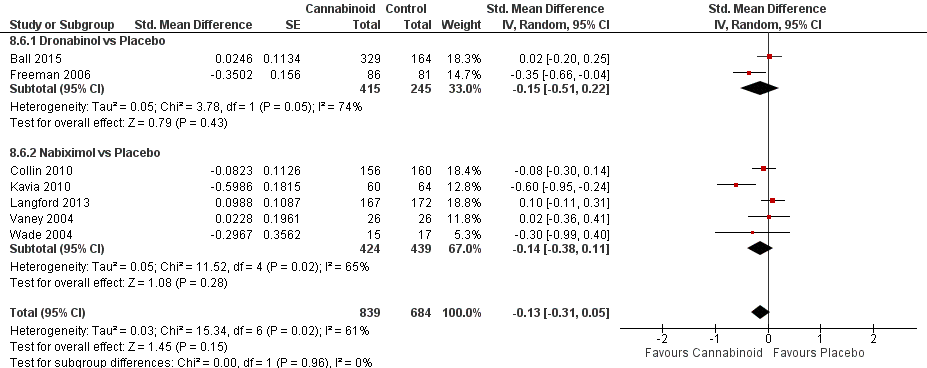


**Supplementary Figure 16. Forest plot for anorexia nervosa**


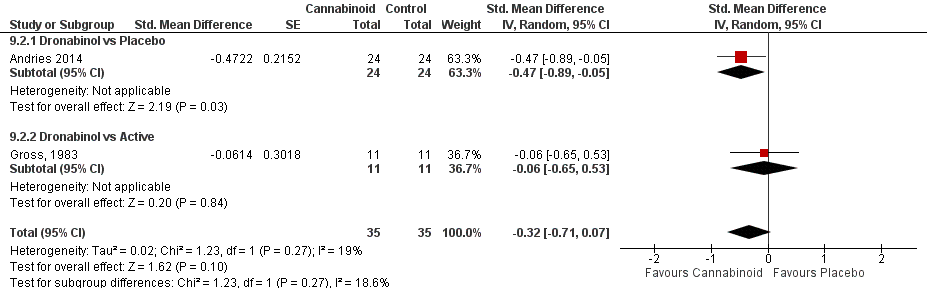


**Supplementary Figure 17. Forest plot for anxiety**

**
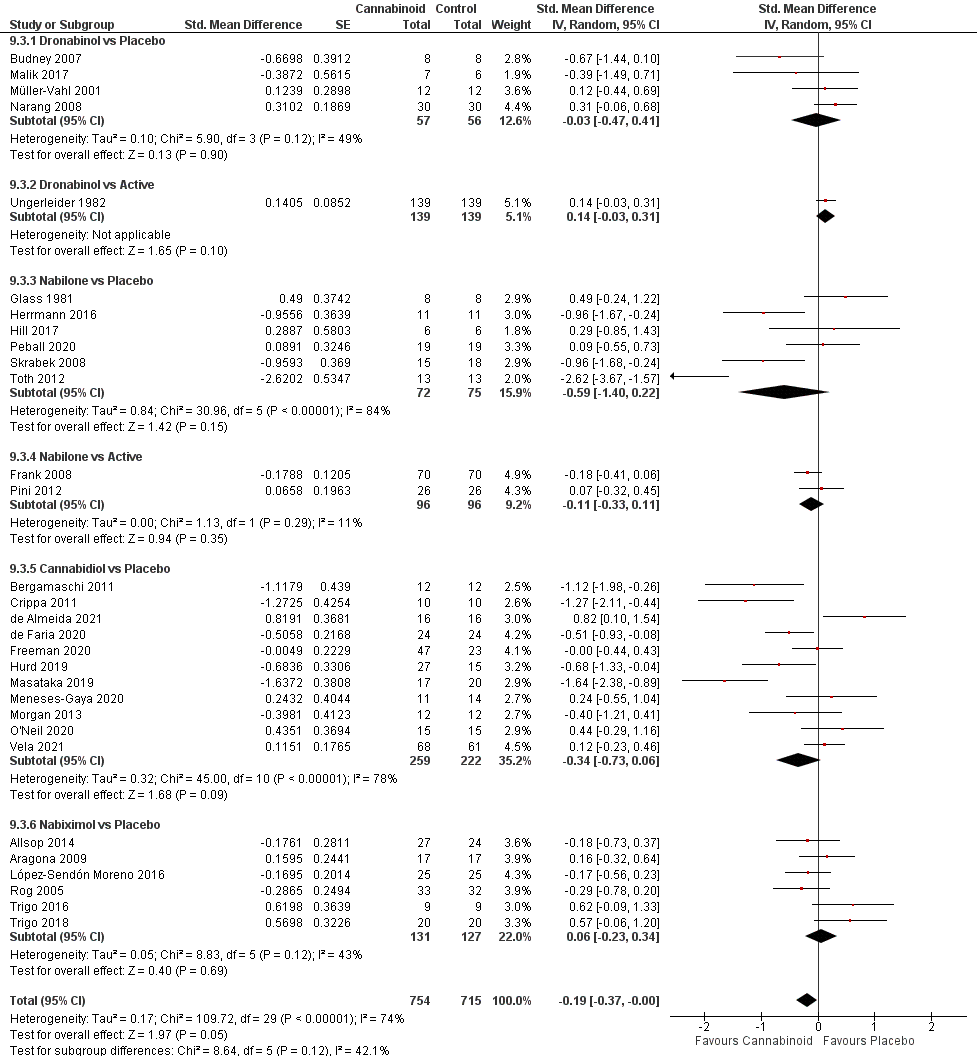
**

**Supplementary Figure 18. Forest plot for dementia**

**
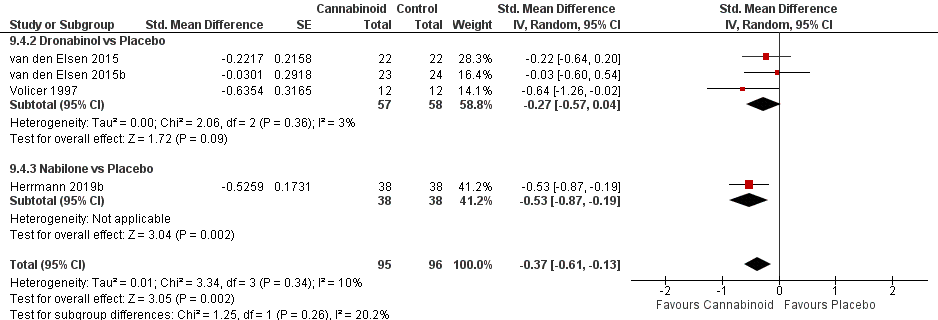
**

**Supplementary Figure 19. Forest plot for depression**

**
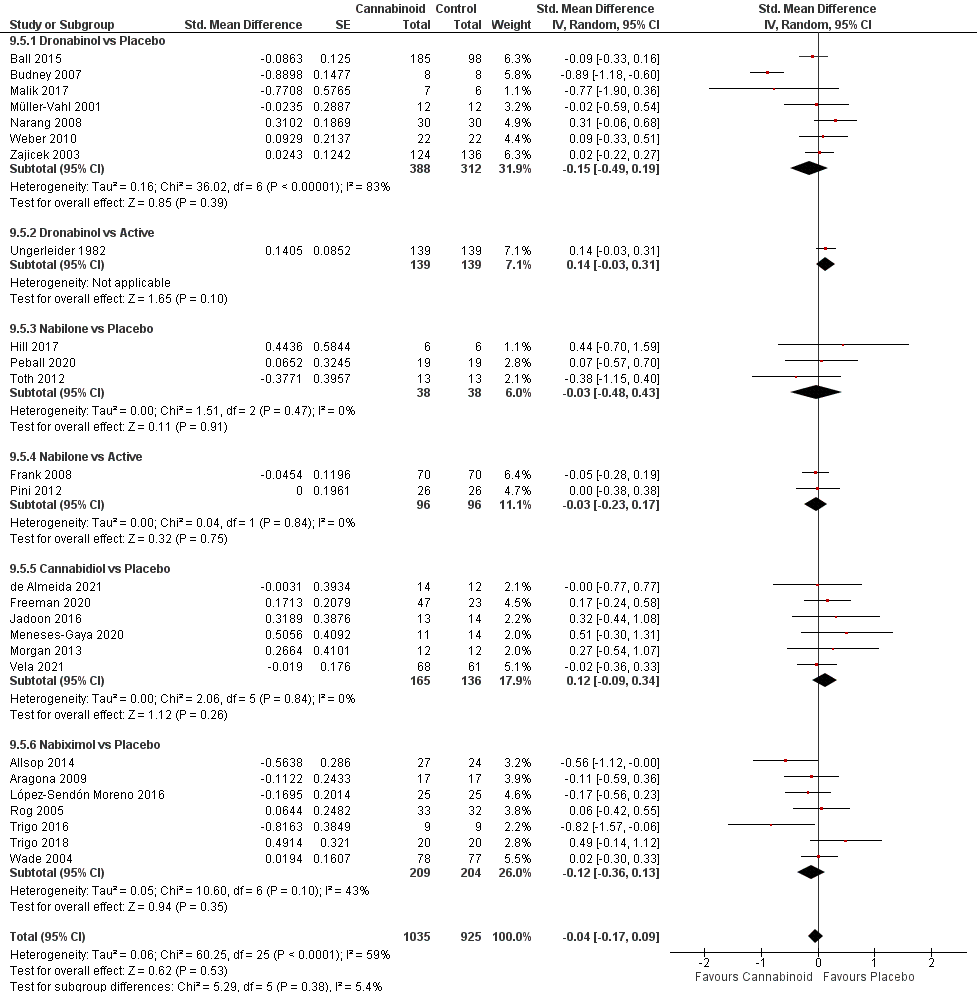
**

**Supplementary Figure 20. Forest plot for PTSD**

**
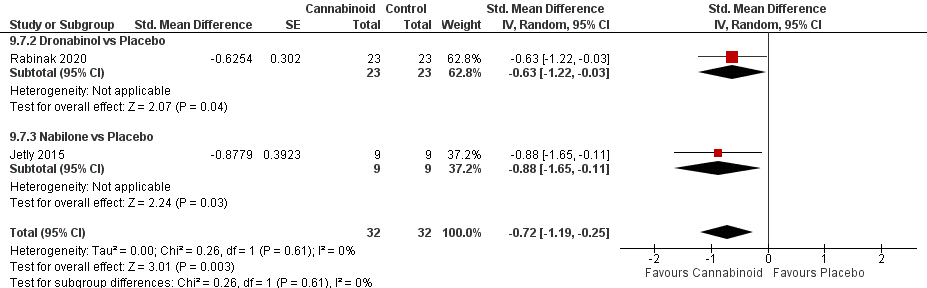
**

**Supplementary Figure 21. Forest plot for schizophrenia and psychosis**

**
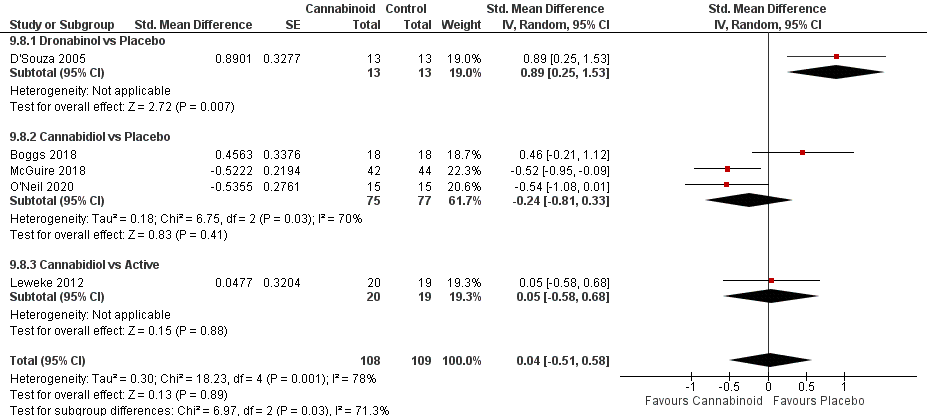
**
