# Supplementary material for: Medical cannabinoids: a pharmacology-based systematic review and meta-analysis for all relevant medical indications
Source: BMC Med. 2022 Aug 19;20:259. doi: 10.1186/s12916-022-02459-1 (PMC9389720; doi:10.1186/s12916-022-02459-1)
Supplement: Supplementary file 5 — Additional file 5. Meta-regression analysis. [file 12916_2022_2459_MOESM5_ESM.docx]

**Supplementary Table 7. Meta-regression results**

| **OUTCOME** | **COMPARISON** | **Q** | **df** | **p** | **coefficient** | **CI 95%** |
| --- | --- | --- | --- | --- | --- | --- |
| Chronic Pain | Nabilone vs Dronabinol | 0,01 | 1 | 0,9127 | -0,0168 | -0,3178 to 0,2842 |
|  | Nabilone vs Nabiximols | 0,43 | 1 | 0,5137 | 0,0941 | -0,1884 to 0,3766 |
|  | Nabiximols vs Dronabinol | 0,58 | 1 | 0,4468 | 0,0740 | -0,1166 to 0,2646 |
| Nausea | Dronabinol (active) vs Nabilone (active) | 1,90 | 1 | 0,1677 | -0,1562 | -0,3781 to 0,0657 |
| Parkinson | Nabilone vs CBD | 0 | 1 | 0,9598 | 0,0154 | -0,5834 to 0,6142 |
| Dementia | Dronabinol vs Nabilone | 1,28 | 1 | 0,2580 | -0,2607 | -0,7123 to 0,1910 |
| Sleep | Nabiximols vs Nabilone | 1,96 | 1 | 0,1618 | 0,2444 | -0,0980 to 0,5868 |
| SUD | Dronabinol (placebo) vs Dronabinol (active) | 1,47 | 1 | 0,2253 | -0,3826 | -1,0010 to 0,2358 |
|  | Dronabinol vs Nabilone | 0,14 | 1 | 0,7044 | -0,0886 | -0,5466 to 0,3693 |
|  | Dronabinol vs Nabiximols | 0,01 | 1 | 0,9272 | -0,0173 | -0,3874 to 0,3529 |
|  | Dronabinol (active) vs Nabilone (placebo) | 0,74 | 1 | 0,3902 | 0,2939 | -0,3766 to 0,9645 |
|  | Dronabinol (active) vs Nabiximols | 1,36 | 1 | 0,2435 | 0,3653 | -0,2486 to 0,9793 |
|  | Nabilone (placebo) vs Nabiximols | 0,10 | 1 | 0,7569 | 0,0714 | -0,3805 to 0,5233 |
| Adverse events | Dronabinol vs. CBD | 0,50 | 1 | 0,4805 | 0,2068 | -0,3677 to 0,7813 |
|  | Nabiximols vs CBD | 0,04 | 1 | 0,8424 | 0,0583 | -0,5161 to 0,6327 |
|  | Nabilone vs CBD | 1,22 | 1 | 0,2701 | 0,4590 | -0,3567 to 1,2748 |
