# Supplementary material for: Medical cannabinoids: a pharmacology-based systematic review and meta-analysis for all relevant medical indications
Source: BMC Med. 2022 Aug 19;20:259. doi: 10.1186/s12916-022-02459-1 (PMC9389720; doi:10.1186/s12916-022-02459-1)
Supplement: Supplementary file 6 — Additional file 6. Forest-plot for secondary outcomes: retention and adverse events. [file 12916_2022_2459_MOESM6_ESM.docx]

**Forest plots for secondary outcomes stratified by type of cannabinoids**

**Supplementary Figure 22. Forest plot for retention**

**
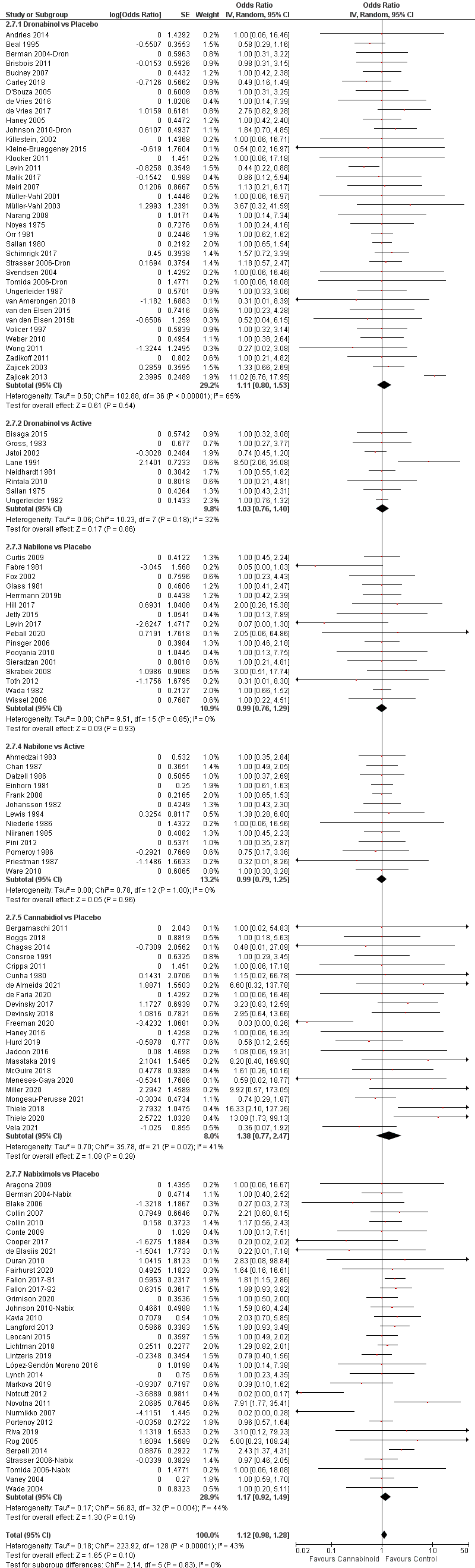
**

**Supplementary Figure 23. Forest plot for adverse events**

**
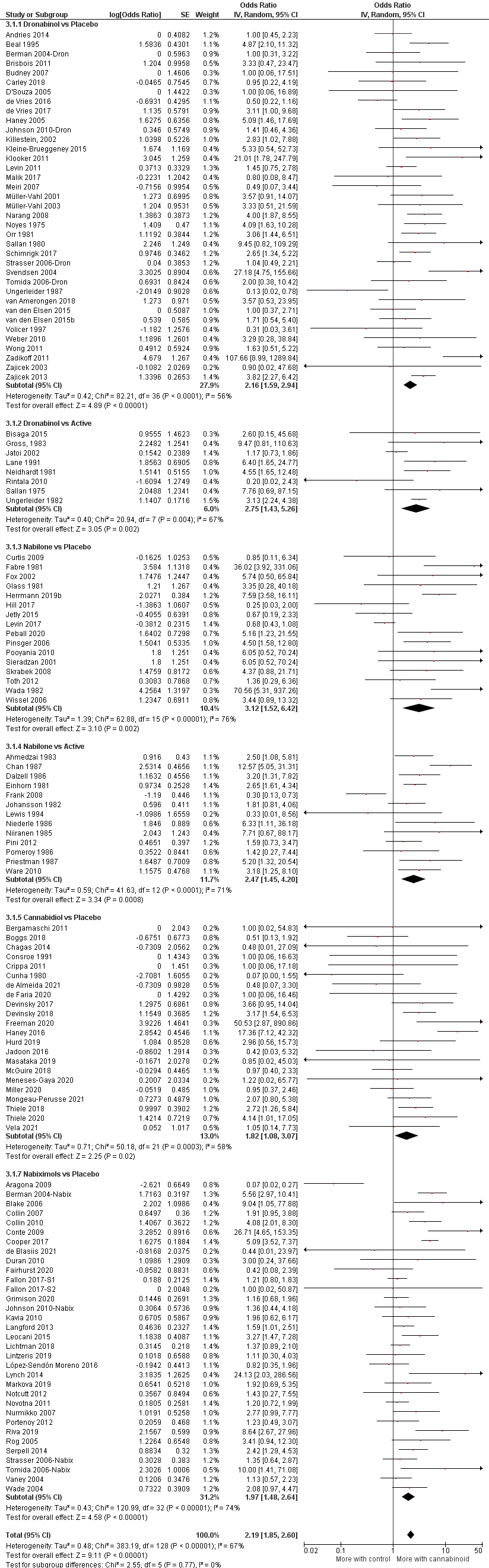
**
